# Supplementary material for: Loop detection using Hi-C data with HiCExplorer
Source: Gigascience. 2022 Jul 9;11:giac061. doi: 10.1093/gigascience/giac061 (PMC9270730; doi:10.1093/gigascience/giac061)
Supplement: giac061_GIGA-D-21-00069_Original_Submission [file giac061_giga-d-21-00069_original_submission.pdf]

GigaScience
Loop detection using Hi-C data with HiCExplorer
--Manuscript Draft--

**Manuscript Number:**	GIGA-D-21-00069
**Full Title:**	Loop detection using Hi-C data with HiCExplorer
**Article Type:**	Technical Note
**Funding Information:**	Bundesministerium für Bildung, Wissenschaft, Forschung und Technologie (031 A538A de.NBI-RBC) — Prof. Dr. Rolf Backofen
	Bundesministerium für Bildung, Wissenschaft, Forschung und Technologie (031 L0101C de.NBI-epi) — Dr. Björn Grüning
	Deutsche Forschungsgemeinschaft (CIBSS - EXC-2189 - Project ID 390939984) — Prof. Dr. Rolf Backofen
**Abstract:**	Background: Chromatin loops are an essential factor in the structural organization of the genome. The detection of chromatin loops in Hi-C interaction matrices is a challenging and compute-intensive task. The presented approach shows a chromatin loop detection algorithm that applies a strict candidate selection based on continuous negative binomial distributions and performs a Wilcoxon rank-sum test to detect enriched Hi-C interactions.<br>Results: HiCExplorers loop detection has a high detection rate and accuracy while providing specificity. It is the fastest available CPU implementation and utilizes all threads offered by modern multi-core platforms.<br>Conclusions: HiCExplorers method to detect loops by using a continuous negative binomial function combined with the donut approach from HiCCUPS leads to reliable and fast computation of loops. All investigated loop-calling algorithms provide a differing number of detect loops and intersect in the best cases by ~50%. The tested in-situ Hi-C data contains high amounts of noise; more similar results in loop calling requires cleaner Hi-C data and therefore, improvements in the data creation.
**Corresponding Author:**	Joachim Wolff<br>Albert-Ludwigs-Universität Freiburg: Albert-Ludwigs-Universitat Freiburg<br>Freiburg im Breisgau, Baden-Württemberg GERMANY
**Corresponding Author Secondary Information:**	
**Corresponding Author's Institution:**	Albert-Ludwigs-Universität Freiburg: Albert-Ludwigs-Universitat Freiburg
**Corresponding Author's Secondary Institution:**	
**First Author:**	Joachim Wolff
**First Author Secondary Information:**	
**Order of Authors:**	Joachim Wolff
	Rolf Backofen, Dr.
	Björn Grüning, Dr.
**Order of Authors Secondary Information:**	
**Additional Information:**	
**Question**	**Response**
Are you submitting this manuscript to a special series or article collection?	No
**Experimental design and statistics**	Yes

Powered by Editorial Manager® and ProduXion Manager® from Aries Systems Corporation

Full details of the experimental design and statistical methods used should be given in the Methods section, as detailed in our Minimum Standards Reporting Checklist. Information essential to interpreting the data presented should be made available in the figure legends.<br><br>Have you included all the information requested in your manuscript?	
**Resources**<br><br>A description of all resources used, including antibodies, cell lines, animals and software tools, with enough information to allow them to be uniquely identified, should be included in the Methods section. Authors are strongly encouraged to cite Research Resource Identifiers (RRIDs) for antibodies, model organisms and tools, where possible.<br><br>Have you included the information requested as detailed in our Minimum Standards Reporting Checklist?	Yes
**Availability of data and materials**<br><br>All datasets and code on which the conclusions of the paper rely must be either included in your submission or deposited in publicly available repositories (where available and ethically appropriate), referencing such data using a unique identifier in the references and in the "Availability of Data and Materials" section of your manuscript.<br><br>Have you have met the above requirement as detailed in our Minimum Standards Reporting Checklist?	Yes

Powered by Editorial Manager® and ProduXion Manager® from Aries Systems Corporation

Click here to access/download;Manuscript;Loop_detection_manuscript.pdf

OXFORD (GIGA)n SCIENCE

GigaScience, XXXX, 1–7

doi: xx.xxxx/xxxx
Manuscript in Preparation
Technical Note

TECHNICAL NOTE

Loop detection using Hi-C data with HiCExplorer

Joachim Wolff[1,*], Rolf Backofen[1,2] and Björn Grüning[1]

[1]Bioinformatics Group, Department of Computer Science, University of Freiburg, Georges-Köhler-Allee 106, 79110 Freiburg, Germany and [2]Signalling Research Centres CIBSS, University of Freiburg, Schänzlestr. 18, 79104 Freiburg, Germany

[*]wolffj@informatik.uni-freiburg.de

Abstract

Background: Chromatin loops are an essential factor in the structural organization of the genome. The detection of chromatin loops in Hi-C interaction matrices is a challenging and compute-intensive task. The presented approach shows a chromatin loop detection algorithm that applies a strict candidate selection based on continuous negative binomial distributions and performs a Wilcoxon rank-sum test to detect enriched Hi-C interactions. **Results:** HiCExplorers loop detection has a high detection rate and accuracy while providing specificity. It is the fastest available CPU implementation and utilizes all threads offered by modern multi-core platforms. **Conclusions:** HiCExplorers method to detect loops by using a continuous negative binomial function combined with the donut approach from HiCCUPS leads to reliable and fast computation of loops. All investigated loop-calling algorithms provide a differing number of detect loops and intersect in the best cases by ~ 50%. The tested in-situ Hi-C data contains high amounts of noise; more similar results in loop calling requires cleaner Hi-C data and therefore, improvements in the data creation.

Key words: Hi-C, Hi-C loop detection, DNA loops

Introduction

Chromosome conformation capture (3C) [1] and its successors 4C [2, 3], 5C [4] and Hi-C [5] are protocols to study the three dimensional structure of a genome. With Hi-C data, a genome-wide interaction map of the chromatin can be created, and chromatin loops can be inferred. Chromatin loops reflect the interaction of promoters and enhancers, gene loops, architectural loops, or polycomb-mediated regions [6] and can be detected as enriched regions in comparison to their neighborhood. By identifying these regions, it can be shown that there are long-range regulations that impact, e.g., the directionality of RNA synthesis [7], or the long-distance between cis-regulatory elements [6] that can not be explained and shown otherwise. Based on Rao [8] the genomic distance between two loci is usually limited to ~ 2 megabases (Mb).
Different algorithms can detect loops: HiCCUPS uses a *donut algorithm*, which considers all elements of a Hi-C interaction matrix as peaks and tests if the region around them is significantly different from the neighboring interactions. HiCCUPS is part of the software Juicer[1], and the implementation requires a general-purpose GPU (GPGPU), which imposes a barrier to many users by merely not having access to an Nvidia GPU. However, an experimental CPU-based implementation was released too. HOMER [9] creates a relative contact matrix per chromosome and scans these for locally dense regions. HOMER does not support standard file formats for Hi-C matrices like *cool* [10], which imposes the need to create all data from scratch, which is time-consuming and is a potential source of errors and inaccuracies. Chromosight [11] detects loops based on a pattern-matching algorithm. Cooltools[2] uses a reimplementation of the HiCCUPS algorithm. Chromosight, cooltools and HiCExplorer support the *cooler* file format. GOTHIC [12] models the probability of two genomic locations to interact with each other as a mix of different biases and the chance of random interactions. The problem is that GOTHIC detects a large number of significant interactions but cannot detect only the enriched

1 https://github.com/aidenlab/juicer
2 https://github.com/open2c/cooltools

Compiled on: February 28, 2021.
Draft manuscript prepared by the author.

regions concerning their neighborhood. It is a good tool to detect significant interactions in a Hi-C interaction matrix, but it is not suitable for the specific task of chromatin loop detection. cLoops [13] uses a DBSCAN based approach combined with a local background to estimate the statistical significance of a loop. cLoops is mainly designed for HiChIP data and not for Hi-C. With HiChIP, protein binding sites can be investigated in their 3D context; however, similar to promoter capture Hi-C, only the targeted regions are enriched. The consequence of this is a Hi-C matrix with data only available at these enriched regions, and foreknowledge of potential loop locations is required. FastHiC [14] is a loop detection algorithm based on a hidden Markov random field Bayesian [15], which focuses on intra topological associated domain (TAD) loops in a range of 40kb and therefore not on chromatin loops outside of TADs.

Here we present an algorithm that can detect Hi-C loops. It is optimized for a high parallelization by assigning one thread per chromosome and multiple threads within a chromosome. This approach makes full use of the resources available in the last generation of multi-core CPU platforms.

Methods

According to Rao [8], most of the anchor points of detected loops lie within a range of 2 Mb. This insight can be used to decrease the search space in a biologically meaningful way and also reduces the computational burden, maintaining a low memory footprint at the same time. Moreover, interaction pairs with genomic distances which are too close to each other and therefore quite close to the main diagonal have already high interaction counts. It is in many cases unlikely that these pairs contribute enrichments in the context of their neighborhood. The high interaction count can explain this observation between two loci; they are closer in the one-dimensional space and are therefore close to the main diagonal. Specialized algorithms like FastHiC should be used to detect intra-TAD enrichments. A general problem for Hi-C interactions with few absolute counts is the difficulty to determine if their interactions are true interactions or noise. These artifacts cannot be corrected by the used Hi-C interaction matrix correction algorithms like iterative correction and eigenvector decomposition (ICE) [16], or Knight-Ruiz (KR) [17]. These algorithms perform a matrix balancing and correct for an uneven distribution of the interaction counts per genomic position. The correction algorithms cannot decide and therefore filter out if interactions are true interactions or noise. All values below a given threshold are discarded, and noise is removed to account for these known problems in the Hi-C interaction data.

Algorithm

A strict candidate selection is critical to reduce the computational complexity of the loop detection algorithm. A maximum loop size can be defined to restrict the search space (Figure 1B) to take the observation from Rao into account. In Hi-C, the primary data structure is the symmetrical $n \times n$ interaction count matrix (ICM):

$$ICM = \begin{bmatrix} ic_{00} & \cdots & ic_{0n} \\ \vdots & \ldots & \vdots \\ ic_{n0} & \cdots & ic_{nn} \end{bmatrix} \quad (1)$$

The relative genomic distance is given by:

$$d = |i - j| \; for \; ic_{i,j} \quad (2)$$

And $ic_{i,j}$ as an element of Hi-C interaction matrix ICM.

As a first step, the interaction matrix ICM is transferred to an observed vs. expected matrix to normalize the differing interaction heights per genomic distance. The observed/expected matrix is named M^*. Each entry is defined as:

$$m^*_{i,j} = \frac{icm_{i,j}}{exp_d} \quad (3)$$

Different methods are offered to adjust differences in samples introduced, e.g., by the ligations or by general genome properties, to compute the expected value. A mammal Hi-C sample might need a different normalization compared to an insect.

$$exp\_nonzero_d = \frac{\sum ic_{i,j}}{|non - zero \; interactions \; d|} \quad (4)$$

$$exp\_with\_zero_d = \frac{\sum ic_{i,j}}{|all \; interactions \; d|} \quad (5)$$

$$exp\_ligation_d = exp\_nonzero_{i,j} * \frac{\sum(row_{ICM}(i)) * \sum(row_{ICM}(j))}{\sum(ICM)} \quad (6)$$

Candidate selection per genomic distance

To detect enriched Hi-C interactions, the observed/expected normalized Hi-C data is fitted per genomic distance d independently to a continuous negative binomial distribution (Figure 1C):

$$X_d \sim cNB_d(r_d, p_d) \; \forall d = |i - j| \quad (7)$$

Supplementary Figure 1 shows the value density distribution of different genomic distances and provides evidence for the chosen distribution assumption. In genome analysis, good experience has been made with negative binomial functions as proposed, for example, by DESeq2 [18]. The binomial coefficient must be replaced as it used by edgeR [19, 20] and was discussed at stackexchange[3] to make the discrete negative binomial function continuous:

$$\binom{k + r - 1}{k} = \frac{(k + r - 1)!}{(k!) * (k + r - 1 - k)!} = \frac{(k + r - 1)!}{(k!) * (r - 1)!} \quad (8)$$

The gamma function is defined for any $n \in N$:

$$\Gamma(n) = (n - 1)! \quad (9)$$

Moreover, the gamma function is defined for any $n \in R_{>0}$:

$$\Gamma(n) = \int_0^\infty x^{n-1} * e^{-x} dx \quad (10)$$

3 https://stats.stackexchange.com/questions/310676/continuous-generalization-of-the-negative-binomial-distribution/311927

With Equation (9), the binomial coefficient can be reformulated as:

$$\binom{k+r-1}{k} = \frac{\Gamma(k+r)}{\Gamma(k+1) * \Gamma(r)} \quad (11)$$

Which leads to the probability mass function for a 'continuous negative binomial distribution' with $\forall k \in R_{>0}$ and $\forall r \in R_{>0}$:

$$f(k,r,p) = \frac{\Gamma(k+r)}{\Gamma(k+1) * \Gamma(r)} p^k (1-p)^r \quad (12)$$

The p-value of observing a specific observed vs. expected value at the genomic distance d is given by the continuous negative binomial cumulative density function:

$$pvalue\ of\ m^*_{i,j} = P(x \geq m^*_{i,j}) = \begin{cases} 1 - CDF_d(m^*_{i,j}) & \text{if } m^*_{i,j} > 0. \\ 1 & \text{if i = 0.} \end{cases} \quad (13)$$

Only the observed vs. expected values with p-values smaller than an individual threshold per genomic distance are accepted as candidates (Figure 1D and 1E); these candidates are further filtered to remove candidates with too few absolute interactions). To reduce the amount of data to fit, the user can remove observed vs. expected values lower a threshold before the continuous negative binomial function is fitted. Moreover, an option to remove candidates by their interaction height is given too.

Loop peak detection

The entire neighborhood needs to be considered to detect enriched regions in a Hi-C interaction matrix. A neighborhood is a square of size n with the candidate element in its center; see Figure 1F. An enriched region needs to have an enriched interaction count in relation to the elements in its neighborhood. The concept of a neighborhood comes with a few issues: First, in one neighborhood, there can be multiple candidates detected from different, but next to each other located genomic distances. Second, if a candidate is significant for its genomic distance, it is not necessarily an enriched value for its neighborhood. Third, a single enriched interaction in a neighborhood is possible but is likely a false positive. Meaningful enriched interactions appear in groups and form a peak in the two-dimensional space, as shown in Figure 1F. All candidates in one neighborhood are pooled together to handle the first issue, only the candidate with the highest observed vs. expected value for one neighborhood is considered a representative of its neighborhood; all others are removed. The neighborhood is split into a peak and a background region to cover the second and third issue by considering the square around the candidate as the peak region and the neighborhood's remaining elements as the background, see Figure 1G. The neighborhood is further divided into the vertical region left and right from the peak, the horizontal region above and below the peak, and the bottom left corner; this is a similar approach to HiCCUPS [8]. The peak and neighborhood square sizes are defined by their inradius values, *peakWidth* and *windowSize*. All candidates which fulfill of the following condition are rejected as a loop: $mean(background) \geq mean(peak)$. This filtering step is necessary to address the candidate peak value as a singular outlier within the neighborhood. Furthermore, the Wilcoxon rank-sum test with H0 hypothesis background and peak regions are from the same distribution with significance level p is used. The mentioned filter steps guarantee only neighborhoods with a centering peak value are considered.

Analyses

The algorithm was tested on various cell types published by Rao 2014 to verify the chromatin loop detection algorithm results: GM12878, K562, IMR90, HUVEC, KBM7, NHEK, and HMEC. Additionally, the detected chromatin loop locations are correlated with binned protein peak locations of the 11-zinc finger protein CTCF. CTCF is a known loop binding factor [8] although not all peaks need to have CTCF attached [21], especially in the case of a gene or a polycomb-mediated loop [6]. An intersection of a detected chromatin loop region was accepted if at both loci, CTCF was detected. CTCF was matched to the GM12878, HMEC, HUVEC, K562, and NHEK cell samples; for IMR90 and KBM7, no CTCF from the same source is provided. HiCExplorer's implementation is tested against HiCCUPS algorithm from the Juicer software, HOMER's loop detection, chromosight, and the cooltools *call-dots*. The algorithms of GOTHIC, cLoops, and FastHiC are not part of the comparison due to the algorithms' different focuses.

HiCExplorer candidate selection

The following section was computed on GM12878 with applied Knights-Ruiz correction and a 10kb fixed bin size resolution. The loop detection considers each chromosome independently; data from chromosome 1 shows the search space reduction as an example. The p-value was set to 0.05 for continuous negative binomial distribution candidate selection, a minimal interaction peak height of 20, a peak width of 6, a window size of 10, and a maximal interaction count share of 0.1. Based on Rao's observation that the maximum distance of two loci forming a loop usually does not exceed 2 Mb, the upper boundary was set to this value. The upper distance settings decrease the search space from 40.5 million to 3.9 million candidates. The count of non-zero interactions gives the 40.5 million candidates. However, the parameter for the maximum distance between the two loci is adjustable. The p-value selection based on continuous negative binomial distributions with level 0.05 reduces the search space from 3.9 million to 530,000 candidates for chromosome 1. Pruning of the candidates with fewer absolute interactions than the maximum interaction count share of 0.1 further decreases the search space to 82,000 candidates. The candidate pooling per neighborhood decreases the search space to only 3515 candidates and, the application of mean background filtering ($mean(background) \geq mean(peak)$), gives a vastly small number to apply the testing with Wilcoxon rank-sum test. A good candidate selection helps to decrease the search space drastically. The Wilcoxon rank-sum test gets only 0.00008% of the original candidates to test starting from 40.5 million candidates.

For other cell lines published by Rao 2014, the situation is comparable (Table 5). For all cell lines, the number of detected candidates is of the same order of magnitude, which indicates a robust candidate selection with the chosen continuous negative binomial distributions. Another essential aspect of reducing the search space is the observation that peaks in Hi-C interaction matrices have a two-dimensional area and not single elements. Peaks are only detectable in the context of their local neighborhood, as the significance given by the continuous negative binomial distributions is not enough. The independent candidate selection per genomic distance leads to multiple candidates per neighborhood, and consequently, only the one with the highest observed/expected value can be considered the peak. The pooling of the candidates under these conditions leads to a reduction of the search space in GM12878 cells of a factor of 23. The reduction rates on the other cell types are similar. However, the situation is different after testing

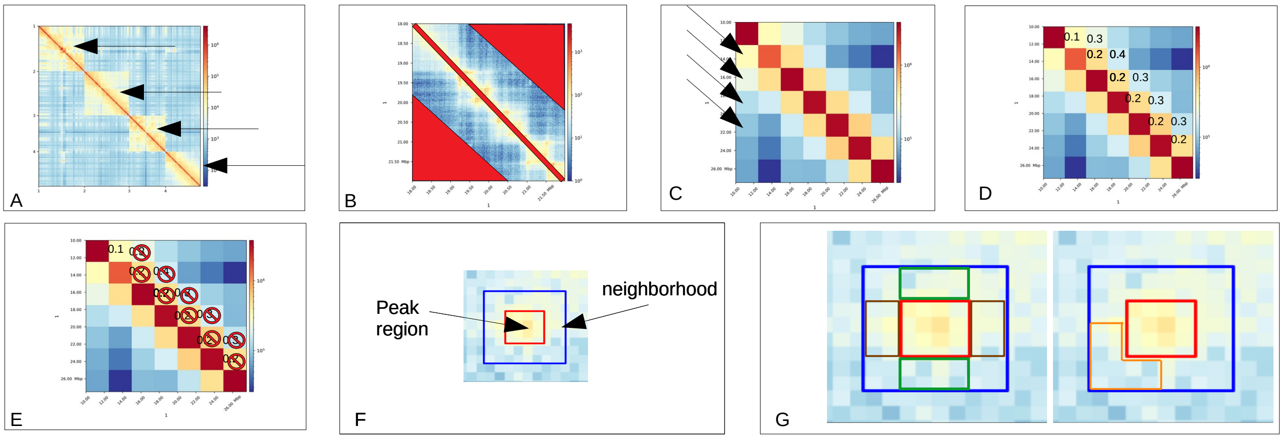

Figure 1. Graphical representation of the loop detection algorithm: A: Compute each chromosome independently. B: Accept an interaction if their relative distance is within: *0 < relative distance < maxLoopSize*. C: (Optional: Remove too low observed vs. expected value before fitting.) Fit cNB distribution per relative distance. D: Compute a p-value for each interaction. E: Reject the candidate if the p-value is too high or the interaction value is too small. F: Define neighborhood around an interaction. Accept as the candidate the one with the highest interaction. G: Apply testing of the peak region vs. vertical, horizontal, and bottom left neighborhood. Reject candidate if: a) maximum or mean of peak region is smaller than the maximum or mean of the neighborhood or b) the p-value computed by Wilcoxon rank-sum test comparing peak and neighborhood region is too high.

Data	HiCExplorer (2 Mb)	HiCExplorer(8 Mb)	HiCCUPS	HiCCUPS (8 Mb)	HOMER	chromosight (2 Mb)	chromosight (8 Mb)	cooltools (2 Mb)	cooltools (8 Mb)
GM12878	9147	10225	12865	10603	7182	50255	60789	8934	9987
HMEC	5679	5705	7539	7424	7152	20159	20160	1698	5259
HUVEC	3466	3489	2199	2119	4052	16607	16621	1698	1869
IMR90	8025	8205	10625	10255	9556	34100	34572	8116	8582
K562	5748	5838	5383	5093	6968	25470	25811	4166	4527
KBM7	4074	4101	2366	1924	3170	21299	21341	1400	1869
NHEK	5166	5211	3198	2937	5409	25724	25770	2334	2662

Table 1. Detected loops on different cell types cells from Rao 2014, with 10kb resolution, HiCExplorer, and HiCCUPS with applied KR correction.

the peak region (Table 1). The number of detected loops differs between 3000 to 10,000 loops. The non-zero values and implicitly the read coverage per bin are considered to explain this different detection behavior; the higher the read coverage, the more regions are detected (see Table 1 and 5). The candidate selection approach via the definition of a neighborhood makes the algorithm sensitive to the Hi-C interaction matrix's resolution. The lower the resolution, the smaller the neighborhood needs to be. Otherwise, the chances of having elements in the neighborhood which are peaks or TADs, or even the main diagonal are too high. Decreasing the size of the neighborhood creates at the same time another issue: the neighborhood and therefore the number of elements in the peak and background regions are becoming too less. This leads to non-significant test results and leads to the insight that the neighborhood size needs to be adjusted to the bin resolution of the Hi-C matrix, and second, a neighborhood should contain at least around 250 - 300 elements to produce useful results.

Comparison to competitors

The number of detected enriched regions of HiCExplorer, HiCCUPS, HOMER, chromosight, and cooltools differs between the samples. The detection rate is on a comparable level (Table 1), except for chromosight. Chromosight detects significantly more loops with a very low p-value; however, as the loops' visualization (Figure 2) indicates, most detect loops are in very noisy regions, and it is questionable what chromosight exactly detects. To investigate the accuracy, the detected loops are correlated with CTCF (Table 2). The detect loops of HiCExplorer are on a comparable level to HiCCUPS and cooltools; on GM12878, HiCExplorer detects a similar amount of loops compared to HiCCUPS (8 Mb: 7298 vs. 7312) but is more specific (8 Mb: 0.71 vs. 0.68), but for example on HMEC HiCExplorer detect fewer loops (8 Mb: 3810 vs. 5350) and is less specific (8 Mb: 0.66 vs. 0.7). Cooltools detect on K562 fewer loops (8 Mb: 4081 vs. 3224) but is more specific (8 Mb: 0.69 vs. 0.71). The other tested cell lines HUVEC, HMEC, and NHEK present similar behavior. The results of Homer and chromosight differ a lot in comparison to HiCExplorer, HiCCUPS, and cooltools. Homer detects more absolute loops (except for GM12878 and KBM7), but it has a low accuracy over all cell lines. Chromosight detects from all testes approaches the most loops and has the highest number of loops correlated to CTCF. HiCExplorer, HiCCUPS, and cooltools can reach similar detection rates if the p-value thresholds are increased; however, the specificity for significantly enriched regions would be removed from the algorithms. It needs to be mentioned that the correlation with CTCF can only indicate the detected loops' quality. First, loop structures representing gene or polycomb-mediated loops do not have CTCF at their anchor points. Second, the used method with ChIP-Seq data is biased and not available in a two-dimensional space. HiChIP data could be used for a better benchmark but was not available.

In comparison to HiCCUPS, HiCExplorer misses the 2% chromatin loops stated in Rao 2014 for genomic distances > 2 Mb, which should include inter-chromosomal enrichments. These inter-chromosomal enrichments are not detectable by HiCExplorer because each chromosome is computed independently. In our testing, also, HiCCUPS was not able to detect non-inter-chromosomal interactions. Recomputed results on GM12878 with HiCCUPS and three resolutions, 5kb, 10kb, and 25kb, 17768 loops were detected, and 4910 have a distance greater than 2 Mb; on 10kb out of 12865 loops, 2968 have a greater distance than 2 Mb. Contrastly, it is not entirely clear on which base Rao 2014 states that only 2% of the loops are in a range greater than 2 Mb. However, if the correlated loops are computed on HiCCUPS data with all loops of distances greater than 2 Mb are removed, 6205 instead of 6354 loops can be correlated with CTCF. These findings support the restriction to a range of 2 Mb. Also, chromosight and cooltools show no significant dif-

Data	HiCExplorer (2 Mb)	HiCExplorer(8 Mb)	HiCCUPS	HiCCUPS (8 Mb)	HOMER	chromosight (2 Mb)	chromosight (8 Mb)	cooltools (2 Mb)	cooltools (8 Mb)
GM12878	7051 (0.77)	7298 (0.71)	7518 (0.58)	7312 (0.68)	1854 (0.25)	14359 (0.28)	16719 (0.27)	6183 (0.69)	6346(0.63)
HMEC	3808 (0.67)	3810 (0.66)	5350 (0.7)	5321 (0.71)	2480 (0.34)	7123 (0.35)	7123 (0.35)	1155 (0.68)	3808 (0.72)
HUVEC	1665 (0.48)	1666 (0.47)	1330 (0.6)	1316 (0.62)	936 (0.23)	4111 (0.24)	4117(0.24)	1092 (0.64)	1105 (0.59)
K562	4044 (0.7)	4081 (0.69)	3864 (0.71)	3791 (0.74)	1945 (0.27)	10079 (0.39)	10219(0.39)	3106(0.74)	3224(0.71)
NHEK	3800 (0.66)	3831 (0.65)	3577 (0.66)	3524 (0.69)	1774 (0.25)	7590 (0.29)	7610 (0.29)	1416 (0.6)	1485(0.55)

Table 2. Number of detected loops on with CTCF match, percentage in brackets.

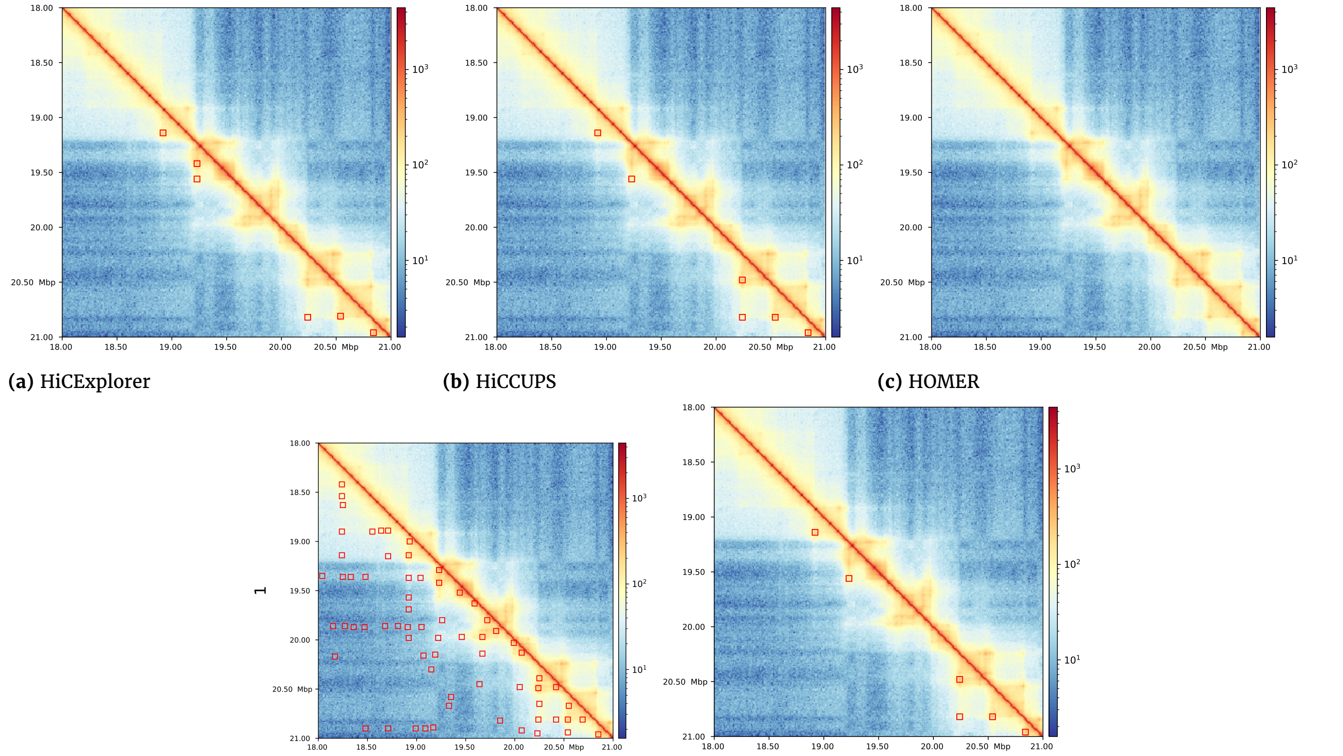

(a) HiCExplorer **(b)** HiCCUPS **(c)** HOMER

(d) chromosight **(e)** cooltools

Figure 2. The plot of chr1 18 - 22 Mb on GM12878 and highlighted the detected loops from each software. HiCExplorer, HiCCUPS, and cooltools show similar results, while Homer is not detecting any loop in the area. Chromosight detects many loops in noisy regions and lacks specificity. Plot with HiCExplorer hicPlotMatrix.

ference in the number of detected and correlated loops between 2 Mb or 8 Mb of distance for the majority of the cells. If the restriction of the genomic distance between two loci is removed for HiCExplorer and all intra-chromosomal contacts are considered, the number of candidates to be tested increases by 10, but the number of accepted peaks increased only minor.

The intersection of detected peaks of HiCExplorer, HiCCUPS, HOMER, chromosight, and cooltools is quite different. HiCExplorer with a search distance of 8 Mb shares ~ 46% of its loops with HiCCUPS genome-wide search and the restricted 8 Mb version on GM12878 cell line; but, e.g., only ~ 24% on HUVEC. HiCExplorer has the highest intersection of detect loops with chromosight, but chromosight also provides the highest number of detect loops. The intersection of detected loops with cooltools is similar to HiCCUPS; the number of intersecting loops with Homer is the lowest. HiCCUPS and cooltools show the highest intersecting numbers, chromosight profits from the high detection rate; while Homer also has with HiCCUPS only a few hundred intersecting loops (Supplementary Table 4). All results show a high correlation rate of the intersected loops of up to 90% with CTCF; indicating if at least two approaches detect a loop, there is a high chance it is a real loop.

The proposed peak detection algorithm was tested on multiple datasets with a 10kb resolution and is the fastest approach of all CPU-based approaches if the 2 Mb search space is considered. Only the HiCCUPS restricted mode on the GPU is slightly faster, for example, 0:56 min to 0:39 on NHEK. On the 8 Mb search distance range, HiCExplorer is also the fastest approach, except for GM12878 cell lines where HiCCUPS in the CPU-based version is faster. HiCExplorer is on GM12878 and 8 Mb search distance ~ 44% faster than chromosight (4:25 min vs. 6:22 min) and uses only 6.7 GB memory while chromosight consumes 39 GB. Moreover, HiCExplorer is two times faster than cooltools if only the loop detection is considered; if the necessary computation of expected values is added, it is almost 3.5 times faster (Supplementary Table 7). Chromosight is the fastest algorithm if only the divorced from the real world single-core performance is measured (Supplementary Table 8). Modern CPUs support up to 64 cores / 128 threads, and data analysis software should use the offered resources as well as possible. For this reason, HiCExplorers' hicDetectLoop does support the parallelization by not only the chromosomes but also an intra-chromosomal parallelization. Homer is, in all scenarios, the slowest algorithm and consumes the most memory. It has the side effect that, for example, the GM12878 dataset could only be computed using one single-core because the memory consumption was already around 100 GB. The chosen approach by the developers of Homer to not support any binary file format to store and access the Hi-C interaction matrix-like Juicer's *hic* or the from all other investigated tools supported *cooler* file format [10], results in a computation based on text files and raw data, and a very poor runtime and memory performance.

Data	HiCExplorer ∩ HiCCUPS (GPU)	HiCExplorer ∩ HiCCUPS (8 Mb)	HiCExplorer ∩ HOMER	HiCExplorer ∩ chromosight	HiCExplorer ∩ cooltools
GM12878	4699	4665	495	6013	4190
HMEC	2724	2722	591	3012	488
HUVEC	862	861	205	1515	683
K562	2265	2262	361	3364	1949
KBM7	566	561	146	1777	469
IMR90	4164	4161	758	4693	3603
NHEK	1359	1340	459	2748	1137

Table 3. intersection of HiCExplorer with 2 Mb search distance with HiCUPS (GPU, full genome), HiCCUPS 8 Mb search distance, Homer (full genome), chromosight 2 Mb search distance, and cooltools 2 Mb search distance.

Data	HiCExplorer ∩ HiCCUPS (GPU)	HiCExplorer ∩ HiCCUPS (8 Mb)	HiCExplorer ∩ HOMER	HiCExplorer ∩ chromosight	HiCExplorer ∩ cooltools
GM12878	4141 (0.88)	4125 (0.88)	408 (0.82)	4953 (0.82)	3687 (0.88)
HMEC	2254 (0.83)	2251 (0.83)	465 (0.79)	2251 (0.75)	438 (0.90)
HUVEC	657 (0.76)	656 (0.76)	117 (0.57)	937 (0.62)	509 (0.75)
K562	1975 (0.87)	1974 (0.87)	275 (0.76)	2642 (0.79)	1684 (0.86)
NHEK	1019 (0.75)	1007 (0.75)	275 (0.60)	1762 (0.64)	865 (0.76)

Table 4. CTCF correlation with intersected loops for 2 Mb search distance, percentage in relation to number of intersected loops, see Table 3

Data	Initial candidates	Candidates for peak detection
GM12878	61.8 mio	1722
K562	19.2 mio	2948
KBM7	14.2 mio	2321
IMR90	19.3 mio	2948
NHEK	10.1 mio	2384
HUVEC	7.6 mio	3249

Table 5. Initial possible candidates vs. reduced candidate set of HiCExplorer for chromosome 1.

Data	Non-zero elements	Sparsity
GM12878	1,810 mio	0.0189
K562	781 mio	0.0081
KBM7	465 mio	0.0048
IMR90	415 mio	0.0043
NHEK	348 mio	0.0036
HUVEC	268 mio	0.0028
HMEC	188 mio	0.0019

Table 6. Sparsity level of the 10 kb Hi-C interaction matrices. The dense matrix contains 309,581 x 309,581 elements.

Discussion

The search space of an algorithm is the dominating factor for its accuracy and performance. Therefore, pruning it should be the primary goal of newly designed algorithms. Bruteforce solutions like HiCCUPS with no restrictions to the search space are, in theory, able to detect all possible enriched regions, but at the cost of hardware demanding implementation. HiCCUPS solved this by the massively parallel computational resources via GPGPU. The limitation of the search space to a genomic distance of 2 Mb has only a small impact on the detected peaks. HOMER, however, has no limitations on the search space, detects less number of loops, and the detected ones have a significantly lower correlation over all samples to CTCF. Moreover, HOMER does support a parallelization per chromosome like HiCExplorer but is significantly slower than all other solutions and uses more memory per core extensively. Homer's poor runtime performance can be explained by computing on raw data, while all other approaches use precomputed interaction matrices. Chromosight is a fast detection approach and provides the fastest single-core performance; however, it lacks specificity and detects many loops that should be considered noise, even if these loops are provided with a high significance. Cooltools, with its reimplementation of the HiCCUPS approach, is fast and more flexible by providing a genome distance search. The results are good, but it raises questions why they are not more similar to Juicer's HiCCUPS results if both use the same algorithm. Furthermore, it could be shown that the sparsity and therefore read coverage of a Hi-C interaction matrix has a significant influence on the detection of peaks in their neighborhood. The sparser a Hi-C interaction matrix is, the more likely it is that possible valid region detected by the continuous negative binomial distribution filtering are rejected by Wilcoxon rank-sum test. The high amount of different detect loops and the correlation rates to CTCF can be explained in multiple ways. First, the correlation to CTCF is caused by biology itself. Not all loops have CTCF as a binding protein at its anchors; gene-loops or polycomb-mediated loops lack it. Moreover, the used CTCF data from ENCODE[4] is already ten years old, but no newer data was available. The method to detect the correlation is suboptimal: ChIP-Seq data is one-dimensional, and a loop has two-dimensional coordinates. If a CTCF peak is detected at both coordinates, it does not have to imply CTCF is present as a loop binding protein. The usage of HiChIP data would be of a higher biological meaning; however, it was not available for the used Hi-C cell lines. Second, the Hi-C data is created with in-situ Hi-C and has a higher noise level than newer approaches like Arima Hi-C. Detections of loops in noisy areas cause the competing algorithms' low intersection values, primarily chromosight detects more noise than loops. The solution for this is simple: Better Hi-C with less noise and more HiChIP data needs to be available to compare loop detection algorithms better.

Availability of source code and requirements

HiCExplorer is licensed under GPLv3 and is available on Github (https://github.com/deeptools/HiCExplorer/) or as a conda package in the bioconda channel [22]. HiCExplorer is implemented in Python 3.6, 3.7. and 3.8 for Linux and macOS.

Availability of supporting data and materials

Hi-C data: GSE63525; Rao et al. [8]. CTCF for: Gm12878 from GSM935611; Hmec from GSM749753; Huvec from GSM749749; K562 from GSM733719 and Nhek from GSM733636.

Declarations

4 https://www.encodeproject.org/

Competing Interests

The author(s) declare that they have no competing interests.

Funding

German Federal Ministry of Education and Research [031 A538A de.NBI-RBC awarded to R.B.]; German Federal Ministry of Education and Research [031 L0101C de.NBI-epi awarded to B.G.]. R.B. was supported by the German Research Foundation (DFG) under Germany's Excellence Strategy (CIBSS - EXC-2189 - Project ID 390939984).

Author's Contributions

JW: Designed and implemented the presented algorithm, and wrote the manuscript. RB: contributed to the manuscript. BG: contributed to the manuscript.

Acknowledgements

We thank Simon Bray and Anup Kumar for proofreading the manuscript.

References

1. Dekker J, Rippe K, Dekker M, Kleckner N. Capturing chromosome conformation. science 2002;295(5558):1306–1311. [PubMed:11847345] [doi:10.1126/science.1067799].
2. Simonis M, Klous P, Splinter E, Moshkin Y, Willemsen R, De Wit E, et al. Nuclear organization of active and inactive chromatin domains uncovered by chromosome conformation capture-on-chip (4C). Nature genetics 2006;38(11):1348. [PubMed:17033623] [doi:10.1038/ng1896].
3. Zhao Z, Tavoosidana G, Sjölinder M, Göndör A, Mariano P, Wang S, et al. Circular chromosome conformation capture (4C) uncovers extensive networks of epigenetically regulated intra-and interchromosomal interactions. Nature genetics 2006;38(11):1341. [PubMed:17033624] [doi:10.1038/ng1891].
4. Dostie J, Richmond TA, Arnaout RA, Selzer RR, Lee WL, Honan TA, et al. Chromosome Conformation Capture Carbon Copy (5C): a massively parallel solution for mapping interactions between genomic elements. Genome research 2006;16(10):1299–1309. [PubMed:16954542] [PubMed Central:PMC1581439] [doi:10.1101/gr.5571506].
5. Lieberman-Aiden E, Van Berkum NL, Williams L, Imakaev M, Ragoczy T, Telling A, et al. Comprehensive mapping of long-range interactions reveals folding principles of the human genome. Science 2009 oct;326(5950):289–293. http://www.ncbi.nlm.nih.gov/pubmed/19815776http://www.pubmedcentral.nih.gov/articlerender.fcgi?artid=PMC2858594, [PubMed:19815776] [PubMed Central:PMC2858594] [doi:10.1126/science.1181369].
6. Bonev B, Cavalli G. Organization and function of the 3D genome. Nature Reviews Genetics 2016;17(11):661. [PubMed:28704353] [doi:10.1038/nrg.2016.147].
7. Tan-Wong SM, Zaugg JB, Camblong J, Xu Z, Zhang DW, Mischo HE, et al. Gene loops enhance transcriptional directionality. Science 2012;338(6107):671–675.
8. Rao SSP, Huntley MH, Durand NC, Stamenova EK. A 3D Map of the Human Genome at Kilobase Resolution Reveals Principles of Chromatin Looping. Cell 2014;159(7):1665–1680. http://dx.doi.org/10.1016/j.cell.2014.11.021, [PubMed:25497547] [PubMed Central:PMC5635824] [doi:10.1016/j.cell.2014.11.021].
9. Heinz S, Benner C, Spann N, Bertolino E, Lin YC, Laslo P, et al. Simple Combinations of Lineage-Determining Transcription Factors Prime cis-Regulatory Elements Required for Macrophage and B Cell Identities. Molecular Cell 2010 may;38(4):576–589. https://www.sciencedirect.com/science/article/pii/S1097276510003667?via{%}3Dihub, [PubMed:20513432] [PubMed Central:PMC2898526] [doi:10.1016/j.molcel.2010.05.004].
10. Abdennur N, Mirny LA. Cooler: scalable storage for Hi-C data and other genomically labeled arrays. Bioinformatics 2020;36(1):311–316.
11. Matthey-Doret C, Baudry L, Breuer A, Montagne R, Guiglielmoni N, Scolari V, et al. Computer vision for pattern detection in chromosome contact maps. Nature communications 2020;11(1):1–11.
12. Mifsud B, Martincorena I, Darbo E, Sugar R, Schoenfelder S, Fraser P, et al. GOTHiC, a probabilistic model to resolve complex biases and to identify real interactions in Hi-C data. PloS one 2017;12(4).
13. Cao Y, Chen Z, Chen X, Ai D, Chen G, McDermott J, et al. Accurate loop calling for 3D genomic data with cLoops. Bioinformatics 2020;36(3):666–675.
14. Xu Z, Zhang G, Wu C, Li Y, Hu M. FastHiC: a fast and accurate algorithm to detect long-range chromosomal interactions from Hi-C data. Bioinformatics 2016;32(17):2692–2695.
15. Xu Z, Zhang G, Jin F, Chen M, Furey TS, Sullivan PF, et al. A hidden Markov random field-based Bayesian method for the detection of long-range chromosomal interactions in Hi-C data. Bioinformatics 2016;32(5):650–656.
16. Imakaev M, Fudenberg G, McCord RP, Naumova N, Goloborodko A, Lajoie BR, et al. Iterative correction of Hi-C data reveals hallmarks of chromosome organization. Nature Methods 2012 sep;9(10):999–1003. http://www.nature.com/doifinder/10.1038/nmeth.2148, [PubMed:22941365] [PubMed Central:PMC3816492] [doi:10.1038/nmeth.2148].
17. Knight PA, Ruiz D. A fast algorithm for matrix balancing. IMA Journal of Numerical Analysis 2013;33(3):1029–1047. [doi:10.1093/imanum/drs019].
18. Love MI, Huber W, Anders S. Moderated estimation of fold change and dispersion for RNA-seq data with DESeq2. Genome biology 2014;15(12):1–21.
19. Robinson MD, McCarthy DJ, Smyth GK. edgeR: a Bioconductor package for differential expression analysis of digital gene expression data. Bioinformatics 2010;26(1):139–140.
20. McCarthy DJ, Chen Y, Smyth GK. Differential expression analysis of multifactor RNA-Seq experiments with respect to biological variation. Nucleic acids research 2012;40(10):4288–4297.
21. Andrey G, Schöpflin R, Jerković I, Heinrich V, Ibrahim DM, Paliou C, et al. Characterization of hundreds of regulatory landscapes in developing limbs reveals two regimes of chromatin folding. Genome research 2017;27(2):223–233. [PubMed:27923844] [PubMed Central:PMC5287228] [doi:10.1101/gr.213066.116].
22. Grüning B, Dale R, Sjödin A, Chapman BA, Rowe J, Tomkins-Tinch CH, et al. Bioconda: sustainable and comprehensive software distribution for the life sciences. Nature methods 2018;15(7):475. [PubMed:29967506] [doi:10.1038/s41592-018-0046-7].

Click here to access/download
Supplementary Material
Loop_detection_supplementary.pdf
