# Supplementary material for: Loop detection using Hi-C data with HiCExplorer
Source: Gigascience. 2022 Jul 9;11:giac061. doi: 10.1093/gigascience/giac061 (PMC9270730; doi:10.1093/gigascience/giac061)
Supplement: giac061_GIGA-D-21-00069_Revision_1 [file giac061_giga-d-21-00069_revision_1.pdf]

GigaScience
Loop detection using Hi-C data with HiCExplorer
--Manuscript Draft--

**Manuscript Number:**	GIGA-D-21-00069R1
**Full Title:**	Loop detection using Hi-C data with HiCExplorer
**Article Type:**	Technical Note
**Funding Information:**	Bundesministerium für Bildung, Wissenschaft, Forschung und Technologie (031 A538A de.NBI-RBC) — Prof. Dr. Rolf Backofen<br>Bundesministerium für Bildung, Wissenschaft, Forschung und Technologie (031 L0101C de.NBI-epi) — Dr. Björn Grüning<br>Deutsche Forschungsgemeinschaft (CIBSS - EXC-2189 - Project ID 390939984) — Prof. Dr. Rolf Backofen
**Abstract:**	Background: Chromatin loops are an essential factor in the structural organization of the genome. The detection of chromatin loops in Hi-C interaction matrices is a challenging and compute-intensive task. The presented approach shows a chromatin loop detection algorithm that applies a strict candidate selection based on continuous negative binomial distributions and performs a Wilcoxon rank-sum test to detect enriched Hi-C interactions.<br>Results: HiCExplorer's loop detection has a high detection rate and accuracy. It is the fastest available CPU implementation and utilizes all threads offered by modern multi-core platforms.<br>Conclusions: HiCExplorers method to detect loops by using a continuous negative binomial function combined with the donut approach from HiCCUPS leads to reliable and fast computation of loops. All investigated loop-calling algorithms provide a differing number of detect loops and intersect in the best cases by 50%. The tested in-situ Hi-C data contains a large amount of noise; more similar results in loop calling requires cleaner Hi-C data and, therefore, improvements in the data creation.
**Corresponding Author:**	Joachim Wolff<br>Albert-Ludwigs-Universität Freiburg: Albert-Ludwigs-Universitat Freiburg<br>Freiburg im Breisgau, Baden-Württemberg GERMANY
**Corresponding Author Secondary Information:**	
**Corresponding Author's Institution:**	Albert-Ludwigs-Universität Freiburg: Albert-Ludwigs-Universitat Freiburg
**Corresponding Author's Secondary Institution:**	
**First Author:**	Joachim Wolff
**First Author Secondary Information:**	
**Order of Authors:**	Joachim Wolff<br>Rolf Backofen, Dr.<br>Björn Grüning, Dr.
**Order of Authors Secondary Information:**	
**Response to Reviewers:**	Respond to the reviewers comments for ‘Loop detection using Hi-C data with HiCExplorer’<br><br>General remark from the authors<br><br>We thank the reviewers for their comments and suggestions that helped us to improve the presented manuscript. We rewrote parts of the manuscript to make the scope of

the publication and the used techniques more clear.

Detailed reply to the reviewers comments

Reviewer #1: Wolff et al. present the python version of HiCExplorer for loop detection. The algorithm is included in the Galaxy HiCExplorer webserver (Wolff et al. 2020), although the publication about the webserver did not describe the algorithm in detail. HiCExplorer uses the same donut approach as HiCCUPS (Rao et al. 2014) with a few notable differences. HiCExplorer selects candidate peaks based on the significance of the distance-corrected observed/expected ratio using a negative binomial model, and compares the peak's enrichment to its neighbourhood's using a Wilcoxon rank-sum test.

The method is appropriate for chromatin loop identification and it performs similarly to existing methods both in terms of computational requirements and specificity of the detected loops. However, the manuscript in its current format does not describe the method adequately, and the comparison with the other methods is limited and inconsistent. It would be good to describe each step of the
method (filtering based on distance, candidate selection based on negative binomial test, additional filtering options, local enrichment testing using different neighbourhoods in a Wilcoxon rank-sum test).

Reviewer #1:
The graphical representation currently included for the algorithm is not informative for most of these steps.
Answer:
We changed the graphic to help the reader to better understand the graphic. However, the graphical description is only complete under consideration of the caption and friendly ask reviewer #1 to please consider this in their reasoning.

Reviewer #1:
For the scientific community, it would be more informative if this method's performance would be further analyzed. Even though it is mentioned that the loop detection greatly depends on the initial parameters, the results do not show how the parameters influence it.
Answer:
We showed the impact of the parameters in the section ‘HiCExplorer candidate selection’ and extended it.

Reviewer #1:
The comparison of HiCExplorer with other existing methods is inconsistent. Finally, the text would need heavy editing for language, clarity and minor spelling mistakes.

Answer:
Reviewer #1 lacks explaining to us why in their view the comparison to other methods is inconsistent. If such a claim is made, and no specific comment for this is listed in the ‘specific comments’ section, it is very hard for us to improve the paper at this point. We request clarification from reviewer #1 about this comment.

Reviewer #1:
Specific comments:
The background does not clearly lay out the motivation behind designing this algorithm. There are similar existing methods that are fast. Why is it expected to detect chromatin loops better?

Answer:

Powered by Editorial Manager® and ProduXion Manager® from Aries Systems Corporation

HiCCUPS (and its reimplementation in cooltools) uses the Poisson distribution which is a discrete distribution. Hi-C data is discrete data, but the commonly preferred way to analyze Hi-C data is not to work on raw, but on corrected data. The correction of the data with e.g. ICE or KR transforms the discrete data to continuous, making the Poisson distribution not usable. HiCCUPS solves this by reverting the correction and by operating on the raw data. We think this is not appropriated and it is better to use a distribution that can handle the Hi-C data independent of the case it is discrete or continuous which we presented with the continuous negative binomial distribution. It behaves like the binomial distribution in case the Hi-C data is uncorrected and discrete.

The factorial factor of a Poisson distribution could have been also been replaced by a gamma function to make it continuous, however, count data tends to overdispersion. The Poisson distribution assumes the variance and expected value are equal, the Negative Binomial distribution allows different values. We added a figure in the supplements to show we have overdispersion in the raw data and therefore the continuous negative binomial distribution is the theoretically better choice.

Besides the mathematical arguments, the original goal to design this algorithm was that at the time the development started (late spring 2018), no tool supporting the detection of loop data with the from us preferred 'cooler' file format was existing, the well-known HiCCUPS algorithm was available only as a GPU version and searched the full genome; while in the study from the authors (Rao 2014) they wrote that 98% of the loops are in a 2 MB distance range. However, the algorithm was added to HiCExplorer in spring 2019, but HiCCUPS added an experimental CPU support (which is experimental until today) and other tools supporting the cooler format (cooltools, chromosight) were published too. Today, the loop detection of HiCExplore is 'yet another one'; however, we think for completeness and citation reasons, the details of this algorithm should be published in a peer-reviewed journal.
Moreover, the presented algorithm is faster than cooltools e.g. Gm12878 and 2 MB distance (1:11 min vs 12:13 min) and requires less memory (2.7 GB vs 20 GB); faster than chromosight (1:11 min vs 3:23) and requires significantly less memory (2.7 GB vs 38.6 GB). HiCCUPS using the CPU version and the 8 MB restriction is faster on GM12878 (4:25 vs 2:41) but needs more memory (6.7 GB vs 27.7 GB). On all other datasets, HiCExplorer restricted to 8 Mb is faster and requires less memory.
Concerning the accuracy: Chromosight detects too many loops and is too unspecific, hiccups and cooltools have similar results to HiCExplorer.
HiCCUPS in the GPU version is the fastest one, however, it also needs significantly more memory. The loop detection of HiCExplorer can run on any notebook, while all other tools need high memory requirements which are not available in regular notebooks or desktop computers or need an Nvidia GPU supporting CUDA. Also, GPU access is not that common, and also sometimes difficult to get in cluster environments. Last, HiCCUPS is only available with the '.hic' file format, and export of cooler to hic is not possible. I asked the authors of Juicer if they provide anything, but I got no answer (https://groups.google.com/g/3d-genomics/c/jCSQk4oEl5w/m/gqyJD0FOBwAJ). To rebuild the Hi-C matrices from scratch just for Juicer's HiCCUPS algorithm (and similar for Homer text file based solution) is not only time consuming but can add potential errors if different Hi-C matrix build tools classify some reads in another way (e.g. if it is a dangling end or similar Hi-C errors).

Reviewer #1:
This is not a 3D genomics specialized journal, therefore the text should introduce Hi-C and its challenges clearly. For example, the notion that genome properties and ligations affect Hi-C data analysis is mentioned in the methods section without further elaboration. It would be hard for readers to understand why authors are normalizing for ligation events in their algorithm.
Answer:
We specified this better. We want to accentuate that the different expected value computation methods are offered, and it is the choice of the user the select one which they think fits best to the data. Our investigation shows the mean is the best way to compute it, however, we do not arrogate what is the best decision for an individual dataset and offer therefore all three methods.

Reviewer #1:
The background introduces a few methods that are not aimed at detecting chromatin

	loops (e.g. GOTHiC) or not designed for Hi-C (e.g. cLoops) and are also not used in the comparison. It would be more useful to describe the algorithms of those methods that are comparable to Hi-C explorer in terms of their goal and design.<br>Answer:<br>Reviewer #1 states one comment above this one, that this journal is not a 3D genomic specialized one and therefore more explanation should be added. We think the way we describe additional algorithms which are for the inexperienced reader very similar to what we present, is necessary for the reader to understand why these algorithms are not considered. Moreover, we describe algorithms the algorithms which are part of the comparison.<br><br>Reviewer #1:<br>Figure 1, which represents the steps of the algorithm, does not make it clear what happens at each step, some of arrows seem to point to random pixels, e.g. in panel C.<br>Answer:<br>The arrows in figure 1C do not point to random pixels, they point to the genomic distances, and these are given in the matrix by the diagonals. The caption describes it:'Fit cNB distribution per relative distance.' If reviewer #1 thinks the graphic irritates more than it helps, we can remove it and rely only on the textual description of the algorithm instead of a graphical one. However, the graphical description of the algorithm is only complete under the consideration of the caption, which reviewer #1 obviously did not consider.<br><br>Reviewer #1:<br>More elaboration on the use of the three different expected value calculation methods would be needed. Which one is more appropriate for a mammalian vs. an insect Hi-C does it depend on the genome size, the sequencing depth or the sparsity of the data?<br>Answer:<br>We extended the section 'HiCExplorer candidate selection' to address this comment.<br><br><br>Reviewer #1:<br>The negative binomial distribution does model well the read counts in most high-throughput sequencing experiments, but the rationale given for choosing it is not appropriate.<br>Answer:<br>We added an additional explanation and an overdispersion test with the Poisson distribution to show why we use it. However, reviewer #1 writes the whole time in their critics of a 'negative binomial' distribution. This is wrong and is one major aspect of our proposed algorithm. We use a continuous negative binomial distribution and not a discrete negative binomial distribution. This was explained in the manuscript and implicates, in combination with Hi-C correction like ICE or KR, already the rationale of choosing it. We encourage reviewer #1 to consider this major difference especially in comparison to HiCCUPS and their ignoring of the correction factors.<br><br>Reviewer #1:<br>Also, citing a stackexchange discussion for the methods is not suitable.<br>The numbers in most tables could be better appreciated if they were represented in a figure.<br>Answer:<br><br>We are aware that a citation of stackexchange is not optimal; we contacted the author of the post, Prof. Gordon Smyth from WEHI and he wrote that we should cite:<br><br>McCarthy, DJ, Chen, Y, Smyth, GK (2012). Differential expression analysis of multifactor RNA-Seq experiments with respect to biological variation. Nucleic Acids Research 40, 4288-4297.<br><br>We had the citation already in the manuscript and removed the reference to stackexchange. We do not want to claim the replacement of the binomial coefficient with the gamma function is our work, therefore we leave it to the editor to decide if we shall cite it or if the solution as proposed by Prof. Smyth is the appropriate way.

Powered by Editorial Manager® and ProduXion Manager® from Aries Systems Corporation

Reviewer #1:
What was the reason to increase the distance only to 8Mb instead of using the full genome as comparison, especially given that some of the compared methods only work on the full genome?
Answer:

The authors of HiCCUPS write in their own study (Rao 2014) that 98% of all loops are detected within the range of 2 MB. However, their GPU tool ignored this for quite some time and computed it on the full genome. With a newer version, an experimental labeled CPU version with the 8 MB restriction was published and the GPU version got the restriction as an option too. HiCCUPS does not allow to use any other distance than these 8 Mb, and for this reason, we compared the results to 8 Mb. Our tool is able to compute the loops within every distance the user is defining it.
Moreover, we stated already in the text why we do not investigate the full chromosomes in detail:

'If the restriction of the genomic distance between two loci is removed for HiCExplorer and all intra-chromosomal contacts are considered, the number of candidates to be tested increases by a factor of 10, but the number of accepted peaks increased only minor.'

Reviewer #1:
The bottom left neighbourhood in HiCCUPS is assessed, because they only use the upper triangle in the Hi-C matrix, and the bottom left neighbourhood represents the shorter interactions. In Figure 2, the detected interactions are indicated on the bottom triangle , which is counterintuitive.
Answer:
We do not think that computational details (upper triangle in the computation) and the visualization of data in the lower triangle confuse the readers. However, visualization in the bottom triangle is quite common, for example:
HiCCUPS, Rao 2014, Figure 3 https://www.cell.com/fulltext/S0092-8674(14)01497-4
Chromosight , Matthey-Doret 2020, Figure 2 https://www.nature.com/articles/s41467-020-19562-7

Reviewer #1:
Fig 2A is showing the same data as Fig 2A in the Galaxy HiCExplorer publication (Wolff et al 2020), but the detected loops indicated are different. What is the reason for that?
Answer:
The algorithm used in the Galaxy HiCExplorer 3 publication was based on HiCExplorer 3.2; with HiCExplorer 3.5 we changed the loop detection algorithm to its current form. For this reason, the detect loops differ. We changed the algorithm because we were not happy with the performance in terms of accuracy of the detect loops and also on the utilization of the threading of modern CPUs. For comparison of the algorithmic differences, please compare the manuscript to the bioRxiv publication of the loop detection.

Reviewer #1:
The difference between the proportion of CTCF-bound loops for the different methods is probably not significant. It should be tested.

Answer:

We have stated the proportion of the CTCF for each of the different methods. We think this was not recognized by reviewer #1 or we hereby ask for clarification of their comment.

Reviewer #2: This paper provided a loop detection method using continuous negative

binomial function combined with donut approach. To test the performance of this method, the authors used in-situ Hi-C data by Rao 2014 in GM12878, K562, IMR90, HUVEC, KBM7, NHEK and HMEC cell lines. This method showed comparable results with HiCCUPS and cooltools and better outputs than HOMER and chromosight. The significant advantage is the utilization of modern computational resources. The following are my comments:

Reviewer #2:
1. The author claimed the advantages in utilizing computational resources. The authors need to clarify how their algorithm contributes to this advantage.

Answer:
We did this in the 'comparison to competitors section':

"For this reason, HiCExplorers' hicDetectLoop does support the parallelization by not only the chromosomes but also an intra-chromosomal parallelization."

We extended the explanation a bit. Also, the lower memory usage is clearly described in the text and in the tables.

Reviewer #2:
2. It will be helpful for the users to know the performance of the software at various sequencing depths, which can be achieved by down-sampling the high resolution datasets.
Answer:

We compare in the submitted manuscript different cell types which have all different sequencing depths for exactly this reason and this was already discussed in the section 'HiCExplorer candidate selection', starting at "For other cell lines published by Rao 2014"; please also consider Supplementary Table 7 where the different read coverages are listed.

Reviewer #2:
3. The authors need to compare (or at least discuss) Fit-Hi-C and Peakchachu. A table showing the strength and limitation of each method will be helpful.
Answer:
We think reviewer #2 means the tool 'Peakachu' (https://github.com/tariks/peakachu) and not 'Peakchachu'. The last one did not show any results on Google.

Fit-Hi-C is a method to detect significant Hi-C contacts. In our understanding this is very similar to GOTHiC. However, we gave it a try and on a Gm12878 10kb dataset, it detected significant contacts in the 100,000-ends. With an additional filter step loops can be detected. However, these detected loops have a low correlation to CTCF, overall the tool is not good in detecting enriched regions.

Peakachu: There is a large difference in the results that the authors present in their publication (https://www.nature.com/articles/s41467-020-17239-9) and the results we have, especially the comparison to HiCCUPS. One explanation is the provided trained model of Peakachu. We think the results of the detection rate and the correlation of the loop with orthogonal data show the provided model does not generalize well. Even more, a reason could be the approach the authors of Peakachu used in their publication: Instead of letting Fit-Hi-C and HiCCUPS compute their loops and compare detected loop locations with each other, they took all 'candidate' locations of the two algorithms and used their own 'merging' algorithm to filter out the loop locations. We think this is methodically critical, and are irritated why this has been accepted by the reviewers. Instead of a full comparison of the algorithms, only the first detection was from Fit-Hi-C and HiCCUPS, but the second one was from the authors of Peakachu. (Section 'Methods' of the Peakachu publication, 'Loop detection with HiCCUPS and Fit-HiC': 'To make a fair comparison with Peakachu and HiCCUPS on the 100% matrix, we sorted the detected interactions by p-values and performed the same pooling algorithm used by Peakachu')

Based on the performance of Fit-HiC and Peakachu on the Gm12878 dataset we decided to not test them on the other cell types in detail.

Powered by Editorial Manager® and ProduXion Manager® from Aries Systems Corporation

	Reviewer #2:<br>To be honest, I don't think any method is clearly better than the other. They are just different approaches.<br>Answer: We agree with this viewpoint on the algorithms and therefore we present our manuscript in GigaScience as a Technical Note. To quote the criteria of the journal:<br><br>“The tool or method needs to have been tested, and properly compared to any existing tools or methods used by the community. It does not necessarily have to outperform existing approaches, but it should show innovation in the approach, implementation, or have added benefits that have been needed in this arena.”<br>https://academic.oup.com/gigascience/pages/technical_note<br><br>We think to fulfill these requirements. HiCCUPS and cooltools ignore the correction factors and uses a discrete distribution assumption and ignore the overdispersion in the data. By choosing a more appropriate model that is also able to work with continuous data, the continuous negative binomial distribution provides a mathematical better model for Hi-C loop detection. Moreover, we provide a CPU-based software enabling many researchers without access to Nvidia GPUs to perform our software. Also, HiCExplorer does not require training with additional and orthogonal data like ‘Peakachu’ which might be not available. We could also show how strong the dependency on a good trained model for Peakachu is. HiCExplorer outperforms Homer, Fit Hi-C and Peakachu by a large amount, time, memory, and accuracy wise.<br><br><br>Reviewer #2:<br>4. It is better to use other types of orthogonal data like HiChIP, ChIA-PET to evaluate the loops called by these methods. There are H3K27ac HiChIP, SMC1 HiChIP, CTCF ChIA-PET and RAD21 ChIA-PET data in GM12878.<br>Answer:<br>We added another table to the Supplementary Material comparing to the listed data for Gm12878 only. The accuracy scores confirm our claims of the CTCF ChIP-Seq data of the submitted manuscript.<br><br>Reviewer #2:<br>5. Just a minor suggestion. There are a lot of tables in the manuscript, which makes it hard for the readers to compare. It might be better to use figures instead.<br><br>Answer:<br>We added a few graphics to replace some tables. Other tables have been moved to the Supplementary Material.
**Additional Information:**	
**Question**	**Response**
Are you submitting this manuscript to a special series or article collection?	No
**Experimental design and statistics**<br><br><br>Full details of the experimental design and statistical methods used should be given in the Methods section, as detailed in our Minimum Standards Reporting Checklist. Information essential to interpreting the data presented should be made available in the figure legends.<br><br><br>Have you included all the information	Yes

requested in your manuscript?	
**Resources**<br><br>A description of all resources used, including antibodies, cell lines, animals and software tools, with enough information to allow them to be uniquely identified, should be included in the Methods section. Authors are strongly encouraged to cite Research Resource Identifiers (RRIDs) for antibodies, model organisms and tools, where possible.<br><br>Have you included the information requested as detailed in our Minimum Standards Reporting Checklist?	Yes
**Availability of data and materials**<br><br>All datasets and code on which the conclusions of the paper rely must be either included in your submission or deposited in publicly available repositories (where available and ethically appropriate), referencing such data using a unique identifier in the references and in the "Availability of Data and Materials" section of your manuscript.<br><br>Have you have met the above requirement as detailed in our Minimum Standards Reporting Checklist?	Yes

Manuscript Click here to access/download;Manuscript;manuscript.pdf

GigaScience, XXXX, 1–8

doi: xx.xxxx/xxxx
Manuscript in Preparation
Technical Note

TECHNICAL NOTE

Loop detection using Hi-C data with HiCExplorer

Joachim Wolff[1,*], Rolf Backofen[1,2] and Björn Grüning[1]

[1]Bioinformatics Group, Department of Computer Science, University of Freiburg, Georges-Köhler-Allee 106, 79110 Freiburg, Germany and [2]Signalling Research Centres CIBSS, University of Freiburg, Schänzlestr. 18, 79104 Freiburg, Germany

*wolffj@informatik.uni-freiburg.de

Abstract

Background: Chromatin loops are an essential factor in the structural organization of the genome. The detection of chromatin loops in Hi-C interaction matrices is a challenging and compute-intensive task. The presented approach shows a chromatin loop detection algorithm that applies a strict candidate selection based on continuous negative binomial distributions and performs a Wilcoxon rank-sum test to detect enriched Hi-C interactions. **Results:** HiCExplorer's loop detection has a high detection rate and accuracy. It is the fastest available CPU implementation and utilizes all threads offered by modern multi-core platforms. **Conclusions:** HiCExplorers method to detect loops by using a continuous negative binomial function combined with the donut approach from HiCCUPS leads to reliable and fast computation of loops. All investigated loop-calling algorithms provide a differing number of detect loops and intersect in the best cases by ~ 50%. The tested in-situ Hi-C data contains a large amount of noise; more similar results in loop calling requires cleaner Hi-C data and, therefore, improvements in the data creation.

Key words: Hi-C, Hi-C loop detection, DNA loops

Introduction

Chromosome conformation capture (3C) [1] and its successors 4C [2, 3], 5C [4] and Hi-C [5] are protocols to study the three dimensional structure of a genome. With Hi-C data, a genome-wide interaction map of the chromatin can be created, and chromatin loops can be inferred. Chromatin loops reflect the interaction of promoters and enhancers, gene loops, architectural loops, or polycomb-mediated regions [6] and can be detected as enriched regions in comparison to their neighborhood. By identifying these regions, it can be shown that there are long-range regulations that impact, e.g., the directionality of RNA synthesis [7], or the long-distance between cis-regulatory elements [6] that can not be explained and shown otherwise. Based on Rao [8], the genomic distance between two loci is usually limited to ~ 2 megabases (Mb).
There are many algorithms that can detect loops: HiCCUPS uses a *donut algorithm*, which considers all elements of a Hi-C interaction matrix as peaks and tests if the region around them is significantly different from the neighboring interactions. HiCCUPS is part of the software Juicer[1], and the implementation requires a general-purpose GPU (GPGPU), which imposes a barrier for users not having access to Nvidia GPUs. However, an experimental CPU-based implementation was released too. The balancing of Hi-C matrices with algorithms like ICE [9] or KR [10] are widely used in Hi-C data analysis, but the loop detection algorithm of HiCCUPS breaks with it. HiCCUPS uses a Poisson model to detect regions of interest, which is a distribution for discrete data. After balancing a Hi-C interaction matrix, the data is not discrete but continuous. To be able to work with the Poisson distribution, the balancing of the values is reverted. To revert the data, and therefore, to fit the data to a distribution based on any assumption instead of fitting on the most probable one is a wrong approach. Moreover, the Poisson distribution on the raw Hi-C data tends to have an overdispersion, making it questionable if Poisson is the correct choice. HOMER [11] creates a relative contact matrix per chromosome and scans these for locally dense regions. HOMER does not

1 https://github.com/aidenlab/juicer

Compiled on: June 23, 2021.
Draft manuscript prepared by the author.

support standard file formats for Hi-C matrices like *cool* [12], which imposes the need to create all data from scratch, which is time-consuming and is a potential source of errors and inaccuracies. Chromosight [13] detects loops based on a pattern-matching algorithm. Cooltools[2] uses a reimplementation of the HiCCUPS algorithm; Fit-Hi-C [14] detects significant Hi-C contacts and provides a merging algorithm to detect DNA loops. Peakachu [15] uses a random forest approach trained on CTCF or H3K27ac data. Chromosight, cooltools, Peakachu and HiC-Explorer support the *cooler* file format. HOMER, Fit-Hi-C, and Peakachu are single-core that eliminates the opportunities of using parallelization techniques to improve runtime. GOTHIC [16] models the probability of two genomic locations to interact with each other as a mix of different biases and the chance of random interactions. The problem is that GOTHIC detects a large number of significant interactions but cannot detect only the enriched regions concerning their neighborhood. It is a good tool to detect significant interactions in a Hi-C interaction matrix, but it is not suitable for the specific task of chromatin loop detection. cLoops [17] uses a DBSCAN based approach combined with a local background to estimate the statistical significance of a loop. cLoops is mainly designed for HiChIP data and not for Hi-C. With HiChIP, protein binding sites can be investigated in their 3D context; however, similar to promoter capture Hi-C, only the targeted regions are enriched. The consequence of this is a Hi-C matrix with data only available at these enriched regions, and foreknowledge of potential loop locations is required. FastHiC [18] is a loop detection algorithm based on a hidden Markov random field Bayesian [19], which focuses on intra topological associated domain (TAD) loops in a range of 40 kb and therefore not on chromatin loops outside of TADs.

Here we present an algorithm that can detect Hi-C loops. Here, it is based on a continuous negative binomial distribution and is optimized for a high parallelization by assigning one thread per chromosome and multiple threads within a chromosome. This approach makes full use of the resources available in the last generation of multi-core CPU platforms.

Methods

According to Rao [8], most of the anchor points of detected loops lie within a range of 2 Mb. This insight can be used to decrease the search space in a biologically meaningful way and also reduces the computational burden, maintaining a low memory footprint at the same time. Moreover, interaction pairs with genomic distances which are too close to each other and therefore quite close to the main diagonal already have high interaction counts. It is, in many cases, unlikely that these pairs contribute enrichments in the context of their neighborhood. The high interaction count can explain this observation between two loci; they are closer in the one-dimensional space and close to the main diagonal. Specialized algorithms like FastHiC should be used to detect intra-TAD enrichments. A general problem for Hi-C interactions with few absolute counts is to determine if their interactions are true interactions or noise. These artifacts cannot be corrected by the used Hi-C interaction matrix correction algorithms like iterative correction and eigenvector decomposition (ICE) [9], or Knight-Ruiz (KR) [10]. These algorithms perform a matrix balancing and correct for an uneven distribution of the interaction counts per genomic position. The correction algorithms cannot decide and therefore filter out if interactions are true interactions or noise. All values below a given threshold are discarded, and noise is removed to account for these known problems in the Hi-C interaction data.

2 https://github.com/open2c/cooltools

Algorithm

A strict candidate selection is critical to reduce the computational complexity of the loop detection algorithm. A maximum loop size can be defined to restrict the search space (Figure 1B) to take the observation from Rao [8] into account. In Hi-C, the primary data structure is the symmetrical $n \times n$ interaction count matrix (ICM):

$$ICM = \begin{bmatrix} ic_{00} & \cdots & ic_{0n} \\ \vdots & \cdots & \vdots \\ ic_{n0} & \cdots & ic_{nn} \end{bmatrix} \quad (1)$$

The relative genomic distance is given by:

$$d = |i - j| \; for \; ic_{i,j} \quad (2)$$

And $ic_{i,j}$ as an element of Hi-C interaction matrix ICM.

As a first step, the interaction matrix ICM is transferred to an observed vs. expected matrix to normalize the differing interaction heights per genomic distance. The observed/expected matrix is named M^*. Each entry is defined as:

$$m^*_{i,j} = \frac{icm_{i,j}}{exp_d} \quad (3)$$

Different methods are offered to adjust differences in samples introduced. Hi-C is, in comparison to techniques like RNA-seq, a two-dimensional approach; all reads are chimeric. Spatially close DNA fragments are fixated with formaldehyde, digested, and ligated to create chimeric reads. These events should, in theory, happen uniformly in the whole genome; however, it might also depend on the sample and genome if this is the case. Therefore, not only one but three ways to compute the expected value are offered.

First, only non-zero contacts are considered:

$$exp\_nonzero_d = \frac{\sum ic_{i,j}}{|non - zero \; interactions \; d|} \quad (4)$$

Second, all contacts are considered:

$$exp\_with\_zero_d = \frac{\sum ic_{i,j}}{|all \; interactions \; d|} \quad (5)$$

And third, similar to HOMER's normalization, a correction for different occurring ligation events is offered:

$$exp\_ligation_d = exp\_nonzero_{i,j} * \frac{\sum(row_{ICM}(i)) * \sum(row_{ICM}(j))}{\sum(ICM)} \quad (6)$$

Candidate selection per genomic distance

To detect enriched Hi-C interactions, the observed/expected normalized Hi-C data is fitted per genomic distance d independently to a continuous negative binomial distribution (Figure 1C). Supplementary Figure 1 shows the value density distribution of different genomic distances and provides evidence for the chosen distribution assumption. In genome analysis, negative binomial functions have shown potential, for example, by DESeq2 [20]. The negative binomial function, and not the Poisson distribution, is used because the raw data of the genomic distances of chromosome 1 of GM12878 cell line at 10 kb indi-

cate overdispersion [21] in a majority of the distances (80.1%); therefore, the negative binomial distribution with an additional free parameter is the better choice (Supplementary Figure 2).

$$X_d \sim cNB_d(r_d, p_d) \; \forall d = |i - j| \quad (7)$$

Gamma functions must replace the factorial in the binomial coefficient as it is used by edgeR [22, 23] to make the discrete negative binomial function continuous:

$$\binom{k+r-1}{k} = \frac{(k+r-1)!}{(k!) * (k+r-1-k)!} = \frac{(k+r-1)!}{(k!) * (r-1)!} \quad (8)$$

The gamma function is defined for any $n \in \mathbb{N}$:

$$\Gamma(n) = (n-1)! \quad (9)$$

Moreover, the gamma function is defined for any $n \in \mathbb{R}_{>0}$:

$$\Gamma(n) = \int_0^\infty x^{n-1} * e^{-x} dx \quad (10)$$

With Equation (9), the binomial coefficient can be reformulated as:

$$\binom{k+r-1}{k} = \frac{\Gamma(k+r)}{\Gamma(k+1) * \Gamma(r)} \quad (11)$$

Which leads to the probability mass function for a 'continuous negative binomial distribution' with $\forall k \in \mathbb{R}_{>0}$ and $\forall r \in \mathbb{R}_{>0}$:

$$f(k, r, p) = \frac{\Gamma(k+r)}{\Gamma(k+1) * \Gamma(r)} p^k (1-p)^r \quad (12)$$

The p-value of observing a specific observed vs. expected value at the genomic distance d is given by the continuous negative binomial cumulative density function:

$$pvalue\ of\ m^*_{i,j} = P(x \geq m^*_{i,j}) = \begin{cases} 1 - CDF_d(m^*_{i,j}) & \text{if } m^*_{i,j} > 0. \\ 1 & \text{if i = 0.} \end{cases} \quad (13)$$

Only the observed vs. expected values with p-values smaller than an individual threshold per genomic distance are accepted as candidates (Figure 1D and 1E); these candidates are further filtered to remove candidates with too few absolute interactions. To reduce the amount of data to fit, the user can remove observed vs. expected values lower a threshold before the continuous negative binomial function is fitted. Moreover, an option to remove candidates by their interaction height is given too.

Loop peak detection

The entire neighborhood needs to be considered to detect enriched regions in a Hi-C interaction matrix. A neighborhood is a square of size n with the candidate element in its center; see Figure 1F. An enriched region needs to have an enriched interaction count in relation to the elements in its neighborhood. The neighborhood concept comes with a few issues: First, in one neighborhood, there can be multiple candidates detected from different, but next to each other located genomic distances. Second, if a candidate is significant for its genomic distance, it is not necessarily an enriched value for its neighborhood. Third, a single enriched interaction in a neighborhood is possible but is likely a false positive. Meaningful enriched interactions appear in groups and form a peak in the two-dimensional space, as shown in Figure 1F. All candidates in one neighborhood are pooled together to handle the first issue, only the candidate with the highest observed vs. expected value for one neighborhood is considered a representative of its neighborhood; all others are removed. The neighborhood is split into a peak and a background region to cover the second and third issues by considering the square around the candidate as the peak region and the neighborhood's remaining elements as the background, see Figure 1G. The neighborhood is further divided into the vertical region left and right from the peak, the horizontal region above and below the peak, and the bottom left corner; this is a similar approach to HiCCUPS [8]. The peak and neighborhood square sizes are defined by their inradius values, *peakWidth* and *windowSize*. All candidates which fulfill of the following condition are rejected as a loop: $mean(background) \geq mean(peak)$. This filtering step is necessary to address the candidate peak value as a singular outlier within the neighborhood. Furthermore, the Wilcoxon rank-sum test with H0 hypothesis background and peak regions have the same distribution with significance level p is used. The mentioned filter steps guarantee that only neighborhoods with a centering peak value are considered.

Analyses

The algorithm was tested on various cell types published by Rao 2014 to verify the chromatin loop detection algorithm results: GM12878, K562, IMR90, HUVEC, KBM7, NHEK, and HMEC. First, the parameter setting for HiCExplorer is investigated, and second, the detect loops of several algorithms are compared. HiCExplorer's implementation is tested against the HiCCUPS algorithm from the Juicer software, HOMER's loop detection, chromosight, cooltools *call-dots*, Fit-Hi-C and Peakachu. The algorithms of GOTHIC, cLoops, and FastHiC are not part of the comparison due to the algorithms' different focuses. The detected chromatin loop locations are correlated with binned protein peak locations of the 11-zinc finger protein CTCF created by ChIP-Seq. CTCF is a known loop binding factor [8] although not all peaks need to have CTCF attached [24], especially in the case of a gene or a polycomb-mediated loop [6]. An intersection of a detected chromatin loop region was accepted if at both loci CTCF was detected. CTCF was matched to the GM12878, HMEC, HUVEC, K562, and NHEK cell samples; for IMR90 and KBM7, no CTCF from the same source is provided. A downside of ChIP-Seq is the one-dimensionality. Two-dimensional data for CTCF, H3K27ac, SMC1, and RAD21 created by HiChIP and ChIA-PET were tested for the GM12878 data set to investigate how one-dimensionality affects the results.

HiCExplorer parameters

The parameters of HiCExplorer do influence the results of the algorithm. First, the threshold for the observed/expected values has the property that fewer loops are detected, the stricter it is. A threshold of 0.5 results in 12331 loops, a threshold of 1 in 12008, but a threshold of 1.5 and 2 results in 9147 and 6099 detected loops, respectively. The stricter the threshold, the more accurate the loops; however, the absolute number of detected loops is lower. The p-value for the continuous negative binomial functions has the same effect, the stricter the threshold, the fewer loops are detected, but they become more accurate. The two difficult to choose parameters are the peak window size and the neighborhood window size. The peak window size needs to be smaller, and the two values should not be too similar. A peak window size of 4 and a neighborhood window size of 5 lead to 2380 loops, but if the peak window size

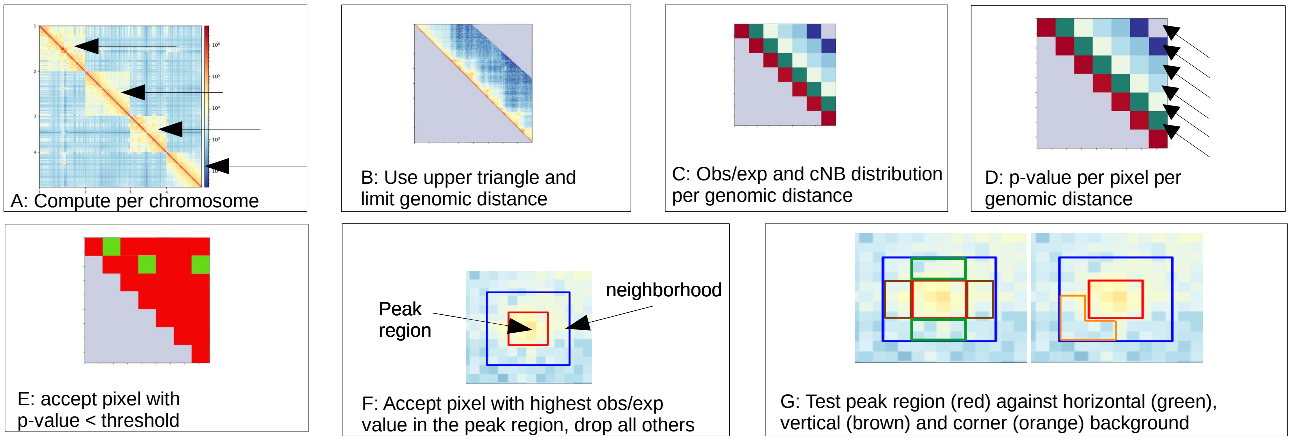

Figure 1. Graphical representation of the loop detection algorithm: A: Compute each chromosome independently. B: Accept an interaction if their relative distance is within: *0 < relative distance < maxLoopSize*. C: (Optional: Remove too low observed vs. expected value before fitting.) Fit cNB distribution per relative distance. D: Compute a p-value for each interaction. E: Reject the candidate if the p-value is too high or the interaction value is too small. F: Define neighborhood around an interaction. Accept as the candidate the one with the highest interaction. G: Apply testing of the peak region vs. vertical, horizontal, and bottom left neighborhood. Reject candidate if: a) maximum or mean of peak region is smaller than the maximum or mean of the neighborhood or b) the p-value computed by Wilcoxon rank-sum test comparing peak and neighborhood region is too high.

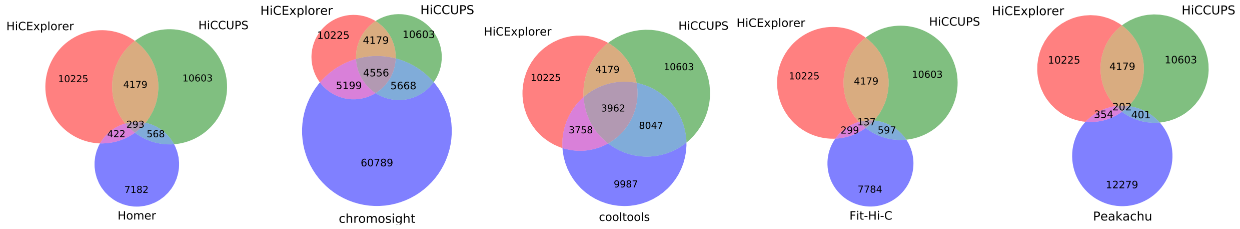

(a) HOMER **(b)** Chromosight **(c)** cooltools **(d)** Fit-Hi-C **(e)** Peakachu

Figure 2. Intersection of detected loops of HiCExplorer, HiCCUPS and either HOMER, chromosight, cooltools, Fit-Hi-C or Peakachu. HiCExplorer, HiCCUPS and cooltools have the highest relative intersection, chromosight the most intersected loops but detects with six times more interaction many false positives. Homer, Fit-Hi-C and Peakachu have only a minor intersection.

is reduced to 2, 9147 loops are detected. The same difference also exists between the sizes, but a peak window size of 4 and neighborhood size of 7, leads to a lower number of detected loops, 7269, with an equal level of accuracy, 0.70 vs. 0.69. The threshold for the peak region and the neighborhood test has an expected effect on loop detection. The stricter it is set, the fewer loops are detected, but the accuracy increases. The different methods to compute the expected value do not contribute to significant differences in the results. Supplementary Figure 3 and Supplementary Table 8 show the expected value based on all interactions (Equation 4) has the best accuracy (CTCF ChIP-Seq 0.71; CTCF ChIA-PET 0.64), the expected value based on the non-zero interactions (Equation 5) has the highest number of detected loops (14144) and provides more absolute correlated loop locations (CTCF ChIP-Seq 9352; CTCF ChIA-PET 7808). Last, the correction for ligation events as proposed by the HOMER (Equation 6) software shows the lowest accuracy (CTCF ChIP-Seq 0.58; CTCF ChIA-PET 0.48). The results depend on the data: The fewer reads a Hi-C matrix has, the sparser it is, and the fitted distributions are therefore more biased towards zero. With this, interactions with a lower interaction count have a lower p-value and might be detected. However, excluding the zero contacts from the distribution can lead to a bias in the other direction; interaction values that should be detected have a too high p-value and are therefore excluded from the computation.

For other cell lines published by Rao 2014, the situation is comparable (Supplementary Table 6). These cell lines have a different read coverage, ranging from 188 million to 1,800 million (Supplementary Table 7). For all cell lines, the number of detected candidates is of the same order of magnitude, which indicates a robust candidate selection with the chosen continuous negative binomial distributions. Another essential aspect of reducing the search space is the observation that peaks in Hi-C interaction matrices have a two-dimensional area and not single elements. Peaks are only detectable in the context of their local neighborhood, as the significance given by the continuous negative binomial distributions is not enough. The independent candidate selection per genomic distance leads to multiple candidates per neighborhood, and consequently, only the one with the highest observed/expected value can be considered the peak. The situation is different after testing the peak region (Supplementary Table 1). The number of detected loops differs between 3000 to 10,000 loops. The non-zero values and implicitly the read coverage per bin are considered to explain this different detection behavior; the higher the read coverage, the more regions are detected (see (Supplementary Table 1, 6 and 7). The candidate selection approach via the definition of a neighborhood makes the algorithm sensitive to the Hi-C interaction matrix's resolution. The lower the resolution, the smaller the neighborhood needs to be. Otherwise, the chances of having elements in the neighborhood, which are peaks or TADs, or even the main diagonal, are too high. Decreasing the size of the neighborhood creates, at the same time, another issue: the neighborhood and, therefore, the number of elements in the peak and background regions are becoming too less. This leads to non-significant test results and to the insight that the neighborhood size needs to be adjusted to the bin resolution of the Hi-C matrix, and second, a neighborhood should contain at least around 250 – 300 elements to produce

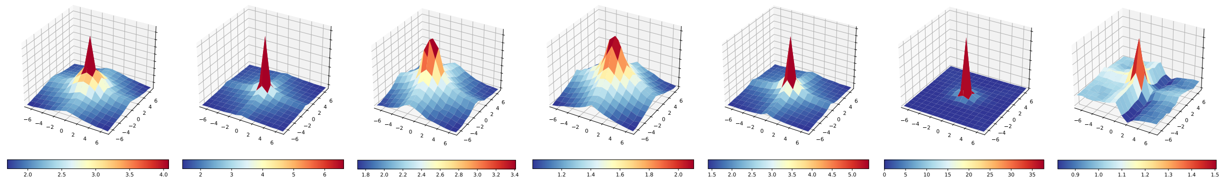

(a) HiCExplorer **(b)** HiCCUPS **(c)** HOMER **(d)** Chromosight **(e)** Cooltools **(f)** Fit-Hi-C **(g)** Peakachu

Figure 3. Aggregated loop locations of detected loops on GM12878, 10 kb resolution for the different detection algorithms. Aggregation with HiCExplorer's hicAggregateContacts.

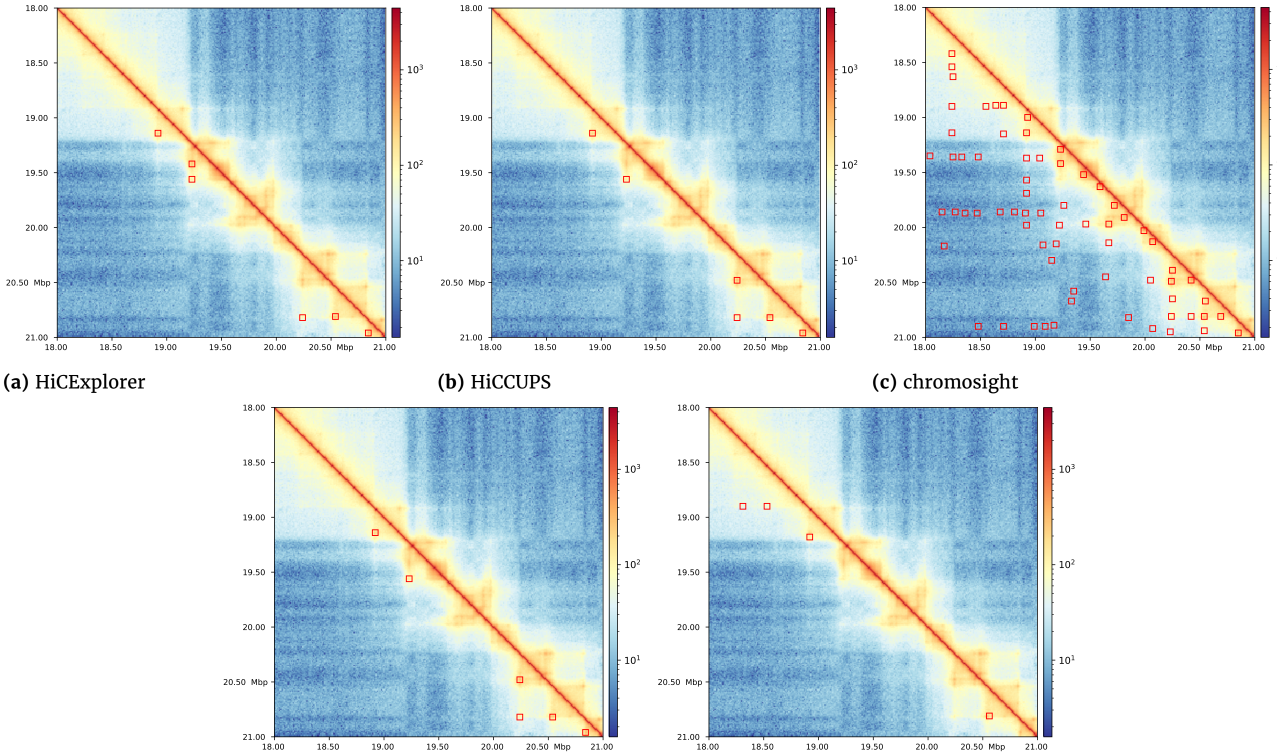

(a) HiCExplorer **(b)** HiCCUPS **(c)** chromosight **(d)** cooltools **(e)** Peakachu

Figure 4. The plot of chr1 18 - 22 Mb on GM12878 and highlighted the detected loops from each software. HiCExplorer, HiCCUPS, and cooltools show similar results. Chromosight detects many loops in noisy regions and lacks specificity. The four loops of Peakachu show a general issue of this algorithm: The first two loops (18 Mb region) are in a region without enrichment, and the two others slightly miss the enriched interactions by a few kilobases. HOMER and Fit-Hi-C are not detecting any loop in the area. Plot with HiCExplorer hicPlotMatrix.

valuable results.

Comparison to state of the art approaches

The number of detected enriched regions of HiCExplorer, HiCCUPS, HOMER, chromosight, cooltools, Fit-Hi-C and Peakachu differs between the samples. The detection rate is on a comparable level (Supplementary Table 1), except for chromosight and Peakachu. Chromosight detects significantly more loops with a very low p-value; however, as the loops' visualization (Figure 4) indicates, most detect loops are in very noisy regions, and it is questionable what chromosight exactly detects. The detected loops are correlated with CTCF and cohesin factors (Supplementary Table 2 and 5) to investigate the accuracy. The detect loops of HiCExplorer are on a comparable level to HiCCUPS and cooltools; on GM12878, HiCExplorer detects a similar amount of loops compared to HiCCUPS (8 Mb: 7298 vs. 7312) but is more specific (8 Mb: 0.71 vs. 0.68), but for example on HMEC HiCExplorer detect fewer loops (8 Mb: 3810 vs. 5350) and is less specific (8 Mb: 0.66 vs. 0.7). Cooltools detect on K562 fewer loops (8 Mb: 4081 vs. 3224) but is more specific (8 Mb: 0.69 vs. 0.71). The other tested cell lines HUVEC, HMEC, and NHEK present similar behavior. The results of HOMER and chromosight differ a lot in comparison to HiCExplorer, HiCCUPS, and cooltools. HOMER detects more absolute loops (except for GM12878 and KBM7), but it has a low accuracy over all cell lines. Chromosight detects from all testes approaches the most loops and has the highest number of loops correlated to CTCF. HiCExplorer, HiCCUPS, and cooltools can reach similar detection rates if the p-value thresholds are increased; however, the specificity for significantly enriched regions would be removed from the algorithms. Fit-Hi-C and Peakachu are special cases in this context. Both algorithms have a very low correlation rate to CTCF, Fit-Hi-C with 0.02 and Peakachu 0.17. The used method with ChIP-Seq data is biased and not available in a two-dimensional space. For this reason, we correlated the GM12878 cell line data additional with CTCF and RAD21 created with ChIA-PET and H3K27ac, and SMC1 created with HiChIP (Supplementary Table 5). The indication of the accuracy from the ChIP-Seq CTCF data is confirmed. HiCExplorer correlates best with 0.64 to CTCF ChIA-PET, HiCCUPS 0.61, cooltools 0.54, chromosight 0.11, HOMER 0.18, Fit-Hi-C 0.02 and Peakachu 0.05. RAD21 ChIA-PET correlates with HiCExplorer 0.25, HiC-

CUPS 0.22, and cooltools 0.17; all other tools correlate less than 0.03. H3K27ac HiChIP data correlate well for HiCExplorer with 0.86, but HiCCUPS is with 0.92 better. Cooltools is with 0.88 also high, chromosight and HOMER have a high correlation too (0.68; 0.74), but Fit-HiC and Peakachu have an inadequate performance (0.29, 0.39). The situation is similar for SMC1 HiChIP: HiCExplorer, HiCCUPS, cooltools, and HOMER correlate in more than 90% of the detected loops, chromosight falls back a bit with 0.77. Fit-Hi-C and Peakachu correlate with 0.34 and 0.5. It needs to be mentioned that the correlation with ChIP-Seq, ChIA-PET, and HiChIP data can only indicate the detected loops' quality. The loop structures representing gene or polycomb-mediated loops do not have CTCF at their anchor points, and the correlation can only be as good as the quality of the correlation data.

In comparison to HiCCUPS, HiCExplorer misses the 2% chromatin loops stated in Rao 2014 for genomic distances > 2 Mb, including inter-chromosomal enrichments. These inter-chromosomal enrichments are not detectable by HiCExplorer because each chromosome is computed independently. In our testing, also, HiCCUPS was not able to detect non-inter-chromosomal interactions. Recomputed results on GM12878 with HiCCUPS and three resolutions, 5 kb, 10 kb, and 25 kb, 17768 loops were detected, and 4910 have a distance greater than 2 Mb; on 10 kb out of 12865 loops, 2968 have a greater distance than 2 Mb. It is not entirely clear on which basis Rao 2014 states that only 2% of the loops are in a range greater than 2 Mb. However, if the correlated loops are computed on HiCCUPS data with all loops of distances greater than 2 Mb are removed, 6205 instead of 6354 loops can be correlated with CTCF. These findings support the restriction to a range of 2 Mb. Also, chromosight and cooltools show no significant difference in the number of detected and correlated loops between 2 Mb or 8 Mb of distance for most cells. If the restriction of the genomic distance between two loci is removed for HiCExplorer and all intra-chromosomal contacts are considered, the number of candidates to be tested increases by a factor of 10, but the number of accepted peaks increased slightly.

The intersection of detected peaks of HiCExplorer, HiCCUPS, HOMER, chromosight, cooltools, Fit-Hi-C and Peakachu is quite different (Figure 2). HiCExplorer with a search distance of 8 Mb shares ~ 46% of its loops with HiCCUPS genome-wide search and the restricted 8 Mb version on GM12878 cell line, e.g., only ~ 24% on HUVEC. HiCExplorer has the highest intersection of detect loops with chromosight, but chromosight also provides the highest number of detect loops. The intersection of detected loops with cooltools is similar to HiCCUPS; the number of intersecting loops with HOMER, Fit-Hi-C, and Peakachu is the lowest. HiCCUPS and cooltools show the highest intersecting numbers, chromosight profits from the high detection rate, while HOMER also has with HiCCUPS only a few hundred intersecting loops. The intersection of Fit-Hi-C and Peakachu with HiCExplorer and HiCCUPS is very low, and the results of the Peakachu publication cannot be confirmed. For Peakachu, we can assume this performance is directly connected to the provided trained models and their bad generalization ability. In the publication of Peakachu, the authors write they have used a probability threshold for a pixel between 90% and 97%. However, to detect a similar number of loops to have comparability, we had to use a score of 68%. For Fit-Hi-C, the authors of Peakachu have used a threshold of 10^{-5}, while we used 0.01 to have a few thousand loops detected.

The accumulated contacts of all detected loop locations on GM12878, displayed as a 3D plot in Figure 3, shows all algorithms detect enriched regions, but the neighborhood structure is very different. HiCExplorer detects a sharp peak with an enriched direct neighborhood; HiCCUPS and Fit-Hi-C have a very sharp peak with almost no neighborhood signal. HOMER and Chromosight detect broader peaks with a highly enriched neighborhood; cooltools has a sharp peak and a neighborhood structure that is slightly more enriched than HiCCUPS and slightly less than HiCExplorer. Last, Peakachu detects a sharp peak, has a neighborhood plateau on one side similar to the other algorithms but on the other side a sharp cliff. The visualizations indicate that a broad peak detection as HOMER and Chromosight provide, or a very sharp peak with no neighborhood signal, have a low correlation to CTCF based loops. The visualization of Peakachu's loop locations with the cliff can be interpreted as locations with a TAD border. This can be explained in the context of a learned model based on CTCF locations because CTCF is present at loop locations and TAD boundaries.

The proposed peak detection algorithm was tested on multiple datasets with a 10 kb resolution and is the fastest approach of all CPU-based approaches if the 2 Mb search space is considered. The HiCCUPS restricted mode on the GPU is slightly faster, for example, 0:56 min to 0:39 on NHEK. On the 8 Mb search distance range, HiCExplorer is also the fastest approach, except for GM12878 cell lines where HiCCUPS in the CPU-based version is faster. HiCExplorer is on GM12878 and 8 Mb search distance ~ 44% faster than chromosight (4:25 min vs. 6:22 min) and uses only 6.7 GB memory while chromosight consumes 39 GB. Moreover, HiCExplorer is two times faster than cooltools if only the loop detection is considered; if the necessary computation of expected values is added, it is almost 3.5 times faster (Supplementary Table 7). Chromosight is the fastest algorithm if only the divorced from the real world single-core performance is measured (Supplementary Table 9 and 10). Modern CPUs support up to 64 cores / 128 threads, and data analysis software should use the offered resources as well as possible. For this reason, HiCExplorers' hicDetectLoop does support the parallelization by the chromosomes and an intra-chromosomal parallelization. The structure of the data allows this: each chromosome can be computed independently, and each genomic distance normalization, distribution fitting, p-value computation too. For example, using 23 threads to compute each chromosome in parallel, and for each chromosome thread, ten additional threads compute all intra-chromosomal computations in parallel. This would lead to a usage of 230 parallel threads. Not all threads are used at the same time, therefore, a good utilization is achieved. However, modern CPUs with core/thread counts of 64 / 128 can be fully utilized with this approach. The two algorithms, Fit-Hi-C and Peakachu, provide only a single core implementation. Their runtimes are on the GM12878 data with 4:46 hours and 7:03 hours by far the slowest and consume at the same time a high amount of memory. HOMER is, in all scenarios, the third slowest algorithm and consumes the most memory. It has the side effect that, for example, the GM12878 dataset could only be computed using one single-core because the memory consumption was already around 100 GB. The chosen approach by the developers of HOMER to not support any binary file format to store and access the Hi-C interaction matrix-like Juicer's *hic* or the from many other investigated tools supported *cooler* file format [12], results in a computation based on text files and raw data, and a very poor runtime and memory performance.

Discussion

The search space of an algorithm is the dominating factor for its accuracy and performance. Therefore, pruning it should be the primary goal of newly designed algorithms. In theory, brute force solutions like HiCCUPS with no restrictions to the search space can detect all possible enriched regions, but at the cost of hardware demanding implementation. HiCCUPS

solved this by the massively parallel computational resources via GPGPU. The limitation of the search space to a genomic distance of 2 Mb has only a tiny impact on the detected peaks. HOMER, however, has no limitations on the search space, detects less number of loops, and the detected ones have a significantly lower correlation over all samples to CTCF. Moreover, HOMER supports a parallel computation per chromosome like HiCExplorer but is significantly slower than all other solutions and extensively uses more memory per core. HOMER's poor runtime performance can be explained by computing on raw data, while all other approaches use precomputed interaction matrices. Chromosight is a fast detection approach and provides the fastest single-core performance; however, it lacks specificity and detects many loops that should be considered noise, even if these loops are provided with a high significance. Cooltools, with its reimplementation of the HiCCUPS approach, is fast and more flexible by providing a genome distance search. The results are good, but it raises questions why they are not more similar to Juicer's HiCCUPS results if both use the same algorithm. An overview of all algorithms properties is listed in Supplementary Table 11. Furthermore, it could be shown that the sparsity and therefore read coverage of a Hi-C interaction matrix significantly influence the detection of peaks in their neighborhood. The sparser a Hi-C interaction matrix is, the more likely it is that possible valid regions detected by the continuous negative binomial distribution filtering are rejected by Wilcoxon rank-sum test. The large number of different detect loops and the high correlation rates to CTCF can be explained in multiple ways. The correlation to CTCF is caused by biology itself. Not all loops have CTCF as a binding protein at its anchors; gene-loops or polycomb-mediated loops lack it. All the algorithms detect enrichments in the Hi-C data, which are interpreted as loops but do not need to be loops necessarily. The enrichments can also be noise in the data, or interactions are not related to CTCF. Second, the Hi-C data is created with in-situ Hi-C and has a higher noise level than newer approaches like Arima Hi-C[3]. Detections of loops in noisy areas cause the competing algorithms' low intersection values, especially chromosight detects more noise than loops.

Availability of source code and requirements

HiCExplorer is licensed under GPLv3 and is available on Github (https://github.com/deeptools/HiCExplorer/) or as a conda package in the bioconda channel [25]. HiCExplorer is implemented in Python 3.6, 3.7. and 3.8 for Linux and macOS.

Availability of supporting data and materials

Hi-C data: GSE63525; Rao et al. [8]. CTCF for: Gm12878 from GSM935611; Hmec from GSM749753; Huvec from GSM749749; K562 from GSM733719 and Nhek from GSM733636. CTCF ChIA-PET (GSM1872886); H3K27ac HiChIP (GSE101498), SMC1 HiChIP (GSE80820), and RAD21 ChIA-PET (GSM1436265).

Declarations

Competing Interests

The author(s) declare that they have no competing interests.

3 https://arimagenomics.com/

Funding

German Federal Ministry of Education and Research [031 A538A de.NBI-RBC awarded to R.B.]; German Federal Ministry of Education and Research [031 L0101C de.NBI-epi awarded to B.G.]. R.B. was supported by the German Research Foundation (DFG) under Germany's Excellence Strategy (CIBSS - EXC-2189 - Project ID 390939984).

Author's Contributions

JW: Designed and implemented the presented algorithm and wrote the manuscript. RB: contributed to the manuscript. BG: contributed to the manuscript.

Acknowledgements

We thank Simon Bray and Anup Kumar for proofreading the manuscript.

References

1. Dekker J, Rippe K, Dekker M, Kleckner N. Capturing chromosome conformation. science 2002;295(5558):1306–1311. [PubMed:11847345] [doi:10.1126/science.1067799].
2. Simonis M, Klous P, Splinter E, Moshkin Y, Willemsen R, De Wit E, et al. Nuclear organization of active and inactive chromatin domains uncovered by chromosome conformation capture-on-chip (4C). Nature genetics 2006;38(11):1348. [PubMed:17033623] [doi:10.1038/ng1896].
3. Zhao Z, Tavoosidana G, Sjölinder M, Göndör A, Mariano P, Wang S, et al. Circular chromosome conformation capture (4C) uncovers extensive networks of epigenetically regulated intra-and interchromosomal interactions. Nature genetics 2006;38(11):1341. [PubMed:17033624] [doi:10.1038/ng1891].
4. Dostie J, Richmond TA, Arnaout RA, Selzer RR, Lee WL, Honan TA, et al. Chromosome Conformation Capture Carbon Copy (5C): a massively parallel solution for mapping interactions between genomic elements. Genome research 2006;16(10):1299–1309. [PubMed:16954542] [PubMed Central:PMC1581439] [doi:10.1101/gr.5571506].
5. Lieberman-Aiden E, Van Berkum NL, Williams L, Imakaev M, Ragoczy T, Telling A, et al. Comprehensive mapping of long-range interactions reveals folding principles of the human genome. Science 2009 oct;326(5950):289–293. http://www.ncbi.nlm.nih.gov/pubmed/19815776http://www.pubmedcentral.nih.gov/articlerender.fcgi?artid=PMC2858594, [PubMed:19815776] [PubMed Central:PMC2858594] [doi:10.1126/science.1181369].
6. Bonev B, Cavalli G. Organization and function of the 3D genome. Nature Reviews Genetics 2016;17(11):661. [PubMed:28704353] [doi:10.1038/nrg.2016.147].
7. Tan-Wong SM, Zaugg JB, Camblong J, Xu Z, Zhang DW, Mischo HE, et al. Gene loops enhance transcriptional directionality. Science 2012;338(6107):671–675.
8. Rao SSP, Huntley MH, Durand NC, Stamenova EK. A 3D Map of the Human Genome at Kilobase Resolution Reveals Principles of Chromatin Looping. Cell 2014;159(7):1665–1680. http://dx.doi.org/10.1016/j.cell.2014.11.021, [PubMed:25497547] [PubMed Central:PMC5635824] [doi:10.1016/j.cell.2014.11.021].
9. Imakaev M, Fudenberg G, McCord RP, Naumova N, Goloborodko A, Lajoie BR, et al. Iterative correction of Hi-C data reveals hallmarks of chromosome

organization. Nature Methods 2012 sep;9(10):999–1003. http://www.nature.com/doifinder/10.1038/nmeth.2148, [PubMed:22941365] [PubMed Central:PMC3816492] [doi:10.1038/nmeth.2148].
10. Knight PA, Ruiz D. A fast algorithm for matrix balancing. IMA Journal of Numerical Analysis 2013;33(3):1029–1047. [doi:10.1093/imanum/drs019].
11. Heinz S, Benner C, Spann N, Bertolino E, Lin YC, Laslo P, et al. Simple Combinations of Lineage-Determining Transcription Factors Prime cis-Regulatory Elements Required for Macrophage and B Cell Identities. Molecular Cell 2010 may;38(4):576–589. https://www.sciencedirect.com/science/article/pii/S1097276510003667?via{%}3Dihub, [PubMed:20513432] [PubMed Central:PMC2898526] [doi:10.1016/j.molcel.2010.05.004].
12. Abdennur N, Mirny LA. Cooler: scalable storage for Hi-C data and other genomically labeled arrays. Bioinformatics 2020;36(1):311–316.
13. Matthey-Doret C, Baudry L, Breuer A, Montagne R, Guiglielmoni N, Scolari V, et al. Computer vision for pattern detection in chromosome contact maps. Nature communications 2020;11(1):1–11.
14. Kaul A, Bhattacharyya S, Ay F. Identifying statistically significant chromatin contacts from Hi-C data with FitHiC2. Nature protocols 2020;15(3):991–1012.
15. Salameh TJ, Wang X, Song F, Zhang B, Wright SM, Khunsriraksakul C, et al. A supervised learning framework for chromatin loop detection in genome-wide contact maps. Nature communications 2020;11(1):1–12.
16. Mifsud B, Martincorena I, Darbo E, Sugar R, Schoenfelder S, Fraser P, et al. GOTHiC, a probabilistic model to resolve complex biases and to identify real interactions in Hi-C data. PloS one 2017;12(4).
17. Cao Y, Chen Z, Chen X, Ai D, Chen G, McDermott J, et al. Accurate loop calling for 3D genomic data with cLoops. Bioinformatics 2020;36(3):666–675.
18. Xu Z, Zhang G, Wu C, Li Y, Hu M. FastHiC: a fast and accurate algorithm to detect long-range chromosomal interactions from Hi-C data. Bioinformatics 2016;32(17):2692–2695.
19. Xu Z, Zhang G, Jin F, Chen M, Furey TS, Sullivan PF, et al. A hidden Markov random field-based Bayesian method for the detection of long-range chromosomal interactions in Hi-C data. Bioinformatics 2016;32(5):650–656.
20. Love MI, Huber W, Anders S. Moderated estimation of fold change and dispersion for RNA-seq data with DESeq2. Genome biology 2014;15(12):1–21.
21. Cameron AC, Trivedi PK. Regression-based tests for overdispersion in the Poisson model. Journal of econometrics 1990;46(3):347–364.
22. Robinson MD, McCarthy DJ, Smyth GK. edgeR: a Bioconductor package for differential expression analysis of digital gene expression data. Bioinformatics 2010;26(1):139–140.
23. McCarthy DJ, Chen Y, Smyth GK. Differential expression analysis of multifactor RNA-Seq experiments with respect to biological variation. Nucleic acids research 2012;40(10):4288–4297.
24. Andrey G, Schöpflin R, Jerković I, Heinrich V, Ibrahim DM, Paliou C, et al. Characterization of hundreds of regulatory landscapes in developing limbs reveals two regimes of chromatin folding. Genome research 2017;27(2):223–233. [PubMed:27923844] [PubMed Central:PMC5287228] [doi:10.1101/gr.213066.116].
25. Grüning B, Dale R, Sjödin A, Chapman BA, Rowe J, Tomkins-Tinch CH, et al. Bioconda: sustainable and comprehensive software distribution for the life sciences. Nature methods 2018;15(7):475. [PubMed:29967506] [doi:10.1038/s41592-018-0046-7].

Supplement

Click here to access/download
Supplementary Material
supplement.pdf
