# Supplementary material for: Loop detection using Hi-C data with HiCExplorer
Source: Gigascience. 2022 Jul 9;11:giac061. doi: 10.1093/gigascience/giac061 (PMC9270730; doi:10.1093/gigascience/giac061)
Supplement: giac061_GIGA-D-21-00069_Revision_3 [file giac061_giga-d-21-00069_revision_3.pdf]

GigaScience
Loop detection using Hi-C data with HiCExplorer
--Manuscript Draft--

**Manuscript Number:**	GIGA-D-21-00069R3
**Full Title:**	Loop detection using Hi-C data with HiCExplorer
**Article Type:**	Technical Note
**Funding Information:**	Bundesministerium für Bildung, Wissenschaft, Forschung und Technologie (031 A538A de.NBI-RBC) — Prof. Dr. Rolf Backofen<br>Bundesministerium für Bildung, Wissenschaft, Forschung und Technologie (031 L0101C de.NBI-epi) — Dr. Björn Grüning<br>Deutsche Forschungsgemeinschaft (CIBSS - EXC-2189 - Project ID 390939984) — Prof. Dr. Rolf Backofen
**Abstract:**	Background: Chromatin loops are an essential factor in the structural organization of the genome; however, their<br><br>detection in Hi-C interaction matrices is a challenging and compute-intensive task. The approach presented here,<br><br>integrated into the HiCExplorer software, shows a chromatin loop detection algorithm that applies a strict candidate<br><br>selection based on continuous negative binomial distributions and performs a Wilcoxon rank-sum test to detect enriched<br><br>Hi-C interactions.<br>Results: HiCExplorer's loop detection has a high detection rate and accuracy. It is the fastest available<br><br>CPU implementation and utilizes all threads offered by modern multi-core platforms.<br>Conclusions: HiCExplorer's method to detect loops by using a continuous negative binomial function combined with the donut approach from<br><br>HiCCUPS leads to reliable and fast computation of loops. All the loop-calling algorithms investigated provide differing<br><br>results, which intersect by ~ 50% at most. The tested in-situ Hi-C data contains a large amount of noise; achieving<br><br>better agreement between loop calling algorithms will require cleaner Hi-C data and therefore future improvements to<br><br>the experimental methods which generate the data.
**Corresponding Author:**	Joachim Wolff<br>Albert-Ludwigs-Universität Freiburg: Albert-Ludwigs-Universitat Freiburg<br>Freiburg im Breisgau, Baden-Württemberg GERMANY
**Corresponding Author Secondary Information:**	
**Corresponding Author's Institution:**	Albert-Ludwigs-Universität Freiburg: Albert-Ludwigs-Universitat Freiburg
**Corresponding Author's Secondary Institution:**	
**First Author:**	Joachim Wolff, Dr.
**First Author Secondary Information:**	

Powered by Editorial Manager® and ProduXion Manager® from Aries Systems Corporation

Order of Authors:	Joachim Wolff, Dr.
	Rolf Backofen, Dr.
	Björn Grüning, Dr.
Order of Authors Secondary Information:	
Response to Reviewers:	Dear editorial board,<br><br>I hereby submit the minor revision with the requested changes.<br><br>Your sincerely,<br><br>Joachim Wolff
Additional Information:	
Question	Response
Are you submitting this manuscript to a special series or article collection?	No
**Experimental design and statistics**<br><br>Full details of the experimental design and statistical methods used should be given in the Methods section, as detailed in our Minimum Standards Reporting Checklist. Information essential to interpreting the data presented should be made available in the figure legends.<br><br>Have you included all the information requested in your manuscript?	Yes
**Resources**<br><br>A description of all resources used, including antibodies, cell lines, animals and software tools, with enough information to allow them to be uniquely identified, should be included in the Methods section. Authors are strongly encouraged to cite Research Resource Identifiers (RRIDs) for antibodies, model organisms and tools, where possible.<br><br>Have you included the information requested as detailed in our Minimum Standards Reporting Checklist?	Yes
**Availability of data and materials**	Yes

Powered by Editorial Manager® and ProduXion Manager® from Aries Systems Corporation

All datasets and code on which the conclusions of the paper rely must be either included in your submission or deposited in publicly available repositories (where available and ethically appropriate), referencing such data using a unique identifier in the references and in the "Availability of Data and Materials" section of your manuscript.<br><br>Have you have met the above requirement as detailed in our Minimum Standards Reporting Checklist?	

Manuscript

Click here to access/download;Manuscript;manuscript_R3.pdf

GigaScience, XXXX, 1–8

doi: xx.xxxx/xxxx
Manuscript in Preparation
Technical Note

TECHNICAL NOTE

Loop detection using Hi-C data with HiCExplorer

Joachim Wolff[1,2,*], Rolf Backofen[1,3] and Björn Grüning[1]

[1]Bioinformatics Group, Department of Computer Science, University of Freiburg, Georges-Köhler-Allee 106, 79110 Freiburg, Germany and [2]Friedrich Miescher Institut for Biomedical Research, Maulbeerstrasse 66, 4058 Basel, Switzerland and [3]Signalling Research Centres CIBSS, University of Freiburg, Schänzlestr. 18, 79104 Freiburg, Germany

[*]wolffj@informatik.uni-freiburg.de

Abstract

Background: Chromatin loops are an essential factor in the structural organization of the genome; however, their detection in Hi-C interaction matrices is a challenging and compute-intensive task. The approach presented here, integrated into the HiCExplorer software, shows a chromatin loop detection algorithm that applies a strict candidate selection based on continuous negative binomial distributions and performs a Wilcoxon rank-sum test to detect enriched Hi-C interactions. **Results:** HiCExplorer's loop detection has a high detection rate and accuracy. It is the fastest available CPU implementation and utilizes all threads offered by modern multi-core platforms. **Conclusions:** HiCExplorer's method to detect loops by using a continuous negative binomial function combined with the donut approach from HiCCUPS leads to reliable and fast computation of loops. All the loop-calling algorithms investigated provide differing results, which intersect by ~ 50% at most. The tested in-situ Hi-C data contains a large amount of noise; achieving better agreement between loop calling algorithms will require cleaner Hi-C data and therefore future improvements to the experimental methods which generate the data.

Key words: Hi-C, Hi-C loop detection, DNA loops

Introduction

Many algorithms are currently available for loop detection in Hi-C data. HiCCUPS uses a *donut algorithm*, which considers all elements of a Hi-C interaction matrix as peaks and tests if the region around them is significantly different from the neighboring interactions. HiCCUPS is part of the software Juicer[1], and the implementation requires a general-purpose GPU (GPGPU), which imposes a barrier for users without access to Nvidia GPUs. However, an experimental CPU-based implementation has also been released. Algorithms such as iterative correction and eigenvector decomposition (ICE) [1], or Knight-Ruiz (KR) [2] are widely used in Hi-C data analysis for balancing Hi-C matrices, but the loop detection algorithm of HiCCUPS uses a different approach. HiCCUPS employs a Poisson model, which is a distribution for discrete data, to detect regions of interest. After balancing a Hi-C interaction matrix, the data is no longer discrete, but continuous. In order to work with the Poisson distribution, the balancing of the values is reverted. This procedure is methodologically questionable, as it involves manipulation of the data to fit the requirements of a particular distribution, rather than fitting on the distribution which is most probable or suitable. Moreover, the Poisson distribution on the raw Hi-C data tends to have an overdispersion, which suggests Poisson is not the best choice. HOMER [3] creates a relative contact matrix per chromosome and scans these for locally dense regions. HOMER does not support standard file formats for Hi-C matrices like *cool* [4], which forces the user to create all data from scratch, a time-consuming process and a potential source of errors and inaccuracies. Chromosight [5] detects loops based on a pattern-matching algo-

1 https://github.com/aidenlab/juicer

Compiled on: March 24, 2022.
Draft manuscript prepared by the author.

rithm. Cooltools[2] uses a reimplementation of the HiCCUPS algorithm; Fit-Hi-C [6] detects significant Hi-C contacts and provides a merging algorithm to detect DNA loops. Peakachu [7] uses a random forest approach trained on CTCF or H3K27ac data. Chromosight, cooltools, Peakachu, and HiCExplorer support the *cooler* file format. HOMER, Fit-Hi-C, and Peakachu do not utilize parallelization techniques to improve runtime, running only on a single core.

Here we present an algorithm that can detect Hi-C loops. It is based on a continuous negative binomial distribution and is highly parallelized, assigning one thread per chromosome and parallelizing further using multiple threads within a chromosome. This approach makes full use of the resources available in the last generation of multi-core CPU platforms.

Methods

According to Rao [8], most of the anchor points of detected loops lie within a range of 2 Mb. This insight can be used to decrease the search space in a biologically meaningful way and also to reduce the computational burden, while at the same time maintaining a low memory footprint. Moreover, interaction pairs with genomic distances which are too close to each other, corresponding to points in the Hi-C matrix close to the main diagonal, already have high interaction counts. It is, in many cases, unlikely that these pairs contribute enrichments in the context of their neighborhood. The high interaction count can explain this observation between two loci; they are closer in one-dimensional space and close to the main diagonal. Specialized algorithms like FastHiC should be used to detect intra-TAD enrichments. A general problem for Hi-C interactions with few absolute counts is determining whether their interactions are true interactions or noise. These artifacts cannot be corrected by the commonly-used Hi-C interaction matrix correction algorithms such as iterative correction and eigenvector decomposition (ICE) [1], or Knight-Ruiz (KR) [2]. These algorithms perform a matrix balancing and correct for an uneven distribution of the interaction counts per genomic position. The correction algorithms are unable to distinguish and therefore filter true interactions from noise. All values below a given threshold are discarded, and noise is removed to account for these known problems in the Hi-C interaction data.

Algorithm

A strict candidate selection is critical to reducing the computational complexity of the loop detection algorithm. A maximum loop size can be defined to restrict the search space to take the previously-mentioned observation from Rao [8] into account. In Hi-C, the primary data structure is the symmetrical $n \times n$ interaction count matrix (ICM):

$$ICM = \begin{bmatrix} ic_{00} & \cdots & ic_{0n} \\ \vdots & \cdots & \vdots \\ ic_{n0} & \cdots & ic_{nn} \end{bmatrix} \quad (1)$$

The relative genomic distance is given by:

$$d = |i - j| \; for \; ic_{i,j} \quad (2)$$

where $ic_{i,j}$ is an element of Hi-C interaction matrix ICM.

2 https://github.com/open2c/cooltools

As a first step, the interaction matrix ICM is transferred to an observed vs. expected matrix M^* to normalize the differing interaction heights per genomic distance. Each element m of M^* is defined as:

$$m^*_{i,j} = \frac{icm_{i,j}}{exp_d} \quad (3)$$

Different methods are offered to adjust differences in the samples introduced. Hi-C is, in comparison to techniques like RNA-seq, a two-dimensional approach; all reads are chimeric. The term chimeric in the context of Hi-C should be understood as reads which are ligated from two different locations in the genome. This is achieved by fixation of spatially close DNA fragments with formaldehyde, followed by digestion and ligation to create chimeric reads. These events should, in theory, happen uniformly in the whole genome; however, whether this is the case depends on the particular sample and genome studied. Therefore, three different ways to compute the expected value are offered. Note that the observed/expected matrix normalization step was not included in the initial version of this publication released on bioRxiv [9], but was described in the authors dissertation [10].

First, only non-zero contacts are considered:

$$exp\_nonzero_d = \frac{\sum ic_{i,j}}{|non - zero \; interactions \; d|} \quad (4)$$

Second, all contacts are considered:

$$exp\_with\_zero_d = \frac{\sum ic_{i,j}}{|all \; interactions \; d|} \quad (5)$$

And third, similar to HOMER's normalization, a correction for different occurring ligation events is offered:

$$exp\_ligation_d = exp\_nonzero_{i,j} * \frac{\sum(row_{ICM}(i)) * \sum(row_{ICM}(j))}{\sum(ICM)} \quad (6)$$

Candidate selection per genomic distance

To detect enriched Hi-C interactions, the observed/expected normalized Hi-C data is fitted per genomic distance d independently to a continuous negative binomial distribution. Supplementary Figure 1 shows the value density distribution of different genomic distances and provides evidence for the chosen distribution assumption. The negative binomial function, rather than the Poisson distribution, is used because the raw data of the genomic distances of chromosome 1 of GM12878 cell line at 10 kb indicate overdispersion [11] in a majority of the distances (80.1%); therefore, the negative binomial distribution with an additional free parameter is the better choice (Supplementary Figure 2).

$$X_d \sim cNB_d(r_d, p_d) \; \forall d = |i - j| \quad (7)$$

Gamma functions must replace the factorial in the binomial coefficient as used by edgeR [12, 13] to make the discrete negative binomial function continuous:

$$\binom{k + r - 1}{k} = \frac{(k + r - 1)!}{(k!) * (k + r - 1 - k)!} = \frac{(k + r - 1)!}{(k!) * (r - 1)!} \quad (8)$$

The gamma function is defined for any $n \in \mathbb{N}$:

$$\Gamma(n) = (n - 1)! \quad (9)$$

Moreover, the gamma function is defined for any $n \in \mathbb{R}_{>0}$:

$$\Gamma(n) = \int_0^\infty x^{n-1} * e^{-x} dx \tag{10}$$

With Equation (9), the binomial coefficient can be reformulated as:

$$\binom{k+r-1}{k} = \frac{\Gamma(k+r)}{\Gamma(k+1) * \Gamma(r)} \tag{11}$$

which leads to the probability mass function for a 'continuous negative binomial distribution' with $\forall k \in \mathbb{R}_{>0}$ and $\forall r \in \mathbb{R}_{>0}$:

$$f(k, r, p) = \frac{\Gamma(k+r)}{\Gamma(k+1) * \Gamma(r)} p^k (1-p)^r \tag{12}$$

The p-value of observing a specific observed vs. expected value at the genomic distance d is given by the continuous negative binomial cumulative density function:

$$pvalue\ of\ m^*_{i,j} = P(x \geq m^*_{i,j}) = \begin{cases} 1 - CDF_d(m^*_{i,j}) & \text{if } m^*_{i,j} > 0. \\ 1 & \text{if i = 0.} \end{cases} \tag{13}$$

Only the observed vs. expected values with p-values smaller than an individual threshold per genomic distance are accepted as candidates; these candidates are further filtered to remove candidates with too few absolute interactions. To reduce the amount of data to fit, the user can remove observed vs. expected values below a threshold before the continuous negative binomial function is fitted. Moreover, an option to remove candidates by their interaction height is also provided.

Loop peak detection

The entire neighborhood needs to be considered to detect enriched regions in a Hi-C interaction matrix. A neighborhood is a square of size n with the candidate element in its center. An enriched region needs to have an enriched interaction count in relation to the elements in its neighborhood. The neighborhood concept comes with a few issues: first, within a single neighborhood, there can be multiple candidate loops detected from different but adjacent genomic distances. Second, if a candidate is significant for its genomic distance, it is not necessarily an enriched value for its neighborhood. Third, a single enriched interaction in a neighborhood is possible, but is likely to be a false positive. Meaningful enriched interactions appear in groups and form a peak in the two-dimensional space. All candidates in one neighborhood are pooled together to handle the first issue, only the candidate with the highest observed vs. expected value for one neighborhood is considered a representative of its neighborhood; all others are removed. The neighborhood is split into a peak and a background region to cover the second and third issues by considering the square around the candidate as the peak region and the neighborhood's remaining elements as the background. The neighborhood is further divided into the vertical region left and right from the peak, the horizontal region above and below the peak, and the bottom left corner; this is a similar approach to HiCCUPS [8]. The peak and neighborhood square sizes are defined by their inradius values, *peakWidth* and *windowSize*. All candidates which fulfil the condition $mean(background) \geq mean(peak)$ are rejected as a loop. This filtering step is necessary to address the situation where a candidate peak value is a singular outlier within the neighborhood. Furthermore, the Wilcoxon rank-sum test is used, with the H0 hypothesis that the background and peak regions have the same distribution with significance level p. As background, the vertical and horizontal area mentioned above, and the bottom left corner, are independently tested against the peak region. Note in the initial version of this publication released on bioRxiv [9] only the peak vs. the entire neighborhood region was tested. The filter steps described guarantee that only neighborhoods with a centering peak value are considered.

Analyses

The algorithm was tested on various cell types published by Rao 2014 [8] to verify the chromatin loop detection algorithm results: GM12878, K562, IMR90, HUVEC, KBM7, NHEK, and HMEC. First, the parameter setting for HiCExplorer is investigated, and second, the loop detection results of several algorithms are compared. HiCExplorer's implementation is tested against the HiCCUPS algorithm from the Juicer software, HOMER's loop detection, chromosight, cooltools' *call-dots*, Fit-Hi-C, and Peakachu. The algorithms of GOTHIC, cLoops, and FastHiC are not considered, due to the differing focus of these algorithms. The detected chromatin loop locations are correlated with binned protein peak locations of the 11-zinc finger protein CTCF identified by ChIP-Seq. CTCF is a known loop binding factor [8] although not all peaks need to have CTCF attached [14], especially in the case of a gene or a polycomb-mediated loop [15]. In order to test the algorithms mentioned above, the detected chromatin loops were accepted as true if CTCF was detected at both loci, otherwise rejected. CTCF was matched to the GM12878, HMEC, HUVEC, K562, and NHEK cell samples; for IMR90 and KBM7, no CTCF from the same source is provided. A downside of ChIP-Seq is the one-dimensionality. In addition, therefore, two-dimensional data for CTCF, H3K27ac, SMC1, and RAD21 created by HiChIP and ChIA-PET were tested for the GM12878 data set to investigate how one-dimensionality affects the results.

HiCExplorer parameters

The parameters of HiCExplorer have an influence on the results of the algorithm. First, the threshold for the observed/expected values is negatively correlated with the number of detected loops. A threshold of 0.5 results in 12331 loops, a threshold of 1 in 12008, but a threshold of 1.5 and 2 results in 9147 and 6099 detected loops, respectively. The stricter the threshold, the more accurate the loops; however, the number of detected loops is lower. The p-value for the continuous negative binomial functions has the same effect: the stricter the threshold, the fewer loops are detected, but they become more accurate, as measured by CTCF correlation. Choosing good values for the peak window size and the neighborhood window size parameters presents some difficulty. The peak window size should be the smaller of the two, and the two values should not be too similar. A peak window size of 4 and a neighborhood window size of 5 leads to 2380 loops, but if the peak window size is reduced to 2, 9147 loops are detected. Increasing the two parameters by the same amount, to a peak window size of 4 and neighborhood size of 7, such that the same difference between the values is maintained, leads to a lower number of detected loops, 7269, with an equal level of accuracy, 0.70 vs. 0.69. The threshold for the peak region and the neighborhood test has an expected effect on loop detection. The stricter it is set, the fewer loops are detected, but the accuracy increases. The different methods provided for computing the expected value do not contribute to significant differences in the results. Supplementary Figure 3 and Supplementary Table 6 show the expected value based on all interactions (Equation 4) has the best

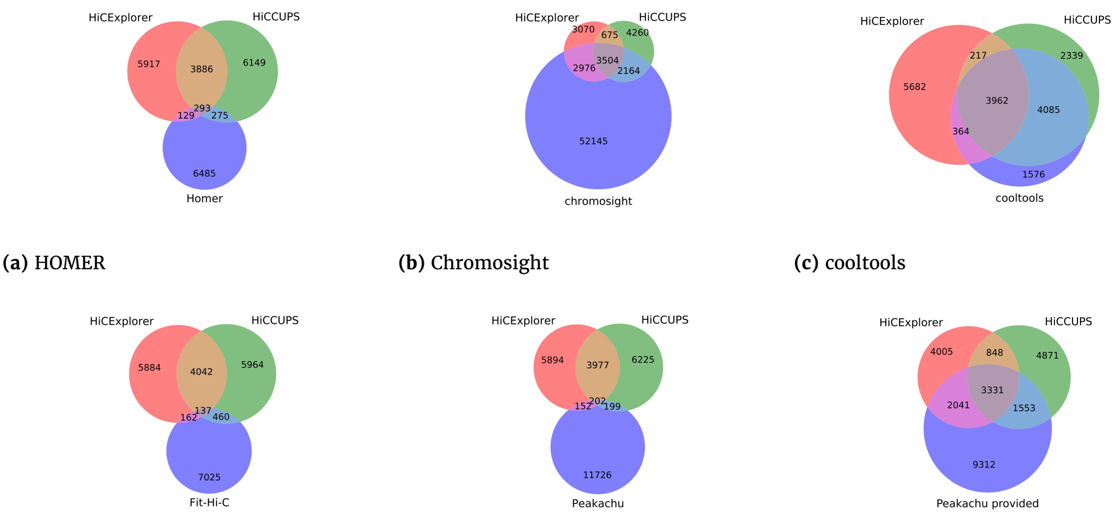

(a) HOMER **(b)** Chromosight **(c)** cooltools

(d) Fit-Hi-C **(e)** Peakachu **(f)** Peakachu (author data)

Figure 1. Intersection of detected loops of HiCExplorer, HiCCUPS and either HOMER, chromosight, cooltools, Fit-Hi-C or Peakachu. HiCExplorer, HiCCUPS, and cooltools have the highest relative intersection. Chromosight has the most intersected loops, but detects many false positives, predicting six times more interactions. Homer, Fit-Hi-C, and Peakachu have only a minor intersection. Last, the loop results of Peakachu, as published by the authors (subfigure **f**), shows a higher overlap with the detected loops of HiCExplorer and HiCCUPS compared to the results we computed.

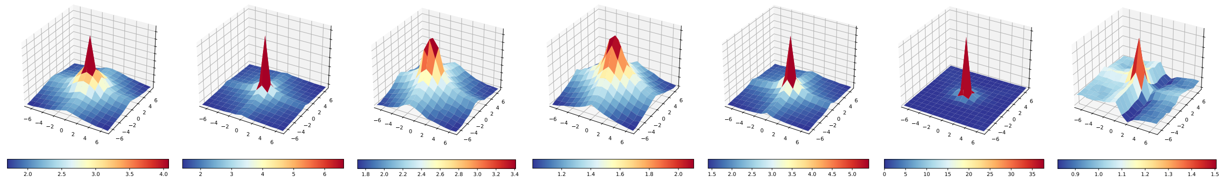

(a) HiCExplorer **(b)** HiCCUPS **(c)** HOMER **(d)** Chromosight **(e)** Cooltools **(f)** Fit-Hi-C **(g)** Peakachu

Figure 2. Aggregated loop locations of detected loops on GM12878, 10 kb resolution for the different detection algorithms. Aggregation is performed with HiCExplorer's hicAggregateContacts.

accuracy (CTCF ChIP-Seq 0.71; CTCF ChIA-PET 0.64), the expected value based on the non-zero interactions (Equation 5) has the highest number of detected loops (14144) and provides more absolute correlated loop locations (CTCF ChIP-Seq 9352; CTCF ChIA-PET 7808). Last, the correction for ligation events as proposed by the HOMER (Equation 6) software shows the lowest accuracy (CTCF ChIP-Seq 0.58; CTCF ChIA-PET 0.48). The results depend on the data: the fewer reads a Hi-C matrix has, the sparser it is, and the fitted distributions are more biased towards zero. In this case, interactions with a lower interaction count have a lower p-value and are more likely to be detected. However, excluding the zero contacts from the distribution can lead to a bias in the other direction; interaction values that should be detected have a p-value which is too high and are therefore excluded from the computation.

For other cell lines published by Rao 2014, the situation is comparable (Supplementary Table 1). The number of detected loops ranges between 3000 and 10000 loops. The non-zero values and implicitly the read coverage per bin help to explain this different detection behavior; the higher the read coverage, the more regions are detected (see Supplementary Tables 1, 4, and 5). The candidate selection approach via the definition of a neighborhood makes the algorithm sensitive to the Hi-C interaction matrix's resolution. The lower the resolution, the smaller the neighborhood needs to be. Otherwise, the chances of having elements in the neighborhood, peaks or TADs, or even the main diagonal, are too high. At the same time, decreasing the size of the neighborhood creates another issue: the number of elements in the peak and background regions becomes too low. This leads to non-significant test results and to the insight that firstly, the neighborhood size should be adjusted to the bin resolution of the Hi-C matrix, and secondly, that a neighborhood should contain at least around 250 - 300 elements to produce valuable results.

Comparison to state-of-the-art approaches

In the following section the detected loops by different tools on the Hi-C interaction matrices of the cell lines GM12878, HMEC, HUVEC, IMR90, K562, KBM7 and NHEK (by [8]) with the Knight-Ruiz correction [2] are compared. The search distance is restricted to 8 MB if the tool allows this; the results are post-processed for all others. The tools compared are: HiCExplorer, HiCCUPS, HOMER, chromosight, cooltools, Fit-Hi-C, and Peakachu.

Detected loop comparison

The detection rate is comparable for all tools and cell lines (Supplementary Table 1), except for chromosight and Peakachu. Chromosight detects significantly more loops with a very low p-value; however, as the loops' visualization (Figure 3c, chromosome 1 18.00 - 22.00 MB)) indicates, most detected loops are in very noisy regions, and it is questionable what exactly chromosight is detecting. This is supported by the analysis of additional regions, see Supplementary Figure 4c (chromo-

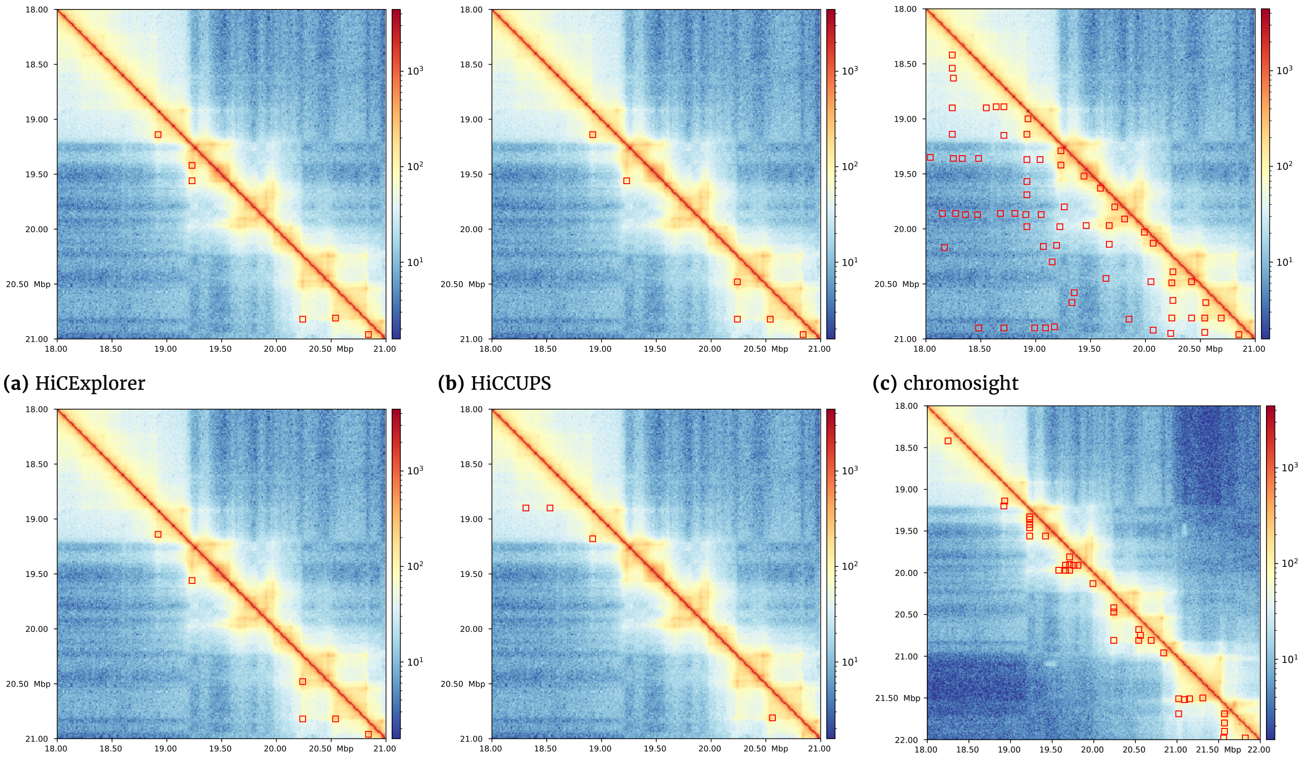

(a) HiCExplorer **(b)** HiCCUPS **(c)** chromosight

(d) cooltools **(e)** Peakachu **(f)** Peakachu (authors data)

Figure 3. Plot of chr1 18 - 22 Mb on GM12878, with the detected loops highlighted from each software. HiCExplorer, HiCCUPS, and cooltools show similar results. Chromosight detects many loops in noisy regions and lacks specificity. The four loops of Peakachu show a general issue of this algorithm: The first two loops (18 Mb region) are in a region without enrichment, and the two others slightly miss the enriched interactions by a few kilobases. HOMER and Fit-Hi-C do not detect any loop in the area. The subplot **(e)** is based on the authors computations and **(f)** is based on the loops as they have been published by the authors of Peakachu in [7]. Plots are produced using HiCExplorer hicPlotMatrix.

some 4 20.55 - 22.55 MB), 6c (chromosome 1 15.00 - 18.00 MB) and 8c (chromosome 10 90.00 - 92.00 MB). On the other hand, Peakachu detects much fewer loops than the other algorithms considered. After correspondence with the authors, it became clear that the models provided were trained on ICE-corrected matrices, whereas we have used Knight-Ruiz corrected matrices. For this reason, the loops detected by Peakachu, as published by the authors, have also been taken into consideration. Nonetheless, a detailed analysis of loop loci shows that Peakachu misses important loops, regardless of whether the KR data or the author's own results are considered. For example, the region chromosome 4 20.55 - 22.55 Mb contains four visible loops: Peakachu on KR detects two of them, and misses one completely. Additionally, two locations are detected that slightly miss a loop (Supplementary Figure 4e). The Peakachu results provided by the authors miss two loops and detect the two others successfully (Supplementary Figure 4f). Supplementary Figure 6e shows another issue of Peakachu on KR data. Many loops are detected at the border of a faulty region; it seems the machine learning approach did not have access to this kind of data in training. The data provided by the authors of Peakachu do not have this kind of issue, but overall, while the provided data contain more correct locations, the detection sometimes detects too many loops, for example, in the region chromosome 10 90 - 92 Mb (Supplementary Material 8f). The third problematic tool is Fit-Hi-C. The number of detected loops is at first sight comparable to the other tools; the loci-specific analysis cannot confirm this. The regions chromosome 1 15 - 22 MB (Figure 3 and Supplementary Figure 6h), chromosome 4 20.55 - 22.55 MB (Supplementary Figure 4h) or chromosome 10 90.00 - 92.00 MB have no loops detected by Fit-Hi-C, while the other tools are able to detect loops in these regions. In comparison, the regions where Fit-Hi-C does detect loops are eye-opening. The regions chromosome 1 13.00 - 14.00 MB (Supplementary Figure 5) and chromosome 1 142.00 - 144.00 MB contain mostly very sparse or even faulty Hi-C data. Fit-Hi-C detects an overwhelming amount of enriched pixels in these regions and returns these as loops. While it might be true that these pixels are enriched in a local context, they are far from being a loop. The pattern of the accumulated loop locations (Figure 2) confirms that the detected pattern is usually a single enriched interaction. The other tools detect only very few or no loops in the regions chromosome 1 13.00 - 14.00 MB and chromosome 1 142.00 - 144.00 Mb. Supplementary Figure 7 indicates HiCExplorer and HiCCUPS also have issues in noisy regions. An explanation is how loops are detected: Both tools detect first outliers and later consider the backgrounds with the loop regions based on statistical tests. Regions which are noisy, but to the statistical test show two different distributions do pass the criterion of detection. This behaviour is present also for the most of the other tools and is considered by us a weakness of statistical based approaches. The intersection between the detected peaks of HiCExplorer, HiCCUPS, HOMER, chromosight, cooltools, Fit-Hi-C, and Peakachu is quite different (Figure 1). HiCExplorer, with a search distance of 8 Mb, shares ~ 46% of its loops with HiCCUPS. HiCExplorer has the highest intersection of detected loops with chromosight, but chromosight also provides the highest number of detected loops. The intersection of detected loops with cooltools is similar to HiCCUPS; the number of intersecting loops with HOMER, Fit-Hi-C, and Peakachu is lower. HiCCUPS and cooltools show the highest intersecting numbers, chromosight profits from its high detection rate, while HOMER shares only a few hundred loops with HiCCUPS, similar to its intersection with HiCEx-

plorer. The intersection of Fit-Hi-C and Peakachu with HiCExplorer and HiCCUPS is very low, and the results of the Peakachu publication cannot be confirmed. Concerning Peakachu, it can be assumed that the performance is directly connected to the trained models and its inadequate generalization ability. In the publication describing Peakachu, the authors write they have used a probability threshold for a pixel between 90% and 97%. However, to detect a similar number of loops to have comparability, we had to use a score of 68%. For Fit-Hi-C, the authors of Peakachu have used a threshold of 10^{-5}, while we used 0.01 to enable detection of a few thousand loops.

Loop location correlation to protein locations

The detected loops are correlated with CTCF and cohesin factors (Supplementary Table 2) to investigate the amount of intersecting locations. This correlation is computed because it was shown that at the anchor points of loops, the proteins CTCF and cohesin are involved as loop binding factors [8, 15]. However, the loop structures representing gene or polycomb-mediated loops do not have CTCF at their anchor points, and the correlation can only be as good as the quality of the ChIP-Seq data from which it is derived. This measurement is, therefore, only an indicator of the accuracy of the detection.

The number of loops detected by HiCExplorer are comparable to HiCCUPS. On the GM12878 cell line and correlated to ChIA-PET based CTCF locations, HiCExplorer detects a similar amount of loops compared to HiCCUPS (6540 vs. 6564) but is more specific (0.64 vs. 0.61). Cooltools (5467 loops) and the loops provided by the Peakachu authors (8174 loops) have a similar relative value of 54% and 50%. Based on our computations, the loops detected with Peakachu have a match at only 686 loop locations and a relative value of 5%. The correlation for the other three tools is also low. Chromosight has 7205 loops correlated, a share of only 11%, Homer has 1349 loops and a share of 18%, and last, Fit-Hi-C has only 163 correlated loop locations with a share of 2%.

The correlation of locations for ChIA-PET RAD21, a cohesin subfactor, has overall significantly lower correlations. HiCExplorer has 2577 loops (25%), HiCCUPS 2385 loops (22%), cooltools 1781 loops (17%), and the loop locations provided by the Peakachu authors 2554 (15%). All other tools have a meager share of correlated locations of < 3%. As a second source of information, data from HiChIP experiments is also considered. The correlation values are overall much higher: for the histone H3K27ac, the highest correlation is achieved by the author-provided Peakachu results with 96%, followed by HiCCUPS with 92%, cooltools with 85% and HiCExplorer reaching only fourth place with 86%. The results of the other tools are also much higher than the results of CTCF and RAD21; for example, Fit-Hi-C had only 2% matches with RAD21, but has 29% with H3K27ac. The correlation based on SMC1, a cohesin subfactor, created with HiChIP indicates the same: again, the author-provided Peakachu results are the highest with 99% followed by HiCCUPS (96%), cooltools (94%), HiCExplorer (91%) and Homer (90%). The correlation of the low performing Fit-Hi-C 2 is 34% higher compared to other proteins, but is also low compared to all other tools. Last, the proportions of the detected locations of the different tools were tested for significant differences. A two-sided proportion z-score test was used, and given the H0 'the proportion is equal', the H0 was rejected for all datasets and tools, under a p-value of 0.05 (Supplementary Table 3).

Non-intersected loops

The two previous sections investigated the intersection of loops between different tools and their correlation to structural proteins. The intersection of all detected loops between HiCExplorer and HiCCUPS is 46%, and both tools have a high correlation to structural proteins for their detected loops. However, the non-intersecting detections have not been investigated. The above-discussed correlation to structural protein locations indicates that the loops detected by either HiCExplorer or HiCCUPS have a high match to the positions of structural proteins. The situation is similar for the unique detect loops of either HiCExplorer or HiCCUPS. The correlation of unique loops to ChIA-PET based CTCF locations shows a lower matching than all detected locations, 0.49 to 0.64 for HiCExplorer and 0.46 to 0.61 for HiCCUPS. A similar pattern is present for the other proteins: ChIA-PET RAD21 0.15 to 0.25 for HiCExplorer and 0.11 to 0.22 for HiCCUPS; HiChIP H3K27ac 0.78 to 0.86 and 0.89 to 0.92; HiChIP SMC1 0.85 to 0.91 and 0.93 to 0.96. The lower correlations for the uniquely detect loops of HiCExplorer and HiCCUPS indicate a higher false detection if a loop is not detected by both tools; however, the correlations are still on a high level. The unique detect loops are in their large majority of high value for the investigation of DNA loop structures.

Averaged loop structure comparison

The accumulated contacts of all detected loop locations on GM12878, displayed as a 3D plot in Figure 2, shows that all algorithms detect enriched regions, but the neighborhood structure is very different. HiCExplorer detects a sharp peak with an enriched direct neighborhood, while HiCCUPS and Fit-Hi-C have a very sharp peak with almost no neighborhood signal. HOMER and Chromosight detect broader peaks with a highly enriched neighborhood, and cooltools has a sharp peak and a neighborhood structure that is slightly more enriched than HiCCUPS and slightly less than HiCExplorer. Finally, Peakachu detects a sharp peak and has a neighborhood plateau on one side, similar to the other algorithms, but a sharp cliff on the other side. The visualizations indicate that a broad peak detection as provided by HOMER and Chromosight, or a very sharp peak with no neighborhood signal, have a low correlation to CTCF-based loops. The visualization of Peakachu's loop locations with the sharp cliff can be interpreted as locations with a TAD border. This can be explained in the context of a learned model based on CTCF locations, because CTCF is present at both loop locations and TAD boundaries.

Runtime and memory usage

The runtime and memory performance is a crucial factor in determining the quality of an algorithm, as well as its implementation. The performance was measured on the Hi-C interaction matrices of the cell lines by Rao [8] discussed above, with a 10 kb resolution for the tools HiCExplorer, HiCCUPS, Homer, chromosight, cooltools, Fit-Hi-C 2, and Peakachu. The measures was computed on an AMD 3700X with 128 GB memory and an Nvidia GTX 1070. For a fair comparison, the CPU implementations are considered, but for completeness, it should be mentioned that the GPU implementation of HiCCUPS with the search space restriction mode of 8 MB active was over all datasets the fastest approach.

On the 8 Mb search distance range, HiCExplorer is the fastest CPU implementation, except for GM12878 cell lines where the CPU-based version of HiCCUPS is faster. HiCExplorer is ~ 44% faster than chromosight (4:25 min vs. 6:22 min) on GM12878 with a 8 Mb search distance, and uses only 6.7 GB memory, while chromosight consumes 39 GB. Moreover, HiCExplorer is two times faster than cooltools if only loop detection is considered; if the necessary computation of expected values is added, it is almost 3.5 times faster (Supplementary Table 8). When considering the somewhat theoretical measure of single-core performance, Chromosight is the fastest algorithm (Supplementary Table 9); nonetheless, modern CPUs support up to 64 cores / 128 threads, and data analysis software should use the offered resources as well as possible. For this reason, HiC-

Explorers' hicDetectLoop supports parallelization by chromosomes as well as intra-chromosomal parallelization. The data structure allows this: each chromosome can be computed independently, as can each genomic distance normalization, distribution fitting, and p-value computation. For example, if 23 threads are used to compute each chromosome in parallel, and for each chromosome thread, ten other threads compute all intra-chromosomal computations in parallel, a total of 230 parallel threads are used. Not all threads are used at the same time; therefore, a good utilization is achieved. However, modern CPUs with core/thread counts of 64 / 128 can be fully utilized with this approach. Two of the algorithms, Fit-Hi-C and Peakachu, provide only a single-core implementation. Their runtimes are by far the slowest, taking 4:46 hours and 7:03 hours on the GM12878 data, and consume at the same time a high amount of memory. HOMER is, in all scenarios, the third slowest algorithm and also consumes the most memory. However, HOMER is also the only algorithm without any search space restriction parameter, so that all searches are performed genome-wide. This has the side effect that, for example, the GM12878 dataset could only be computed using a single core, because the memory consumption was already around 100 GB. The approach chosen by the developers of HOMER to not support any binary file format to store and access the Hi-C interaction matrix, such as Juicer's *hic* or the *cooler* [4] file format supported by many of the other investigated tools, results in a computation based on text files and raw data, and contributes, apart from the lack of a search space restriction, to the very poor runtime and memory performance.

Discussion

The search space of an algorithm is the dominant factor determining its accuracy and performance. Therefore, pruning it should be the primary goal when optimizing newly designed algorithms. In theory, brute force solutions which apply no restrictions to the search space, like HiCCUPS, can detect all possible enriched regions, but the result is an implementation with very demanding hardware requirements. HiCCUPS solves this by utilizing massively parallel computational resources via GPGPU. On the other hand, HOMER also applies no limitations to the search space, yet detects a lower number of loops, and those which are detected have a significantly lower correlation over all samples to CTCF localization. HOMER does support a parallel computation per chromosome, like HiCExplorer but is significantly slower than all other solutions and uses significantly more memory per core. HOMER's poor runtime performance can be explained by the fact that computation is performed on raw data, while all other approaches use precomputed interaction matrices. Chromosight is a fast detection approach and provides the fastest single-core performance; however, it lacks specificity and detects many loops that should be considered noise, even though these loops may be provided with a high significance. Cooltools, with its reimplementation of the HiCCUPS approach, provides a genome distance search which makes it faster and more flexible. The results are good, but it is unclear why they are not more similar to Juicer's HiCCUPS results, given that the same algorithm is used. An overview of the properties of all algorithms is provided in Supplementary Table 10.

The divergence between the Peakachu results based on our computations and the data published by the authors is high. Given that the machine learning-based model of Peakachu is trained using the locations of certain proteins, it is unsurprising that the H3K27ac and SMC1 locations have very high correlation values. Our understanding is that the published trained model does not detect loop locations themselves, but rather the locations of SMC1 and H3K27ac. Moreover, the poor performance on the KR corrected matrix used indicates heavy overfitting and a poor generalization ability onto different kinds of input matrices. Another explanation could be the lack of preprocessing to normalize the input data. The idea of training a model using the locations of proteins known to be correlated with loops is sensible, but is limited by the fact that not all loop locations have CTCF and cohesin at their anchors. Overall, the model is an interesting approach; nonetheless, the published model requires a more diverse training data to improve performance on varying input datasets.

Furthermore, it could be shown that the sparsity and thus the read coverage of a Hi-C interaction matrix significantly influences the detection of peaks in their neighborhood. The sparser a Hi-C interaction matrix is, the more likely that the possible valid regions detected by the continuous negative binomial distribution filtering are rejected by the Wilcoxon rank-sum test. The large number of differences between the detected loops and the high correlation rates to CTCF can be explained in multiple ways. The correlation to CTCF has its roots in biology. Not all loops have CTCF as a binding protein at its anchors; gene-loops or polycomb-mediated loops lack it. All the algorithms detect enrichments in the Hi-C data, which are interpreted as loops, but may also have other explanations. The enrichments can also be noise in the data, or interactions which are unrelated to CTCF. Secondly, the Hi-C data is created with in-situ Hi-C and has a higher noise level than newer approaches like Arima Hi-C[3]. Detections of loops in noisy areas is responsible for the low intersection values for the predictions of the competing algorithms, in particular for chromosight, which detects more noise than loops.

Availability of source code and requirements

Project name: HiCExplorer
Project home page: https://github.com/deeptools/HiCExplorer/
Operating system(s): Linux / MacOS
Programming language: Python
Other requirements: Python 3.6 and higher
License: GPLv3
RRID: SCR_022111
biotools ID: https://bio.tools/hicexplorer

Availability of supporting data and materials

The following identifier are NCBI GEO accession numbers.

Hi-C data: GSE63525; Rao et al. [8]. CTCF for: Gm12878 from GSM935611; Hmec from GSM749753; Huvec from GSM749749; K562 from GSM733719 and Nhek from GSM733636. CTCF ChIA-PET (GSM1872886); H3K27ac HiChIP (GSE101498), SMC1 HiChIP (GSE80820), and RAD21 ChIA-PET (GSM1436265). Result files are available via Zenodo [16].

Declarations

Competing Interests

The author(s) declare that they have no competing interests.

3 https://arimagenomics.com/

Funding

German Federal Ministry of Education and Research [031 A538A de.NBI-RBC awarded to R.B.]; German Federal Ministry of Education and Research [031 L0101C de.NBI-epi awarded to B.G.]. R.B. was supported by the German Research Foundation (DFG) under Germany's Excellence Strategy (CIBSS - EXC-2189 - Project ID 390939984).

Author's Contributions

JW: Designed and implemented the presented algorithm and wrote the manuscript. RB: contributed to the manuscript. BG: contributed to the manuscript.

Acknowledgements

We thank Simon Bray and Anup Kumar for proofreading the manuscript.

References

1. Imakaev M, Fudenberg G, McCord RP, Naumova N, Goloborodko A, Lajoie BR, et al. Iterative correction of Hi-C data reveals hallmarks of chromosome organization. Nature Methods 2012 sep;9(10):999–1003. http://www.nature.com/doifinder/10.1038/nmeth.2148, [PubMed:22941365] [PubMed Central:PMC3816492] [doi:10.1038/nmeth.2148].
2. Knight PA, Ruiz D. A fast algorithm for matrix balancing. IMA Journal of Numerical Analysis 2013;33(3):1029–1047. [doi:10.1093/imanum/drs019].
3. Heinz S, Benner C, Spann N, Bertolino E, Lin YC, Laslo P, et al. Simple Combinations of Lineage-Determining Transcription Factors Prime cis-Regulatory Elements Required for Macrophage and B Cell Identities. Molecular Cell 2010 may;38(4):576–589. https://www.sciencedirect.com/science/article/pii/S1097276510003667?via{%}3Dihub, [PubMed:20513432] [PubMed Central:PMC2898526] [doi:10.1016/j.molcel.2010.05.004].
4. Abdennur N, Mirny LA. Cooler: scalable storage for Hi-C data and other genomically labeled arrays. Bioinformatics 2020;36(1):311–316.
5. Matthey-Doret C, Baudry L, Breuer A, Montagne R, Guiglielmoni N, Scolari V, et al. Computer vision for pattern detection in chromosome contact maps. Nature communications 2020;11(1):1–11.
6. Kaul A, Bhattacharyya S, Ay F. Identifying statistically significant chromatin contacts from Hi-C data with FitHiC2. Nature protocols 2020;15(3):991–1012.
7. Salameh TJ, Wang X, Song F, Zhang B, Wright SM, Khunsriraksakul C, et al. A supervised learning framework for chromatin loop detection in genome-wide contact maps. Nature communications 2020;11(1):1–12.
8. Rao SSP, Huntley MH, Durand NC, Stamenova EK. A 3D Map of the Human Genome at Kilobase Resolution Reveals Principles of Chromatin Looping. Cell 2014;159(7):1665–1680. http://dx.doi.org/10.1016/j.cell.2014.11.021, [PubMed:25497547] [PubMed Central:PMC5635824] [doi:10.1016/j.cell.2014.11.021].
9. Wolff J, Backofen R, Gruening B. Loop detection using Hi-C data with HiCExplorer. bioRxiv 2020;.
10. Wolff J. Approaches to analysis of chromosome conformation capture data. freiDok 2020;https://doi.org/10.6094/UNIFR/224705.
11. Cameron AC, Trivedi PK. Regression-based tests for overdispersion in the Poisson model. Journal of econometrics 1990;46(3):347–364.
12. Robinson MD, McCarthy DJ, Smyth GK. edgeR: a Bioconductor package for differential expression analysis of digital gene expression data. Bioinformatics 2010;26(1):139–140.
13. McCarthy DJ, Chen Y, Smyth GK. Differential expression analysis of multifactor RNA-Seq experiments with respect to biological variation. Nucleic acids research 2012;40(10):4288–4297.
14. Andrey G, Schöpflin R, Jerković I, Heinrich V, Ibrahim DM, Paliou C, et al. Characterization of hundreds of regulatory landscapes in developing limbs reveals two regimes of chromatin folding. Genome research 2017;27(2):223–233. [PubMed:27923844] [PubMed Central:PMC5287228] [doi:10.1101/gr.213066.116].
15. Bonev B, Cavalli G. Organization and function of the 3D genome. Nature Reviews Genetics 2016;17(11):661. [PubMed:28704353] [doi:10.1038/nrg.2016.147].
16. Wolff J, Loop detection using Hi-C data with HiCExplorer. Zenodo; 2021. https://doi.org/10.5281/zenodo.5648500.

Click here to access/download
Supplementary Material
supplement_R3.pdf
