# Supplementary material for: Loop detection using Hi-C data with HiCExplorer
Source: Gigascience. 2022 Jul 9;11:giac061. doi: 10.1093/gigascience/giac061 (PMC9270730; doi:10.1093/gigascience/giac061)
Supplement: giac061_Supplemental_Files [file giac061_supplemental_files.zip › loop_final_supplementary.pdf]

Supplementary material: Loop detection using Hi-C data with HiCExplorer

Joachim Wolff [1,2*], Rolf Backofen [2,3], Björn Grüning [2]

[1]Friedrich Miescher Institut for Biomedical Research, Maulbeerstrasse 66, 4058 Basel, Switzerland
[2] Bioinformatics Group, Department of Computer Science, University of Freiburg, Georges-Köhler-Allee 106, 79110 Freiburg, Germany
[3]Signalling Research Centres CIBSS, University of Freiburg, Schänzlestr. 18, 79104 Freiburg, Germany

[*]To whom correspondence should be addressed.

1 Density distributions for genomic distances

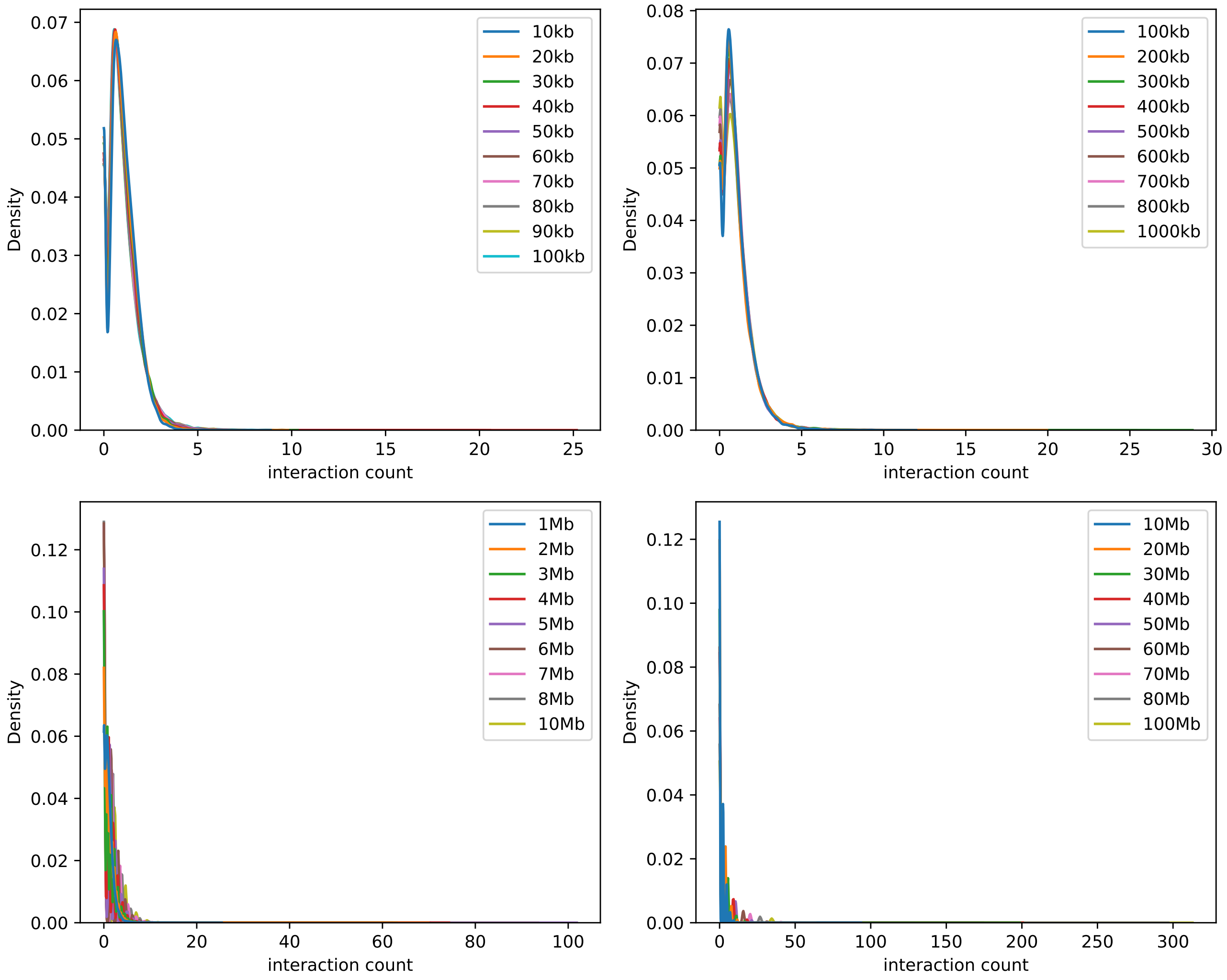

Figure S 1: Value density distributions per genomic distances on Gm12878. Values are observed / expected normalized per genomic distance.

2 Overdispersion test

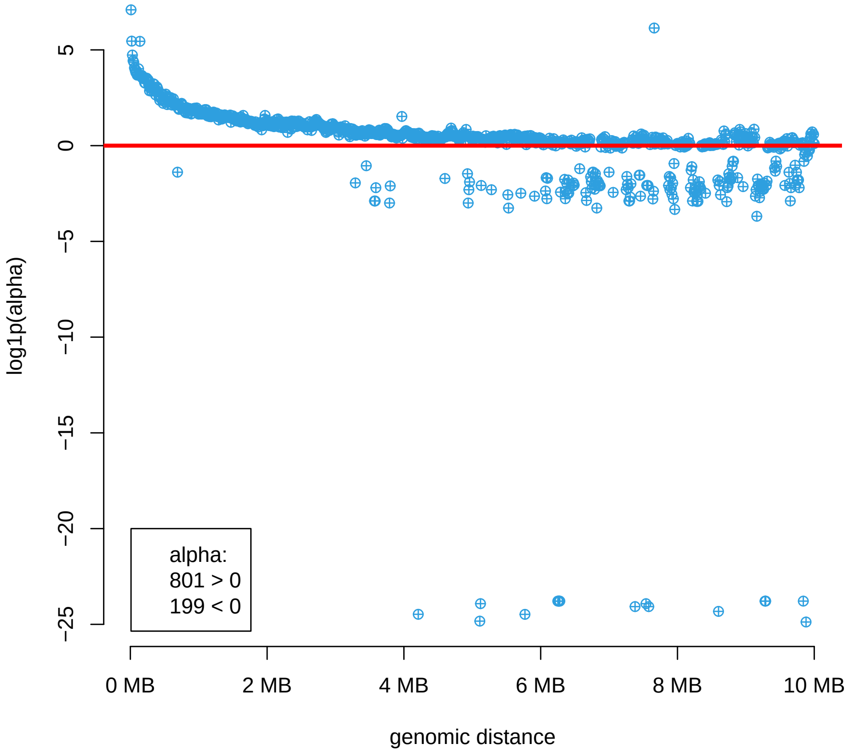

Figure S 2: Overdispersion test from Cameron & Trivedi 1990. Tested on the raw data of chromosome 1 of GM12878 cells, 10 kb resolution. The mayority of the distances (80.1%) has an overdispersion.

3 Detected loops and accuracy

Data	HiCExplorer	HiCCUPS	HOMER	chromosight	cooltools	Fit-Hi-C	Peakachu	Peakachu (p.b.a.)
GM12878	10225	10603	7182	60789	9987	7784	12279	16237
HMEC	5705	7424	7152	20160	5259	7784	12279	-
HUVEC	3489	2119	4052	16621	1869	1118	885	-
IMR90	8205	10255	9556	34572	8582	2487	484	-
K562	5838	5093	6968	25811	4527	- (*)	- (*)	-
KBM7	4101	1924	3170	21341	1869	2182	1625	-
NHEK	5211	2937	5409	25770	2662	1720	1042	-

Table 1: Detected loops on different cell types cells from Rao 2014, with 10kb resolution, and a 8 MB search restriction. If the tool was not able to provide a search space restriciton, the data has been post-processed to fulfill the requirement. Peakachu (p.b.a.): **p**ublished **b**y **a**uthors) uses precomputed loops published by the authors of Peakachu; data was only provided for GM12878 cell line and not for the others. (*) The computation crashed for Fit-Hi-C and peakachu on K562 data set.

Data	CTCF ChIA-PET	H3K27ac HiChIP	RAD21 ChIA-PET	SMC1 HiChIP
HiCExplorer	6540 (0.64)	8835 (0.86)	2577 (0.25)	9346 (0.91)
HiCCUPS	6564 (0.61)	9831 (0.92)	2385 (0.22)	10179 (0.96)
cooltools	5467 (0.54)	8857 (0.88)	1781 (0.17)	9396 (0.94)
chromosight	7205 (0.11)	41599 (0.68)	1785 (0.02)	47056 (0.77)
Homer	1349 (0.18)	5368 (0.74)	286 (0.03)	6470 (0.90)
FitHi-C 2	163 (0.02)	2279 (0.29)	109 (0.01)	2656 (0.34)
Peakachu	686 (0.05)	4873 (0.39)	78 (0.006)	6150 (0.50)
Peakachu (p.b.a.)	8174 (0.50)	15688 (0.96)	2554 (0.15)	16207 (0.99)

Table 2: Correlation of detected loops of the GM12878 cell line on 10 kb resolution and 8 Mb genomic distance restriction with various HiChIP and ChiA-PET: CTCF ChIA-PET (GSM1872886); H3K27ac HiChIP (GSE101498), SMC1 HiChIP (GSE80820), and RAD21 ChIA-PET (GSM1436265) data. The data for Peakachu is measured twice: First, the trained model as published by the authors of Peakachu is used to detect loops on a KR corrected matrix as it was used for all other tools. The second data (Peakachu **p**ublished **b**y **a**uthors) uses precomputed loops published by the authors of Peakachu.

	HiCExplorer	HiCCUPS	Homer	chromosight	cooltools	FitHi-C 2	Peakachu	Peakachu (p.b.a.)
HiCExplorer	1.00e+00	2.16e-03	0.00e+00	0.00e+00	1.27e-40	0.00e+00	0.00e+00	1.59e-104
HiCCUPS		1.00e+00	0.00e+00	0.00e+00	1.86e-25	0.00e+00	0.00e+00	2.43e-77
Homer			1.00e+00	5.99e-63	0.00e+00	4.17e-251	2.22e-184	0.00e+00
chromosight				1.00e+00	0.00e+00	4.99e-151	8.39e-92	0.00e+00
cooltools					1.00e+00	0.00e+00	0.00e+00	4.37e-12
FitHi-C 2						1.00e+00	2.22e-33	0.00e+00
Peakachu							1.00e+00	0.00e+00
Peakachu (p.b.a.)								1.00e+00

(a) P-values of two-sided proportion z-test based on CTCF ChIA-PET intersection locations and overall number of detected loops.

	HiCExplorer	HiCCUPS	Homer	chromosight	cooltools	FitHi-C 2	Peakachu	Peakachu (p.b.a.)
HiCExplorer	1.00e+00	1.99e-50	4.27e-85	9.84e-301	9.37e-07	0.00e+00	0.00e+00	1.39e-211
HiCCUPS		1.00e+00	3.79e-244	0.00e+00	1.68e-23	0.00e+00	0.00e+00	2.97e-47
Homer			1.00e+00	7.01e-28	2.44e-126	0.00e+00	0.00e+00	0.00e+00
chromosight				1.00e+00	0.00e+00	0.00e+00	0.00e+00	0.00e+00
cooltools					1.00e+00	0.00e+00	0.00e+00	2.91e-143
FitHi-C 2						1.00e+00	7.54e-51	0.00e+00
Peakachu							1.00e+00	0.00e+00
Peakachu (p.b.a.)								1.00e+00

(b) P-values of two-sided proportion z-test based on H3K27ac HiChIP intersection locations and overall number of detected loops.

	HiCExplorer	HiCCUPS	Homer	chromosight	cooltools	FitHi-C 2	Peakachu	Peakachu (p.b.a.)
HiCExplorer	1.00e+00	4.47e-06	1.37e-302	0.00e+00	3.65e-37	0.00e+00	0.00e+00	2.50e-80
HiCCUPS		1.00e+00	5.41e-252	0.00e+00	8.85e-17	0.00e+00	0.00e+00	2.05e-44
Homer			1.00e+00	1.08e-06	1.30e-166	7.28e-23	4.20e-62	2.95e-142
chromosight				1.00e+00	0.00e+00	6.91e-15	2.85e-49	0.00e+00
cooltools					1.00e+00	2.89e-272	0.00e+00	8.45e-06
FitHi-C 2						1.00e+00	3.90e-08	2.44e-240
Peakachu							1.00e+00	0.00e+00
Peakachu (p.b.a.)								1.00e+00

(c) P-values of two-sided proportion z-test based on RAD21 ChIA-PET intersection locations and overall number of detected loops.

	HiCExplorer	HiCCUPS	Homer	chromosight	cooltools	FitHi-C 2	Peakachu	Peakachu (p.b.a.)
HiCExplorer	1.00e+00	1.05e-42	2.99e-03	4.12e-230	2.26e-13	0.00e+00	0.00e+00	5.28e-293
HiCCUPS		1.00e+00	2.00e-56	0.00e+00	2.07e-10	0.00e+00	0.00e+00	4.74e-124
Homer			1.00e+00	3.61e-136	1.79e-22	0.00e+00	0.00e+00	0.00e+00
chromosight				1.00e+00	0.00e+00	0.00e+00	0.00e+00	0.00e+00
cooltools					1.00e+00	0.00e+00	0.00e+00	3.53e-193
FitHi-C 2						1.00e+00	3.15e-109	0.00e+00
Peakachu							1.00e+00	0.00e+00
Peakachu (p.b.a.)								1.00e+00

(d) P-values of two-sided proportion z-test based on SMC1 HiChIP intersection locations and overall number of detected loops.

Table 3: Two-sided z-test for the different CTCF, H3K27ac, RAD21 and SMC1 propotions as shown in Table 2

Data	Initial candidates	Candidates for peak detection
GM12878	61.8 mio	1722
K562	19.2 mio	2948
KBM7	14.2 mio	2321
IMR90	19.3 mio	2948
NHEK	10.1 mio	2384
HUVEC	7.6 mio	3249

Table 4: Initial possible candidates vs. reduced candidate set of HiCExplorer for chromosome 1.

Data	Non-zero elements	Sparsity
GM12878	1,810 mio	0.0189
K562	781 mio	0.0081
KBM7	465 mio	0.0048
IMR90	415 mio	0.0043
NHEK	348 mio	0.0036
HUVEC	268 mio	0.0028
HMEC	188 mio	0.0019

Table 5: Sparsity level of the 10 kb Hi-C interaction matrices. The dense matrix contains 309,581 x 309,581 elements.

Data	Mean	Non-zero mean	Non-zero mean ligation
Loops	10255	14144	11566
CTCF ChIP-Seq	7298 (0.71)	9352 (0.66)	6808 (0.58)
CTCF ChIA-PET	6540 (0.64)	7808 (0.55)	5584 (0.48)
RAD21 ChIA-PET	2577 (0.25)	2907 (0.20)	2239 (0.19)
H3K27ac HiChIP	8835 (0.86)	11851 (0.83)	9271 (0.80)
SMC1 HiChIP	9346 (0.91)	12796 (0.89)	9991 (0.86)

Table 6: Different expected value computation for GM12878.

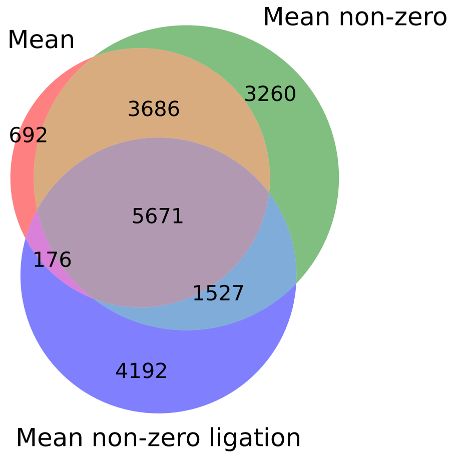

Figure S 3: Venn diagram with loop overlaps of the different expect value computations on GM12878.

Parameter	Loops	CTCF ChIA-PET accuracy
peakWidth=4, windowSize=5	2380	1803 (0.75)
peakWidth=2, windowSize=5	9147	6464 (0.70)
peakWidth=4, windowSize=7	7259	5034 (0.69)
pValuePreselection 0.01	4289	3416 (0.79)
pValuePreselection 0.05	8456	6072 (0.71)
pValuePreselection 0.2	9159	6468 (0.70)
pValue 0.01	4937	3927 (0.79)
pValue 0.1	11939	7476 (0.62)
peakInteractionsThreshold 1	9147	6464 (0.70)
peakInteractionsThreshold 20	9112	6464 (0.70)
peakInteractionsThreshold 50	8422	6294 (0.74)
obsExpThreshold 0.5	12331	7340 (0.59)
obsExpThreshold 1	12008	7334 (0.61)
obsExpThreshold 2	6099	4786 (0.78)

Table 7: The effect of different parameter settings on the results with HiCExplorer. All parameters use their default values (peakWidth 2, windowSize 5, pValuePreselection 0.1, pValue 0.025, peakInteractionThreshold 10, obsExpTheshold 1.5), if it is not specified otherwise.

4 Comparison of detected loops at different locations

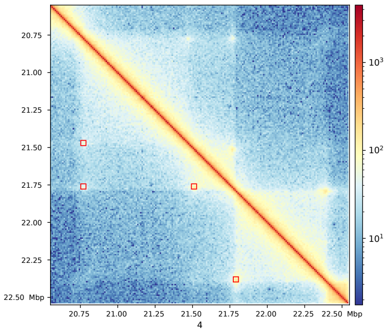

(a) HiCExplorer

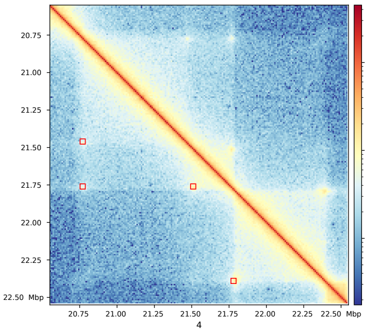

(b) HiCCUPS

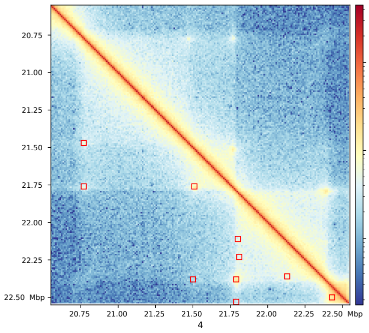

(c) chromosight

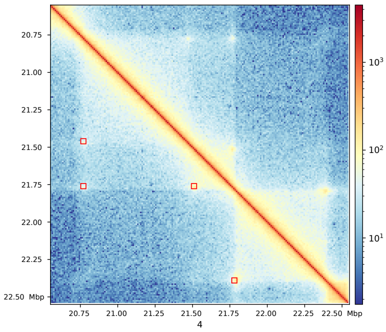

(d) cooltools

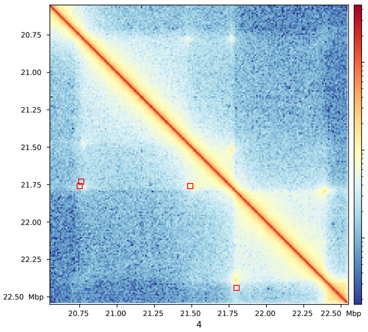

(e) Peakachu

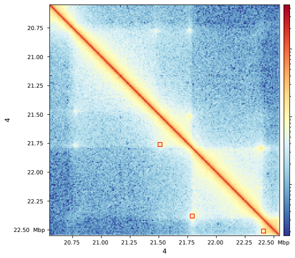

(f) Peakachu (p.b.a.)

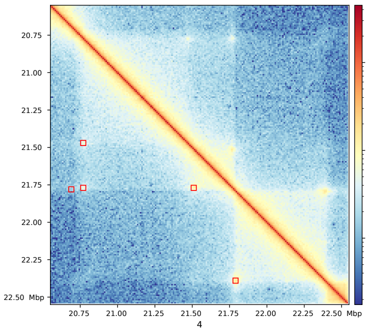

(g) Homer

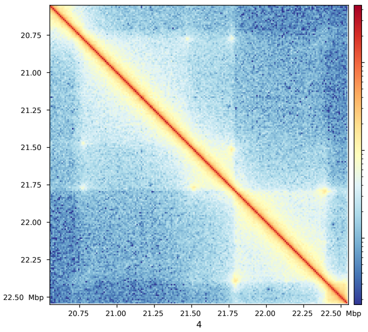

(h) FitHi-C 2

Figure S 4: The plot of chromosome 4 20.55 - 22.55 Mb on GM12878 and highlighted the detected loops from each software. HiCExplorer, HiCCUPS, and cooltools show similar results. Chromosight detects many loops in noisy regions and lacks specificity. The four loops of Peakachu show a general issue of this algorithm: The first two loops (18 Mb region) are in a region without enrichment, and the two others slightly miss the enriched interactions by a few kilobases. HOMER and Fit-Hi-C are not detecting any loop in the area. Plot with HiCExplorer hicPlotMatrix.

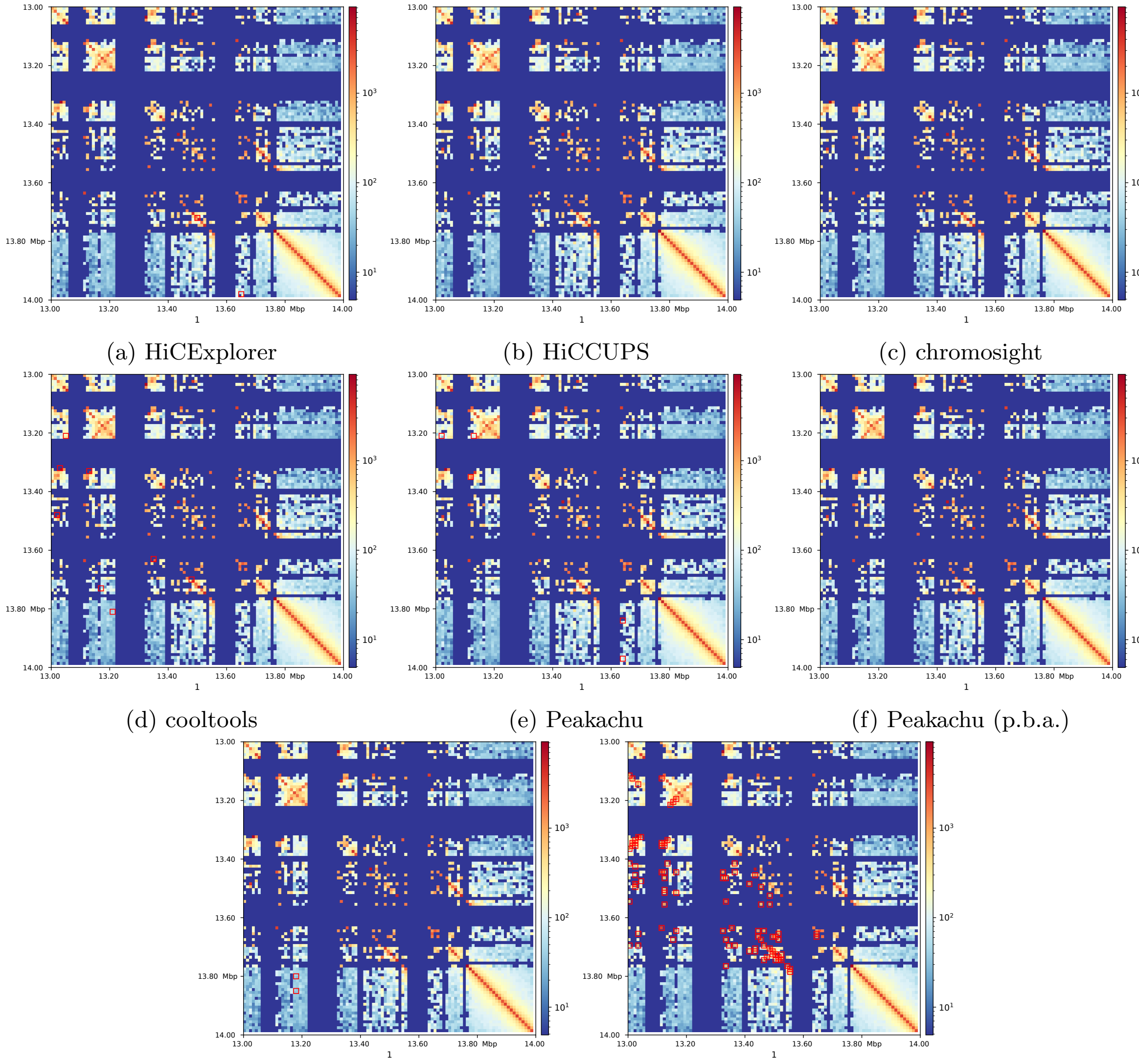

(a) HiCExplorer (b) HiCCUPS (c) chromosight

(d) cooltools (e) Peakachu (f) Peakachu (p.b.a.)

(g) Homer (h) FitHi-C 2

Figure S 5: The plot of chromosome 1 13.00 - 14.00 Mb on GM12878 and highlighted the detected loops from each software. HiCExplorer, HiCCUPS, and cooltools show similar results. Chromosight detects many loops in noisy regions and lacks specificity. The four loops of Peakachu show a general issue of this algorithm: The first two loops (18 Mb region) are in a region without enrichment, and the two others slightly miss the enriched interactions by a few kilobases. HOMER and Fit-Hi-C are not detecting any loop in the area. Plot with HiCExplorer hicPlotMatrix.

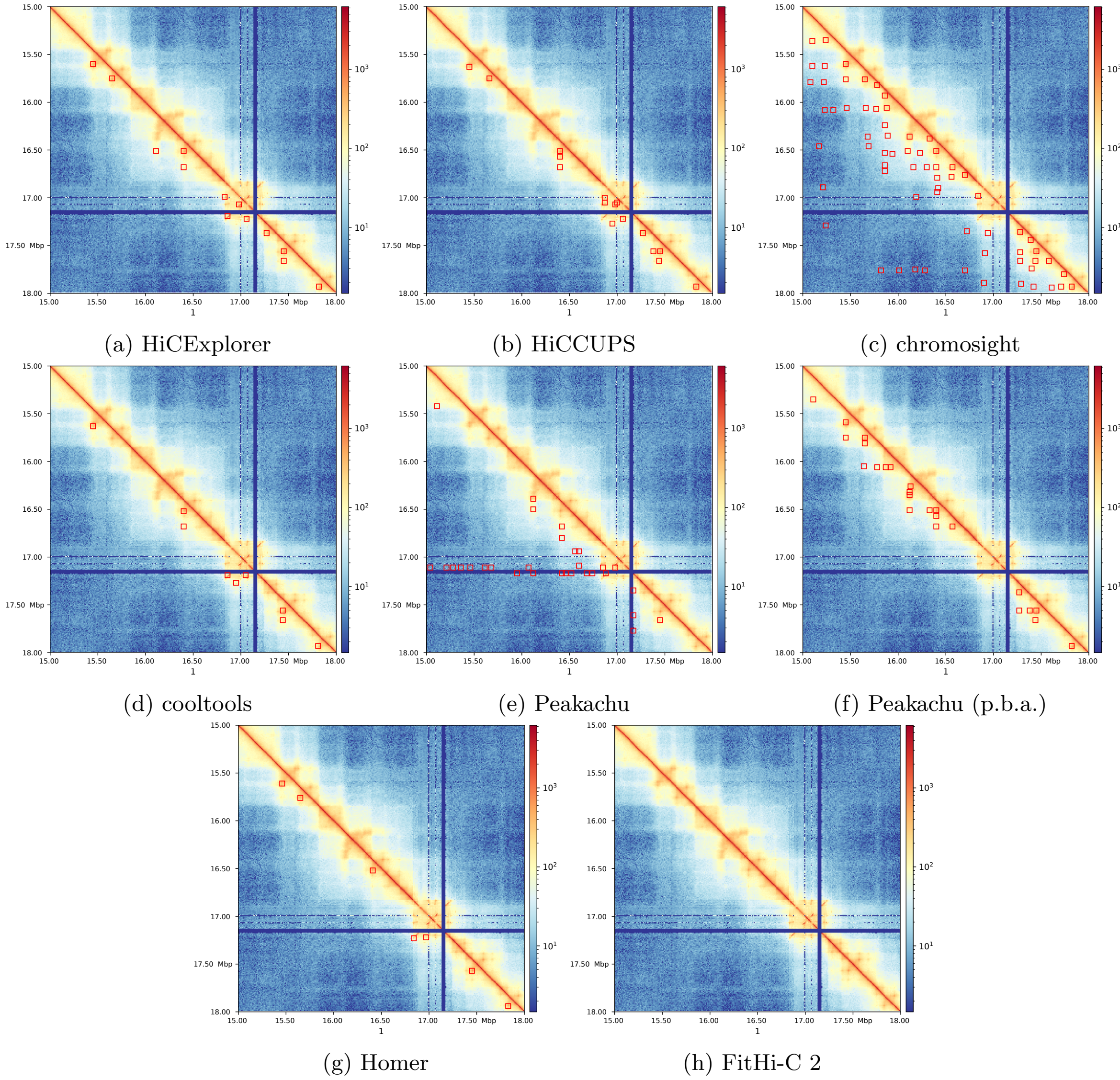

(a) HiCExplorer (b) HiCCUPS (c) chromosight

(d) cooltools (e) Peakachu (f) Peakachu (p.b.a.)

(g) Homer (h) FitHi-C 2

Figure S 6: The plot of chromosome 1 15.00 - 18.00 Mb on GM12878 and highlighted the detected loops from each software. HiCExplorer, HiCCUPS, and cooltools show similar results. Chromosight detects many loops in noisy regions and lacks specificity. The four loops of Peakachu show a general issue of this algorithm: The first two loops (18 Mb region) are in a region without enrichment, and the two others slightly miss the enriched interactions by a few kilobases. HOMER and Fit-Hi-C are not detecting any loop in the area. Plot with HiCExplorer hicPlotMatrix.

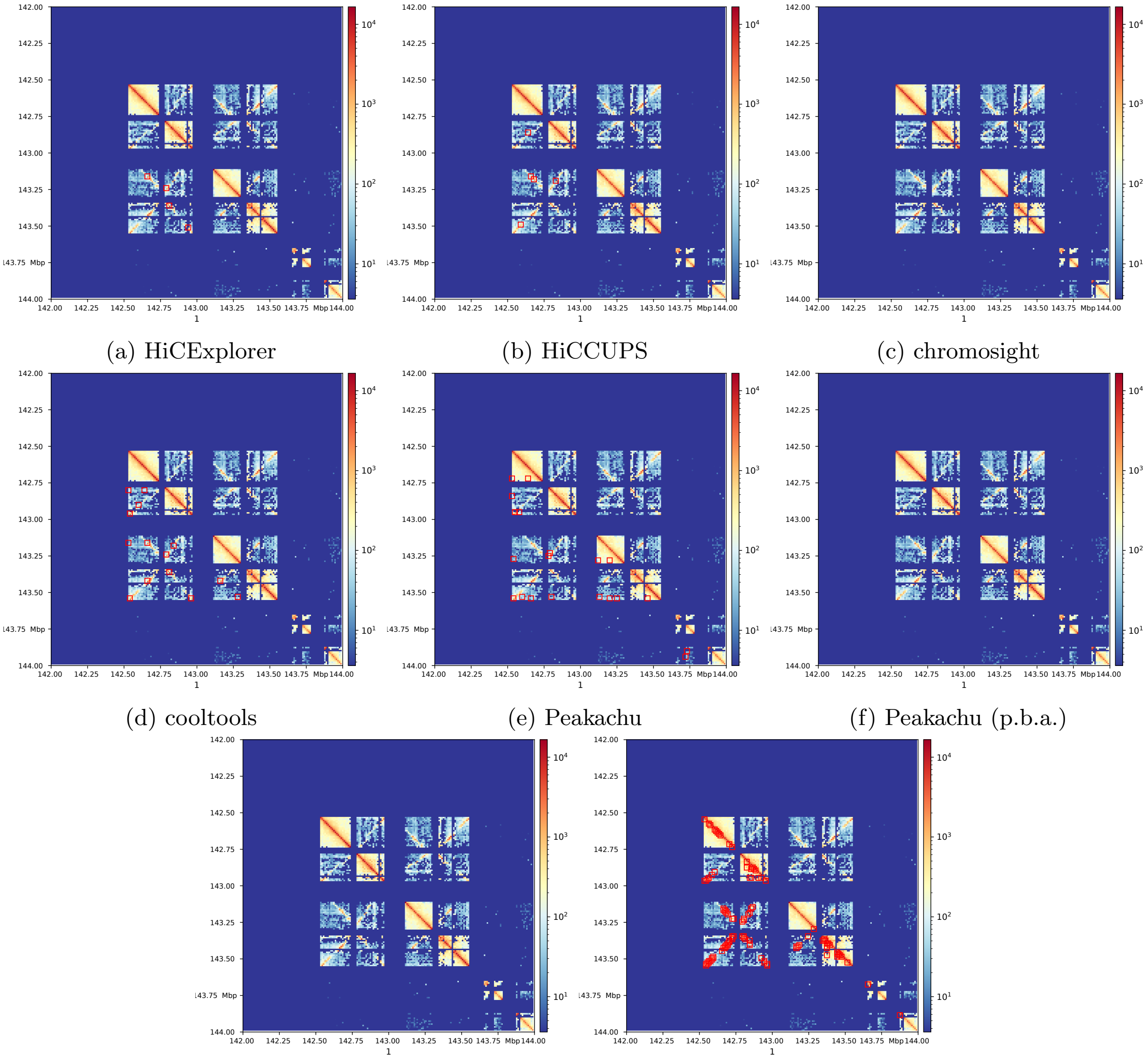

(a) HiCExplorer (b) HiCCUPS (c) chromosight

(d) cooltools (e) Peakachu (f) Peakachu (p.b.a.)

(g) Homer (h) FitHi-C 2

Figure S 7: The plot of chromosome 1 142.00 - 144.00 Mb on GM12878 and highlighted the detected loops from each software. HiCExplorer, HiCCUPS, and cooltools show similar results. Chromosight detects many loops in noisy regions and lacks specificity. The four loops of Peakachu show a general issue of this algorithm: The first two loops (18 Mb region) are in a region without enrichment, and the two others slightly miss the enriched interactions by a few kilobases. HOMER and Fit-Hi-C are not detecting any loop in the area. Plot with HiCExplorer hicPlotMatrix.

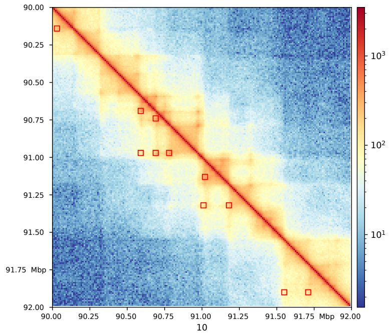

(a) HiCExplorer

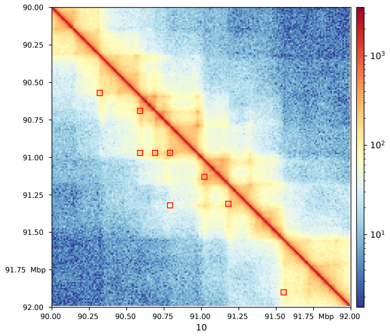

(b) HiCCUPS

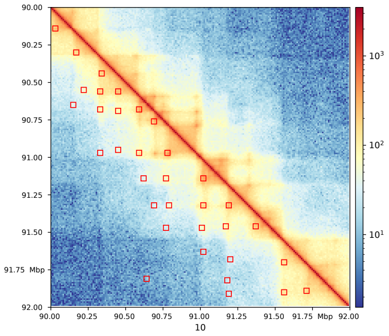

(c) chromosight

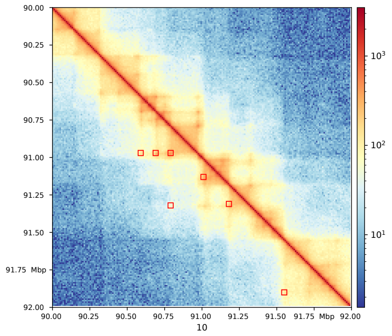

(d) cooltools

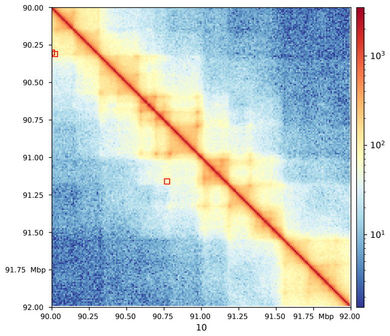

(e) Peakachu

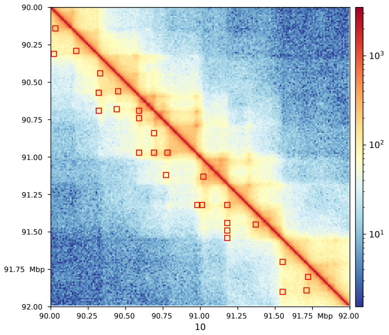

(f) Peakachu (p.b.a.)

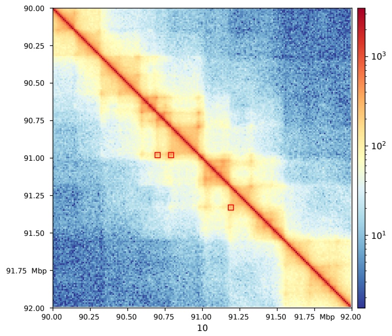

(g) Homer

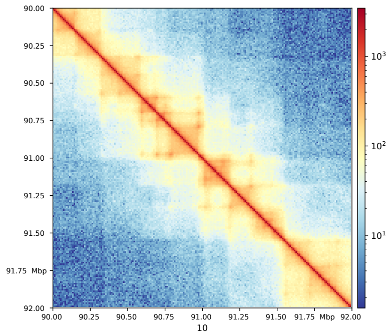

(h) FitHi-C 2

Figure S 8: The plot of chromosome 10 90.00 - 92.00 Mb on GM12878 and highlighted the detected loops from each software. HiCExplorer, HiCCUPS, and cooltools show similar results. Chromosight detects many loops in noisy regions and lacks specificity. The four loops of Peakachu show a general issue of this algorithm: The first two loops (18 Mb region) are in a region without enrichment, and the two others slightly miss the enriched interactions by a few kilobases. HOMER and Fit-Hi-C are not detecting any loop in the area. Plot with HiCExplorer hicPlotMatrix.

5 Runtimes

Dataset	HiCExplorer (2 MB)	HiCExplorer (8 MB)	HiCCUPS (GPU)	HiCCUPS (GPU rest.)	HiCCUPS (CPU)
GM12878 p+r					
	1:11 min	4:25 min	6:41 min	1:01 min	2:41 min
	2.7 GB	6.7 GB	34.8 GB	31.3 GB	27.7 GB
HMEC					
	0:52 min	1:22 min	7:20 min	0:40 min	4:13 min
	2.2 GB	2.8 GB	10.1 GB	13.9 GB	6.53 GB
HUVEC					
	0:58 min	1:47 min	7:07 min	0:39 min	3:28 min
	2.5 GB	3.3 GB	9.4 GB	14.1 GB	7.4 GB
IMR90					
	1:07 min	2:24 min	6:49 min	0:39 min	3:28 min
	2.7 GB	3.5 GB	10.3 GB	14.1 GB	10.2 GB
K562					
	1:03 min	2:15 min	6:32 min	0:37 min	2:33 min
	2.7 GB	3.5 GB	10.7 GB	15.4 GB	8.4 GB
KBM7					
	1:02 min	2:02 min	7:08 min	0:38 min	3:08 min
	2.3 GB	4 GB	14.4 GB	14.1 GB	6.7 GB
NHEK					
	0:56 min	1:44 min	7:09 min	0:39 min	3:36 min
	2.4 GB	4.1 GB	10.2 GB	10.9 GB	8.4 GB

(a) Runtime and memory for HiCExplorer and HiCCUPS.

Dataset	Homer	chromosight (2MB)	chromosight (8 MB)	cooltools (2MB)	cooltools (8MB)	Fit-Hi-C 2 (8 MB)	peakachu
GM12878 p+r							
	-	3:23 min	6:22 min	6:02 min (+ 6:11 min)	9:06 min (+ 6:11 min)	4:46 h + 20 min	7:03 h
	-	38.6 GB	39 GB	5.7 GB (20 GB)	6.5 GB (20 GB)	118 GB	59.5 GB
HMEC							
	11:58 min	1:03	2:01 min	2:14 min (+ 0:41 min)	3:52 min (+ 0:41 min)	37:03 + 5:32 min	1:34 h
	44.8 GB	6.1 GB	11.9 GB	2.6 GB (20 GB)	2.9 GB (20 GB)	13.8 GB	11.9 GB
HUVEC							
	15:44 min	1:06	2:12 min	2:04 (+1:00 min)	3:41 (+1:00 min)	51:06 + 7:24 min	2:13 h
	54 GB	9 GB	11.6 GB	2.6 GB (18.5 GB)	2.6 GB 2.9 GB (18.5 GB)	19.5	19 GB
IMR90							
	38:17 min	1:47 min	3:19 min	2:41 min (+1:34 min)	4:28 min (+1:34 min)	80 + 10:58 min	3:27 h
	102 GB	14 GB	14.8 GB	3.5 GB (20.1 GB)	3.8 GB (20.1 GB)	30.25 GB	31.6 GB
K562							
	30:40 min	1:27 min	2:43 min	2:16 (+1:22 min)	3:54 min(+1:22 min)	-	1:49h
	92.6 GB	12.1 GB	13.5 GB	2.3 GB(20 GB)	3.0 GB(20 GB)	-	33 GB
KBM7							
	35:12 min	1:17 min	2:36 min	2:24 min (+1:29 min)	4:12 min (+1:29 min)	1:19 h	2:49 h
	86.8	10.5 GB	12.4 GB	3.2 GB (20.1 GB)	3.2 GB (20.1 GB)	33 GB	24.5 GB
NHEK							
	21:56	1:18 min	2:26	2:14 (+1:15 min)	3:52 (+1:15 min)	59:39 min + 7:04 min	2:05 h
	71.5 GB	8.2 GB	12.2 GB	2.5 GB(19.9 GB)	2.7 GB (19.9 GB)	24 GB	18.7 GB

(b) Runtime and memory for Homer, chromosight and cooltools.

Table 8: Computed on different datasets from Rao et al. (2014) with KR on 10kb resolution, on AMD Ryzen 3700X 8 cores / 16 threads, 120 GB memory with Nvidia GTX 1070. HiCExplorer, Chromosight and cooltools was computed on 2 MB and 8 MB of genomic distance; HiCCUPS GPU on the full dataset, HiCCPUS CPU and 'GPU restrict' mode with a fixed size of 8 MB.

Dataset	HiCExplorer	HiCCUPS	Homer	chromosight	cooltools
GM12878 p+r					
	35:47 min	21:14 min	7:46:44 h min	22:59 min	32:17 (+9:59 min)
	1.5 GB	6.2 GB	98.7 GB	8.9 GB	1.45 GB (2.33 GB)
HMEC					
	13:22 min	40:43 min	35:24 min	8:27 min	21:16 min (+1:06 min)
	0.52 GB	2.2 GB	8 GB	2.5 GB	0.48 GB (1.75 GB)
HUVEC					
	16:58 min	33:33 min	44:52 min	9:30 min	21:31 (+1:34 min)
	0.62 GB	1.65 GB	9.9 GB	2.6 GB	0.46 GB (1.8 GB)
IMR90					
	22:52 min	27:33 min	1:37:54 h	12:24 min	22:35 (+2:26 min)
	0.86 GB	2.32 GB	102 GB	2.84 GB	0.6 GB (2.33) GB
K562					
	22:08 min	23:58 min	1:22:54 h min	10:59 min	10:49 (+2:08 min)
	0.88 GB	2.0 GB	23.4 GB	2.7 GB	0.48 GB (2.1 GB)
KBM7					
	20:18 min	30:00 min	1:23:04 h	10:44 min	22:51 min (+2:01 min)
	0.72 GB	2.2 GB	19.1 GB	2.7 GB	0.5 GB (2.6 GB)
NHEK					
	16:22 min	34:22 min	1:00:21 h	9:54 min	22:30 min (+1:56)
	0.6 GB	2.1 GB	13.4 GB	2.6 GB	0.49 GB (1.7 GB)

Table 9: Computed on different datasets from Rao et al. (2014) with KR on 10kb resolution with 8 MB maximum loop distance using only one thread. On AMD Ryzen 3700X 8 cores / 16 threads, 120 GB memory with Nvidia GTX 1070. HiCCUPS uses the CPU based version, Homer has no option to restrict the loop size and computes therefore on the full dataset.

6 Tool features

Tool	Multicore	variable distance	accuracy	CPU/GPU	memory usage	one click installation
HiCExplorer	yes	yes	high	CPU	low	yes (conda)
Juicer HiCCUPS	yes	no (all or 8 MB)	high	GPU	moderate	no (add. CUDA installation)
Juicer HiCCUPS CPU	yes	no (fixed to 8 MB)	high	CPU	moderate	yes
Homer	no	no (full genome)	low	CPU	very high	no
cooltools	yes	yes	high	CPU	low	yes
chromosight	yes	yes	low	CPU	moderate	yes
FitHiC2	no	yes	low	CPU	high	no (conda, add. tools via github)
peakachu	no	yes	high	CPU	moderate	yes (conda)

Table 10: Feature comparison of different loop detection tools

7 Data preparation and parameters

Hi-C matrices: Download from GSE63525 in *hic* format. Convertation to cool with *hic2cool* in version 0.8.2.

The processing workflow for example for IMR90:

Listing 1: hic2cool

```
hic2cool convert -r 10000 GSE63525_IMR90_combined.hic imr90.cool -p 16
```

Apply of KR correction factors to cooler's weight column with HiCExplorer hicConvertFormat:

Listing 2: hicConvertFormat

```
hicConvertFormat -m imr90.cool --inputFormat cool --outputFormat cool -o imr90_int_KR.cool
    ↪ --correction_name KR --enforce_integer
```

Loop detection with HiCExplorer's hicDetectLoop with default parameters except the loop size:

Listing 3: hicDetectLoops

```
hicDetectLoops -m imr90_int_KR.cool -o imr90_2mb_result.txt --maxLoopDistance 8000000 -t 16
    ↪  -tpc 8
```

Loop detection with HiCCUPS (GPU):

Listing 4: HiCCUPS GPU

```
java -jar juicer_tools_1.22.01.jar hiccups -r 10000 -k KR --threads 16
    ↪ GSE63525_IMR90_combined.hic imr90_results
```

Restricted mode:

Listing 5: HiCCUPS GPU restricted mode

```
java -jar juicer_tools_1.22.01.jar hiccups -r 10000 -k KR --threads 16 --restrict
    ↪ GSE63525_IMR90_combined.hic imr90_results_restrict
```

CPU mode:

Listing 6: HiCCUPS CPU

```
java -jar juicer_tools_1.22.01.jar hiccups -r 10000 -k KR --threads 16 --cpu
    ↪ GSE63525_IMR90_combined.hic imr90_results_cpu
```

Homer:

Listing 7: Homer's findTADsAndLoops

```
perl bin/findTADsAndLoops.pl find imr90_tag/ -cpu 16 -res 10000 -window 15000 -genome hg19
```

Chromosight:

Listing 8: Chromosight detect

```
chromosight detect --threads 16 --min-dist 40000 --pearson 0.3 --win-size 15 --min-
    ↪ separation 0 --max-dist 8000000 imr90_int_KR.cool chromsight_output_imr90
```

Cooltools

Listing 9: Cooltools compute-expected

```
cooltools compute-expected -p 16 imr90_int_KR.cool -o imr90_exp.tsv
```

Listing 10: Cooltools call-dots

```
cooltools call-dots --max-loci-separation 8000000 -p 16 --num-lambda-chunks 50 -o imr90.tsv
    ↪  imr90_int_KR.cool imr90_exp.tsv
```

Listing 11: Fit-Hi-C

```
fithic -i kbm7/fithic.interactionCounts.gz -f gm12878/fithic.fragmentMappability.gz -o
    ↪ gm12878/ -r 10000 --upperbound 800000
cd gm12878
zcat 1/subset_fithic_1.gz 2/subset_fithic_2.gz 3/subset_fithic_3.gz 4/subset_fithic_4.gz 5/
    ↪ subset_fithic_5.gz 6/subset_fithic_6.gz 7/subset_fithic_7.gz 8/subset_fithic_8.gz 9/
    ↪ subset_fithic_9.gz 10/subset_fithic_10.gz 11/subset_fithic_11.gz 12/subset_fithic_12
    ↪ .gz 13/subset_fithic_13.gz 14/subset_fithic_14.gz 15/subset_fithic_15.gz 16/
    ↪ subset_fithic_16.gz 17/subset_fithic_17.gz 18/subset_fithic_18.gz 19/
    ↪ subset_fithic_19.gz 20/subset_fithic_20.gz 21/subset_fithic_21.gz 22/
    ↪ subset_fithic_22.gz X/subset_fithic_X.gz > gm12878_loops.txt
```

Listing 12: Peakachu

```
peakachu score_genome -r 10000 --balance --upper 800 -p gm12878_KR.cool -O scores -m
    ↪ down100.ctcf.pkl
for i in scores/*; do peakachu pool -i $i -t .70 > ${i}.loops.txt; done
cat *.loops.txt > gm12878_loops.txt
```

CTCF data from various sources as narrow peak or broad peak files: Gm12878 from GSM935611; Hmec from GSM749753; Huvec from GSM749749; K562 from GSM733719 and Nhek from GSM733636. Sorting with

Listing 13: bedtools sort

```
bedtools sort -i hmec_total_pooled.broadPeak > hmec_sorted.bed
```

Correlation with CTCF with HiCExplorer validate locations:

Listing 14: HiCExplorer validate locations

```
hicValidateLocations -d hmec.tsv.postproc -p proteins/hmec_sorted.bed -cl -r 10000
```

To correlate to HiChIP and ChIA-PET data, the data needs to be transformed from a hic to a cool file, or from a text file to a cool file:

Listing 15: hic to cool

```
hic2cool convert -r 10000 proteins/hichip/GSE80820_HiChIP_GM_cohesin.hic proteins/hichip/
    ↪ GSE80820_HiChIP_GM_cohesin_10kb.cool
```

Listing 16: text to cool

```
hicConvertFormat --matrices proteins/ChiAPET/GSM1436265_RAD21_ENCFF002EMQ.txt --inputFormat
    ↪  2D-text --outputFormat cool --outFileName proteins/ChiAPET/
    ↪ GSM1436265_RAD21_ENCFF002EMQ_10kb.cool --resolutions 10000 --chromosomeSize hg19.
    ↪ chrom.sizes
```

Listing 17: Validation with a cool file

```
hicValidateLocations --data fithic/10/gm12878/gm12878_loops.txt --validationData proteins/
    ↪ hichip/GSE80820_HiChIP_GM_cohesin_10kb.cool -vt cool -r 10000
```

Validation of loop requires to remove the header of a loop detection file by some of the tools manually. To correct for the annotation name issues, a parameter to add or remove the 'chr' prefix for loops or the protein data is for some files required. Intersection computation via *bedtools intersect*.

HiCExplorer version 3.7 (beta), HiCCUPS version 1.22.01 (and CUDA 11.2), Homer version 4.11, chromosight version 1.4.1, cooltools version 0.3.2, bedtools 2.30.0, hic2cool 0.8.2, peakachu 1.1.4, Fit-Hi-C 2.0.7.

Peakachus trained models have been downloaded from their github repository: https://github.com/tariks/peakachu#using-peakachu-as-a-standard-loop-caller
