# Supplementary material for: Loop detection using Hi-C data with HiCExplorer
Source: Gigascience. 2022 Jul 9;11:giac061. doi: 10.1093/gigascience/giac061 (PMC9270730; doi:10.1093/gigascience/giac061)
Supplement: giac061_Response_to_Reviewer_Comments_Original_Submission [file giac061_response_to_reviewer_comments_original_submission.pdf]

Author's Response To Reviewer Comments

Close

Respond to the reviewers comments for 'Loop detection using Hi-C data with HiCExplorer'

General remark from the authors

We thank the reviewers for their comments and suggestions that helped us to improve the presented manuscript. We rewrote parts of the manuscript to make the scope of the publication and the used techniques more clear.

Detailed reply to the reviewers comments

Reviewer #1: Wolff et al. present the python version of HiCExplorer for loop detection. The algorithm is included in the Galaxy HiCExplorer webserver (Wolff et al. 2020), although the publication about the webserver did not describe the algorithm in detail. HiCExplorer uses the same donut approach as HiCCUPS (Rao et al. 2014) with a few notable differences. HiCExplorer selects candidate peaks based on the significance of the distance-corrected observed/expected ratio using a negative binomial model, and compares the peak's enrichment to its neighbourhood's using a Wilcoxon rank-sum test.

The method is appropriate for chromatin loop identification and it performs similarly to existing methods both in terms of computational requirements and specificity of the detected loops. However, the manuscript in its current format does not describe the method adequately, and the comparison with the other methods is limited and inconsistent. It would be good to describe each step of the method (filtering based on distance, candidate selection based on negative binomial test, additional filtering options, local enrichment testing using different neighbourhoods in a Wilcoxon rank-sum test).

Reviewer #1:
The graphical representation currently included for the algorithm is not informative for most of these steps.
Answer:
We changed the graphic to help the reader to better understand the graphic. However, the graphical description is only complete under consideration of the caption and friendly ask reviewer #1 to please consider this in their reasoning.

Reviewer #1:
For the scientific community, it would be more informative if this method's performance would be further analyzed. Even though it is mentioned that the loop detection greatly depends on the initial parameters, the results do not show how the parameters influence it.
Answer:
We showed the impact of the parameters in the section 'HiCExplorer candidate selection' and extended it.

Reviewer #1:
The comparison of HiCExplorer with other existing methods is inconsistent. Finally, the text would need heavy editing for language, clarity and minor spelling mistakes.

Answer:
Reviewer #1 lacks explaining to us why in their view the comparison to other methods is inconsistent. If such a claim is made, and no specific comment for this is listed in the 'specific comments' section, it is very hard for us to improve the paper at this point. We request clarification from reviewer #1 about this

comment.

Reviewer #1:
Specific comments:
The background does not clearly lay out the motivation behind designing this algorithm. There are similar existing methods that are fast. Why is it expected to detect chromatin loops better?

Answer:

HiCCUPS (and its reimplementation in cooltools) uses the Poisson distribution which is a discrete distribution. Hi-C data is discrete data, but the commonly preferred way to analyze Hi-C data is not to work on raw, but on corrected data. The correction of the data with e.g. ICE or KR transforms the discrete data to continuous, making the Poisson distribution not usable. HiCCUPS solves this by reverting the correction and by operating on the raw data. We think this is not appropriated and it is better to use a distribution that can handle the Hi-C data independent of the case it is discrete or continuous which we presented with the continuous negative binomial distribution. It behaves like the binomial distribution in case the Hi-C data is uncorrected and discrete.

The factorial factor of a Poisson distribution could have been also been replaced by a gamma function to make it continuous, however, count data tends to overdispersion. The Poisson distribution assumes the variance and expected value are equal, the Negative Binomial distribution allows different values. We added a figure in the supplements to show we have overdispersion in the raw data and therefore the continuous negative binomial distribution is the theoretically better choice.

Besides the mathematical arguments, the original goal to design this algorithm was that at the time the development started (late spring 2018), no tool supporting the detection of loop data with the from us preferred 'cooler' file format was existing, the well-known HiCCUPS algorithm was available only as a GPU version and searched the full genome; while in the study from the authors (Rao 2014) they wrote that 98% of the loops are in a 2 MB distance range. However, the algorithm was added to HiCExplorer in spring 2019, but HiCCUPS added an experimental CPU support (which is experimental until today) and other tools supporting the cooler format (cooltools, chromosight) were published too. Today, the loop detection of HiCExplore is 'yet another one'; however, we think for completeness and citation reasons, the details of this algorithm should be published in a peer-reviewed journal.
Moreover, the presented algorithm is faster than cooltools e.g. Gm12878 and 2 MB distance (1:11 min vs 12:13 min) and requires less memory (2.7 GB vs 20 GB); faster than chromosight (1:11 min vs 3:23) and requires significantly less memory (2.7 GB vs 38.6 GB). HiCCUPS using the CPU version and the 8 MB restriction is faster on GM12878 (4:25 vs 2:41) but needs more memory (6.7 GB vs 27.7 GB). On all other datasets, HiCExplorer restricted to 8 Mb is faster and requires less memory. Concerning the accuracy: Chromosight detects too many loops and is too unspecific, hiccups and cooltools have similar results to HiCExplorer.
HiCCUPS in the GPU version is the fastest one, however, it also needs significantly more memory. The loop detection of HiCExplorer can run on any notebook, while all other tools need high memory requirements which are not available in regular notebooks or desktop computers or need an Nvidia GPU supporting CUDA. Also, GPU access is not that common, and also sometimes difficult to get in cluster environments. Last, HiCCUPS is only available with the '.hic' file format, and export of cooler to hic is not possible. I asked the authors of Juicer if they provide anything, but I got no answer (https://groups.google.com/g/3d-genomics/c/jCSQk4oEl5w/m/gqyJD0FOBwAJ). To rebuild the Hi-C matrices from scratch just for Juicer's HiCCUPS algorithm (and similar for Homer text file based solution) is not only time consuming but can add potential errors if different Hi-C matrix build tools classify some reads in another way (e.g. if it is a dangling end or similar Hi-C errors).

Reviewer #1:
This is not a 3D genomics specialized journal, therefore the text should introduce Hi-C and its challenges clearly. For example, the notion that genome properties and ligations affect Hi-C data analysis is mentioned in the methods section without further elaboration. It would be hard for readers to understand why authors are normalizing for ligation events in their algorithm.
Answer:
We specified this better. We want to accentuate that the different expected value computation methods are offered, and it is the choice of the user the select one which they think fits best to the data. Our investigation shows the mean is the best way to compute it, however, we do not arrogate what is the

best decision for an individual dataset and offer therefore all three methods.

Reviewer #1:
The background introduces a few methods that are not aimed at detecting chromatin loops (e.g. GOTHiC) or not designed for Hi-C (e.g. cLoops) and are also not used in the comparison. It would be more useful to describe the algorithms of those methods that are comparable to Hi-C explorer in terms of their goal and design.
Answer:
Reviewer #1 states one comment above this one, that this journal is not a 3D genomic specialized one and therefore more explanation should be added. We think the way we describe additional algorithms which are for the inexperienced reader very similar to what we present, is necessary for the reader to understand why these algorithms are not considered. Moreover, we describe algorithms the algorithms which are part of the comparison.

Reviewer #1:
Figure 1, which represents the steps of the algorithm, does not make it clear what happens at each step, some of arrows seem to point to random pixels, e.g. in panel C.
Answer:
The arrows in figure 1C do not point to random pixels, they point to the genomic distances, and these are given in the matrix by the diagonals. The caption describes it:'Fit cNB distribution per relative distance.' If reviewer #1 thinks the graphic irritates more than it helps, we can remove it and rely only on the textual description of the algorithm instead of a graphical one. However, the graphical description of the algorithm is only complete under the consideration of the caption, which reviewer #1 obviously did not consider.

Reviewer #1:
More elaboration on the use of the three different expected value calculation methods would be needed. Which one is more appropriate for a mammalian vs. an insect Hi-C does it depend on the genome size, the sequencing depth or the sparsity of the data?
Answer:
We extended the section 'HiCExplorer candidate selection' to address this comment.

Reviewer #1:
The negative binomial distribution does model well the read counts in most high-throughput sequencing experiments, but the rationale given for choosing it is not appropriate.
Answer:
We added an additional explanation and an overdispersion test with the Poisson distribution to show why we use it. However, reviewer #1 writes the whole time in their critics of a 'negative binomial' distribution. This is wrong and is one major aspect of our proposed algorithm. We use a continuous negative binomial distribution and not a discrete negative binomial distribution. This was explained in the manuscript and implicates, in combination with Hi-C correction like ICE or KR, already the rationale of choosing it. We encourage reviewer #1 to consider this major difference especially in comparison to HiCCUPS and their ignoring of the correction factors.

Reviewer #1:
Also, citing a stackexchange discussion for the methods is not suitable.
The numbers in most tables could be better appreciated if they were represented in a figure.
Answer:

We are aware that a citation of stackexchange is not optimal; we contacted the author of the post, Prof. Gordon Smyth from WEHI and he wrote that we should cite:

McCarthy, DJ, Chen, Y, Smyth, GK (2012). Differential expression analysis of multifactor RNA-Seq experiments with respect to biological variation. Nucleic Acids Research 40, 4288-4297.

We had the citation already in the manuscript and removed the reference to stackexchange. We do not want to claim the replacement of the binomial coefficient with the gamma function is our work, therefore we leave it to the editor to decide if we shall cite it or if the solution as proposed by Prof. Smyth is the appropriate way.

Reviewer #1:
What was the reason to increase the distance only to 8Mb instead of using the full genome as comparison, especially given that some of the compared methods only work on the full genome?
Answer:

The authors of HiCCUPS write in their own study (Rao 2014) that 98% of all loops are detected within the range of 2 MB. However, their GPU tool ignored this for quite some time and computed it on the full genome. With a newer version, an experimental labeled CPU version with the 8 MB restriction was published and the GPU version got the restriction as an option too. HiCCUPS does not allow to use any other distance than these 8 Mb, and for this reason, we compared the results to 8 Mb. Our tool is able to compute the loops within every distance the user is defining it.
Moreover, we stated already in the text why we do not investigate the full chromosomes in detail:

'If the restriction of the genomic distance between two loci is removed for HiCExplorer and all intra-chromosomal contacts are considered, the number of candidates to be tested increases by a factor of 10, but the number of accepted peaks increased only minor.'

Reviewer #1:
The bottom left neighbourhood in HiCCUPS is assessed, because they only use the upper triangle in the Hi-C matrix, and the bottom left neighbourhood represents the shorter interactions. In Figure 2, the detected interactions are indicated on the bottom triangle , which is counterintuitive.
Answer:
We do not think that computational details (upper triangle in the computation) and the visualization of data in the lower triangle confuse the readers. However, visualization in the bottom triangle is quite common, for example:
HiCCUPS, Rao 2014, Figure 3 https://www.cell.com/fulltext/S0092-8674(14)01497-4
Chromosight , Matthey-Doret 2020, Figure 2 https://www.nature.com/articles/s41467-020-19562-7

Reviewer #1:
Fig 2A is showing the same data as Fig 2A in the Galaxy HiCExplorer publication (Wolff et al 2020), but the detected loops indicated are different. What is the reason for that?
Answer:
The algorithm used in the Galaxy HiCExplorer 3 publication was based on HiCExplorer 3.2; with HiCExplorer 3.5 we changed the loop detection algorithm to its current form. For this reason, the detect loops differ. We changed the algorithm because we were not happy with the performance in terms of accuracy of the detect loops and also on the utilization of the threading of modern CPUs. For comparison of the algorithmic differences, please compare the manuscript to the bioRxiv publication of the loop detection.

Reviewer #1:
The difference between the proportion of CTCF-bound loops for the different methods is probably not significant. It should be tested.

Answer:

We have stated the proportion of the CTCF for each of the different methods. We think this was not recognized by reviewer #1 or we hereby ask for clarification of their comment.

Reviewer #2: This paper provided a loop detection method using continuous negative binomial function combined with donut approach. To test the performance of this method, the authors used in-situ Hi-C data by Rao 2014 in GM12878, K562, IMR90, HUVEC, KBM7, NHEK and HMEC cell lines. This method showed comparable results with HiCCUPS and cooltools and better outputs than HOMER and chromosight. The significant advantage is the utilization of modern computational resources. The following are my comments:

Reviewer #2:

1. The author claimed the advantages in utilizing computational resources. The authors need to clarify how their algorithm contributes to this advantage.

Answer:
We did this in the ‘comparison to competitors section’:

“For this reason, HiCExplorers’ hicDetectLoop does support the parallelization by not only the chromosomes but also an intra-chromosomal parallelization.”

We extended the explanation a bit. Also, the lower memory usage is clearly described in the text and in the tables.

Reviewer #2:
2. It will be helpful for the users to know the performance of the software at various sequencing depths, which can be achieved by down-sampling the high resolution datasets.
Answer:

We compare in the submitted manuscript different cell types which have all different sequencing depths for exactly this reason and this was already discussed in the section ‘HiCExplorer candidate selection’, starting at “For other cell lines published by Rao 2014‟; please also consider Supplementary Table 7 where the different read coverages are listed.

Reviewer #2:
3. The authors need to compare (or at least discuss) Fit-Hi-C and Peakchachu. A table showing the strength and limitation of each method will be helpful.
Answer:
We think reviewer #2 means the tool ‘Peakachu’ (https://github.com/tariks/peakachu) and not ‘Peakchachu’. The last one did not show any results on Google.

Fit-Hi-C is a method to detect significant Hi-C contacts. In our understanding this is very similar to GOTHiC. However, we gave it a try and on a Gm12878 10kb dataset, it detected significant contacts in the 100,000-ends. With an additional filter step loops can be detected. However, these detected loops have a low correlation to CTCF, overall the tool is not good in detecting enriched regions.

Peakachu: There is a large difference in the results that the authors present in their publication (https://www.nature.com/articles/s41467-020-17239-9) and the results we have, especially the comparison to HiCCUPS. One explanation is the provided trained model of Peakachu. We think the results of the detection rate and the correlation of the loop with orthogonal data show the provided model does not generalize well. Even more, a reason could be the approach the authors of Peakachu used in their publication: Instead of letting Fit-Hi-C and HiCCUPS compute their loops and compare detected loop locations with each other, they took all ‘candidate’ locations of the two algorithms and used their own ‘merging’ algorithm to filter out the loop locations. We think this is methodically critical, and are irritated why this has been accepted by the reviewers. Instead of a full comparison of the algorithms, only the first detection was from Fit-Hi-C and HiCCUPS, but the second one was from the authors of Peakachu. (Section ‘Methods’ of the Peakachu publication, ‘Loop detection with HiCCUPS and Fit-HiC’: ‘To make a fair comparison with Peakachu and HiCCUPS on the 100% matrix, we sorted the detected interactions by p-values and performed the same pooling algorithm used by Peakachu’)

Based on the performance of Fit-HiC and Peakachu on the Gm12878 dataset we decided to not test them on the other cell types in detail.

Reviewer #2:
To be honest, I don't think any method is clearly better than the other. They are just different approaches.
Answer: We agree with this viewpoint on the algorithms and therefore we present our manuscript in GigaScience as a Technical Note. To quote the criteria of the journal:

“The tool or method needs to have been tested, and properly compared to any existing tools or methods used by the community. It does not necessarily have to outperform existing approaches, but it should show innovation in the approach, implementation, or have added benefits that have been needed in this arena.”
https://academic.oup.com/gigascience/pages/technical_note

We think to fulfill these requirements. HiCCUPS and cooltools ignore the correction factors and uses a discrete distribution assumption and ignore the overdispersion in the data. By choosing a more appropriate model that is also able to work with continuous data, the continuous negative binomial distribution provides a mathematical better model for Hi-C loop detection. Moreover, we provide a CPU-based software enabling many researchers without access to Nvidia GPUs to perform our software. Also, HiCExplorer does not require training with additional and orthogonal data like ‘Peakachu’ which might be not available. We could also show how strong the dependency on a good trained model for Peakachu is. HiCExplorer outperforms Homer, Fit Hi-C and Peakachu by a large amount, time, memory, and accuracy wise.

Reviewer #2:
4. It is better to use other types of orthogonal data like HiChIP, ChIA-PET to evaluate the loops called by these methods. There are H3K27ac HiChIP, SMC1 HiChIP, CTCF ChIA-PET and RAD21 ChIA-PET data in GM12878.
Answer:
We added another table to the Supplementary Material comparing to the listed data for Gm12878 only. The accuracy scores confirm our claims of the CTCF ChIP-Seq data of the submitted manuscript.

Reviewer #2:
5. Just a minor suggestion. There are a lot of tables in the manuscript, which makes it hard for the readers to compare. It might be better to use figures instead.

Answer:
We added a few graphics to replace some tables. Other tables have been moved to the Supplementary Material.

Close
