# Supplementary material for: Loop detection using Hi-C data with HiCExplorer
Source: Gigascience. 2022 Jul 9;11:giac061. doi: 10.1093/gigascience/giac061 (PMC9270730; doi:10.1093/gigascience/giac061)
Supplement: giac061_Response_to_Reviewer_Comments_Revision_1 [file giac061_response_to_reviewer_comments_revision_1.pdf]

Author's Response To Reviewer Comments

Close

Suggestions by Editor after consulting external reviewer
- The authors should add to their last figure a comparison of the HiCExplorer and HICUPS to the Peakachu run on the ICE corrected matrices stating it explicitly in the figure legend.

Anwer
We did this.

- Please include more comparisons showing what HiCExplorer calls and what is missed by other tools. Add at least one more locus-by-locus snapshot of loops called by the tools, including Fit-Hi-C method absent in Figure 4. It would be important to better illustrate the Venn Diagram results from figure 2.

Anwer
We did this. Please consider the additional new figures in the Supplementary material.

- Another potentially important figure would be to include calls before merging, displaying several examples of enriched pixels in all the three methods (Fit-Hi-C, HiCExplorer and HiCCUPS). This could help resolving the issue raised by Wolff et al and pertaining to the method used to merge significant pixels into a single loop. Perhaps this should make up an entire small chapter of the paper (depending on the data).
Anwer
We don’t think intermediate data is important in this comparison. The issue that we raised with the merging of loops is a different one: We think that all tools should be compared based on their detected loops and this implies that the merging of loops is applied by individual tools. What we raised and what we are concerned about is a sentence in the publication of Peakachu stating they used the intermediate, unmerged loop candidates and applied for all their self-developed merging tool. This is biasing the results in a positive way towards Peakachu. However, this is an issue of the Peakachu publication and does not influence our comparison.

Reviewer #1

Reviewer #1: My main concern for the revised manuscript is the additional benchmarking the authors performed with Fit-Hi-C and Peackachu. Since Fit-Hi-C is one of the first algorithms for Hi-C loop prediction (published in 2014) and Peakachu is the only method that uses the supervised machine learning approach for such purpose, I suggested that these two software should be recognized. If the authors can perform a fair benchmarking and find out where the differences come from, the results would be really interesting.

The authors decided to test the aforementioned methods during the revision. Unfortunately, I believe there were some errors during the testing.

For Peakachu:

1. Most importantly, the authors used the wrong form of normalized Hi-C files for Peakachu. Peakachu model was trained and should be used with ICE-normalized Hi-C matrix. However, based on page 8 in the supplementary file, the input file is gm12878_KR.cool. The data range for ICE and KR normalization is very different, and therefore, the model trained in ICE file will not work with KR format and the prediction will wrong. Therefore, all the following evaluations and descriptions for the Peakachu prediction are not accurate and needs to be revised (such as Fig. 4, Table S1 ...).

Answer
If your tool is that sensible then one of the following should be considered:
Publish the information that you need an ICE corrected matrix ANYWHERE! It is not published on your GitHub repository where you provide the models, it is not written in the paper, nor in the supplementary material! How should someone know this?
ICE and KR are not that different, I suspect a bit that you trained on not upscaled matrices. Can it be that you trained on a matrix with a value range of 0 - 1, as it is after the correction of ICE and KR if the correction tool does not scale the values back to the correct value range? If so, please make this information public, because it is quite common to work on scaled-back interaction data.
Even this is all not true: An observed/expected normalization should be able to catch most of the differences of ICE / KR correction. In the end, we just search for enriched interactions, the correction does not matter that much. See the comment of the external reviewer concerning this!
If the above still is wrong: Then I am sorry to say this, but I assume your model has a heavy overfitting issue.
I will not revise the results based on the KR corrected matrix. It seems to me that either you missed important normalization (e.g. obs/exp normalization.) in the pre-processing or your model has heavy overfitting. Concerning obs/exp normalization: we checked your publication and supplementary material, we did not find the term 'observed/expected matrix' or something similar anywhere. We, therefore, assume you don't apply this normalization.

Reviewer #1
2. In the response letter, there is another misunderstanding about merging. Because Fit-Hi-C predicted too many contacts, the authors of Peakachu merged "the top 140,000 interactions into 14,876 loops (Fig. 3a, b), with the same pooling algorithm used by Peakachu." The reason is that if multiple continuous bins on a Hi-C map are all predicted as loops, the merging/filtering step will use the bin with the most significant P-value as the chromatin loops (local minimal). As the authors noted, Fit-Hi-C by default will generate "significant contacts in the 100,000-ends." Therefore, this merging/filtering step is necessary if we want to compare the loops predicted by each method. This is also what the author did in this manuscript as well - I am quoting their own writing here, "This filtering step is necessary to address the candidate peak value as a singular outlier within the neighborhood." Therefore, I do not understand the authors are "irritated" by such approach.

Answer
Yes, we merged the significant detected candidates too. However, we used for all approaches the merging tool provided by the individual tool and not for all tools our own merging tool. This is a major difference! To use one merging tool, their own developed one (!!!), is the approach of Peakachu paper and this is what we criticize! We do not criticize the merging in general. Fit-HiC2 provides its own merging tool, so does HiCCUPS. Why have you not used it? That would have been a fair comparison!

Reviewer #1
3. The authors of Peakach have released their prediction in 56 Hi-C datasets on their 3D Genome Browser website (http://3dgenome.fsm.northwestern.edu/publications.html), including the ones used in this manuscript. The authors used models trained at different sequencing depths for different datasets. Therefore, I would suggest the authors use this dataset for a fair evaluation.
Answer
We included this data. The very high agreement of loops with positions of HiChIP based H3K27ac and SMC1 confirms our suspicion of heavy overfitting. Your model does not learn the pattern of loops but the pattern of locations where these two proteins are present. In contrast to this, your performance considering CTCF with 50% is low, compared to HiCExplorer or HiCCUPS with 64% and 61%. Also the loci specific investigation shows that you miss certain clear loops like on chromosome 4 20.55 - 22.55 MB (Supplementary Figure 4) but you detect many loops which are not clearly visible and would need further orthogonal data to be confirmed, for example, the loops in the loci chromosome 1 15.00 - 18.00 MB (Supplementary Figure 6). We suggest you update your published models based on training where the Hi-C interaction matrices are obs / exp normalized and have different corrections as their base. Please take care that important and easily visible loops on chromosome 4 20.55 - 22.55 MB are identified.

Reviewer #1
Regarding Fit-Hi-C, what are the number of peaks the before and after filtering? The author also needs to provide the loop locations so that reviewers can evaluate their claim independently. This information is critical. This manuscript might be helpful for the authors to evaluate Fit-Hi-C (Arya Kaul et al. Nature

Protocol 2020).

Finally, the authors need to provide all the predicted chromatin loops in the cell lines as well as loops predicted by other software used in this manuscript as supplementary materials (loops in Supplementary Table 1).
Answer
We published the data on zenodo.

Reviewer 2

The authors have addressed some of my comments, but the majority of my comments have not been satisfactorily addressed. The manuscript would still require major modification before publication.

Reviewer #2:
I am, of course, fully aware that a figure and its caption belong together, and my concern referred to the figure and its caption text as a whole. However, the graphics, even in their updated version, do not facilitate the readers' understanding of the method. Furthermore, it is customary in scientific writing that the use of arrows or other markers in the graphics is accompanied by an explanation in the caption text (i.e. what does each arrow point to?). Also, instead of the use of arrows, it would be much more informative to demarcate the borders of those areas in the matrices that the authors would like to highlight.
Perhaps, a pragmatic solution could be to delete the confusing graphics altogether and just use the caption text on its own as bullet points or numbered steps for the method.
Answer
We don't think we come here to any agreement with the reviewer. We dropped the graphic without any replacement. The algorithm itself is already described in the text and the graphical description was to have additional material to understand it maybe better. We reject the suggestion to use bullet points because it would be even more redundant.

Reviewer #2:

As pointed out, some of the methods search for loops genome-wide or only within 8Mb windows, while others use a custom distance for the loop search space. Currently the results show loops detected genome-wide for Juicer GPU, within 8Mb for Juicer CPU, genome-wide for HOMER and within 8Mb and 2Mb for the other methods. Comparison of the methods should be restricted to those loops that are shorter than 8Mb/2Mb for all methods and the genome-wide results could be added in supplementary.
Answer:
We restrict all to 8 MB.

Reviewer #2:

Thank you for extending the section. However, the explanation about the chimeric reads is incorrect. Read-pairs are indeed always come from ligation fragments, but chimeric reads is a term used for those reads (single end) which overlap with the ligation site, and therefore cannot be mapped to the genome without trimming or splitting.

Answer:
We added a sentence.

Reviewer #2:

I am not convinced that a passing introduction of a few methods that are not designed to detect loops in Hi-C data will help the inexperienced reader to understand why they are not appropriate. By the authors reasoning, several other methods should also be introduced e.g. FIREcaller, SIP, Mustache, HiC-DC, HIPPIE.
Answer:
We removed the methods that are criticized in the introduction.

Reviewer #2:

As the authors themselves stated above in their reply to my question about the motivation of developing the algorithm. They choose the continuous negative binomial distribution because the negative binomial allows for overdispersion of the data and the continuous version of it can be applied to normalized read counts.
What this comment referred to, was the following sentence: “In genome analysis, good experience has been made with negative binomial functions as proposed, for example, by DESeq2.” This does not describe the overdispersion issue which is the main reason for using a negative binomial based algorithm. In this case a continuous negative binomial.

Answer:

We never claimed that DESeq2 describes the overdispersion issue. The sentence was written why we consider a cNB distribution at all and to motivate that it has been successfully used in other areas of genome analysis. Anyhow, we removed the sentence to satisfy the reviewer.

Reviewer #2:

In this case, I would suggest comparing the tools for loops detected within 2Mb and 8Mb and show the full genome results only in the supplementary. See my comment above about the consistency of the comparison.

Answer:
We restrict the analysis results to 8Mb and dropped all other results.

Reviewer #1:
Fig 2A is showing the same data as Fig 2A in the Galaxy HiCExplorer publication (Wolff et al 2020), but the
detected loops indicated are different. What is the reason for that?
Answer:
The algorithm used in the Galaxy HiCExplorer 3 publication was based on HiCExplorer 3.2; with HiCExplorer 3.5
we changed the loop detection algorithm to its current form. For this reason, the detect loops differ. We changed
the algorithm because we were not happy with the performance in terms of accuracy of the detect loops and also
on the utilization of the threading of modern CPUs. For comparison of the algorithmic differences, please compare the manuscript to the bioRxiv publication of the loop detection.
The authors should highlight these changes in current manuscript.
Answer
We added two sentences.

Reviewer #2:
The authors state that some methods are better correlated with CTCF binding sites than others based on the proportion of CTCF-bound loops. I did notice that the proportion was calculated for each method. However, the difference between these proportions would have needed to be statistically tested (with two-proportions Z-test) to claim differences in the methods’ performance.

Answer
We added the requested z-test for the GM12878 data and the proportions of CTCF ChIA-PET, RAD21 ChIA-PET, H3K27ac HiChIP and SMC1 HiChIP. Given a p-value threshold of 0.05 all results are below this threshold for a H0 ‘the proportions are the same’. Given a p-value threshold of 0.001 only the differences in proportion between HiCExplorer and HiCCUPS for the CTCF ChIA-PET with 0.00216 and HiCExplorer and Homer for the SMC1 HiChIP data with 0.00299 are not significant.

Close
