# Supplementary material for: Loop detection using Hi-C data with HiCExplorer
Source: Gigascience. 2022 Jul 9;11:giac061. doi: 10.1093/gigascience/giac061 (PMC9270730; doi:10.1093/gigascience/giac061)
Supplement: giac061_Response_to_Reviewer_Comments_Revision_2 [file giac061_response_to_reviewer_comments_revision_2.pdf]

Author's Response To Reviewer Comments

Close

Dear editorial board,

I hereby submit the minor revision with the requested changes.

Your sincerely,

Joachim Wolff

Close
