# Supplementary material for: Loop detection using Hi-C data with HiCExplorer
Source: Gigascience. 2022 Jul 9;11:giac061. doi: 10.1093/gigascience/giac061 (PMC9270730; doi:10.1093/gigascience/giac061)
Supplement: giac061_Reviewer_1_Report_Original_Submission — Borbala Mifsud -- 3/22/2021 Reviewed [file giac061_reviewer_1_report_original_submission.pdf]

Reviewer Report

Title: Loop detection using Hi-C data with HiCExplorer

Version: Original Submission **Date:** 3/22/2021

Reviewer name: Borbala Mifsud

Reviewer Comments to Author:

Wolff et al. present the python version of HiCExplorer for loop detection. The algorithm is included in the Galaxy HiCExplorer webserver (Wolff et al. 2020), although the publication about the webserver did not describe the algorithm in detail. HiCExplorer uses the same donut approach as HiCCUPS (Rao et al. 2014) with a few notable differences. HiCExplorer selects candidate peaks based on the significance of the distance-corrected observed/expected ratio using a negative binomial model, and compares the peak's enrichment to its neighbourhood's using a Wilcoxon rank-sum test. The method is appropriate for chromatin loop identification and it performs similarly to existing methods both in terms of computational requirements and specificity of the detected loops. However, the manuscript in its current format does not describe the method adequately, and the comparison with the other methods is limited and inconsistent. It would be good to describe each step of the method (filtering based on distance, candidate selection based on negative binomial test, additional filtering options, local enrichment testing using different neighbourhoods in a Wilcoxon rank-sum test). The graphical representation currently included for the algorithm is not informative for most of these steps. For the scientific community, it would be more informative if this method's performance would be further analyzed. Even though it is mentioned that the loop detection greatly depends on the initial parameters, the results do not show how the parameters influence it. The comparison of HiCExplorer with other existing methods is inconsistent. Finally, the text would need heavy editing for language, clarity and minor spelling mistakes.

Specific comments:

The background does not clearly lay out the motivation behind designing this algorithm. There are similar existing methods that are fast. Why is it expected to detect chromatin loops better?

This is not a 3D genomics specialized journal, therefore the text should introduce Hi-C and its challenges clearly. For example, the notion that genome properties and ligations affect Hi-C data analysis is mentioned in the methods section without further elaboration. It would be hard for readers to understand why authors are normalizing for ligation events in their algorithm.

The background introduces a few methods that are not aimed at detecting chromatin loops (e.g. GOTHiC) or not designed for Hi-C (e.g. cLoops) and are also not used in the comparison. It would be more useful to describe the algorithms of those methods that are comparable to Hi-C explorer in terms of their goal and design.

Figure 1, which represents the steps of the algorithm, does not make it clear what happens at each step, some of arrows seem to point to random pixels, e.g. in panel C.

More elaboration on the use of the three different expected value calculation methods would be needed. Which one is more appropriate for a mammalian vs. an insect Hi-C does it depend on the

genome size, the sequencing depth or the sparsity of the data?
The negative binomial distribution does model well the read counts in most high-throughput sequencing experiments, but the rationale given for choosing it is not appropriate. Also, citing a stackexchange discussion for the methods is not suitable.
The numbers in most tables could be better appreciated if they were represented in a figure.
What was the reason to increase the distance only to 8Mb instead of using the full genome as comparison, especially given that some of the compared methods only work on the full genome?
The bottom left neighbourhood in HiCCUPS is assessed, because they only use the upper triangle in the Hi-C matrix, and the bottom left neighbourhood represents the shorter interactions. In Figure 2, the detected interactions are indicated on the bottom triangle , which is counterintuitive.
Fig 2A is showing the same data as Fig 2A in the Galaxy HiCExplorer publication (Wolff et al 2020), but the detected loops indicated are different. What is the reason for that?
The difference between the proportion of CTCF-bound loops for the different methods is probably not significant. It should be tested.

Level of Interest

Please indicate how interesting you found the manuscript: Choose an item.

Quality of Written English

Please indicate the quality of language in the manuscript: Choose an item.

Declaration of Competing Interests

Please complete a declaration of competing interests, considering the following questions:

- Have you in the past five years received reimbursements, fees, funding, or salary from an organisation that may in any way gain or lose financially from the publication of this manuscript, either now or in the future?
- Do you hold any stocks or shares in an organisation that may in any way gain or lose financially from the publication of this manuscript, either now or in the future?
- Do you hold or are you currently applying for any patents relating to the content of the manuscript?
- Have you received reimbursements, fees, funding, or salary from an organization that holds or has applied for patents relating to the content of the manuscript?
- Do you have any other financial competing interests?
- Do you have any non-financial competing interests in relation to this paper?

If you can answer no to all of the above, write 'I declare that I have no competing interests' below. If your reply is yes to any, please give details below.

I declare that I have no competing interests.

I agree to the open peer review policy of the journal. I understand that my name will be included on my report to the authors and, if the manuscript is accepted for publication, my named report including any attachments I upload will be posted on the website along with the authors' responses. I agree for my report to be made available under an Open Access Creative Commons CC-BY license (http://creativecommons.org/licenses/by/4.0/). I understand that any comments which I do not wish to be included in my named report can be included as confidential comments to the editors, which will not be published.

Choose an item.

To further support our reviewers, we have joined with Publons, where you can gain additional credit to further highlight your hard work (see: https://publons.com/journal/530/gigascience). On publication of this paper, your review will be automatically added to Publons, you can then choose whether or not to claim your Publons credit. I understand this statement.

Yes Choose an item.
