# Supplementary material for: Loop detection using Hi-C data with HiCExplorer
Source: Gigascience. 2022 Jul 9;11:giac061. doi: 10.1093/gigascience/giac061 (PMC9270730; doi:10.1093/gigascience/giac061)
Supplement: giac061_Reviewer_2_Report_Original_Submission — Feng Yue -- 4/4/2021 Reviewed [file giac061_reviewer_2_report_original_submission.pdf]

Reviewer Report

Title: Loop detection using Hi-C data with HiCExplorer

Version: Original Submission **Date:** 4/4/2021

Reviewer name: Feng Yue

Reviewer Comments to Author:

This paper provided a loop detection method using continuous negative binomial function combined with donut approach. To test the performance of this method, the authors used in-situ Hi-C data by Rao 2014 in GM12878, K562, IMR90, HUVEC, KBM7, NHEK and HMEC cell lines. This method showed comparable results with HiCCUPS and cooltools and better outputs than HOMER and chromosight. The significant advantage is the utilization of modern computational resources. The following are my comments:

1. The author claimed the advantages in utilizing computational resources. The authors need to clarify how their algorithm contributes to this advantage.
2. It will be helpful for the users to know the performance of the software at various sequencing depths, which can be achieved by down-sampling the high resolution datasets.
3. The authors need to compare (or at least discuss) Fit-Hi-C and Peakchachu. A table showing the strength and limitation of each method will be helpful. To be honest, I don't think any method is clearly better than the other. They are just different approaches.
4. It is better to use other types of orthogonal data like HiChIP, ChIA-PET to evaluate the loops called by these methods. There are H3K27ac HiChIP, SMC1 HiChIP, CTCF ChIA-PET and RAD21 ChIA-PET data in GM12878.
5. Just a minor suggestion. There are a lot of tables in the manuscript, which makes it hard for the readers to compare. It might be better to use figures instead.

Level of Interest

Please indicate how interesting you found the manuscript: Choose an item.

Quality of Written English

Please indicate the quality of language in the manuscript: Choose an item.

Declaration of Competing Interests

Please complete a declaration of competing interests, considering the following questions:

- Have you in the past five years received reimbursements, fees, funding, or salary from an organisation that may in any way gain or lose financially from the publication of this manuscript, either now or in the future?

- Do you hold any stocks or shares in an organisation that may in any way gain or lose financially from the publication of this manuscript, either now or in the future?
- Do you hold or are you currently applying for any patents relating to the content of the manuscript?
- Have you received reimbursements, fees, funding, or salary from an organization that holds or has applied for patents relating to the content of the manuscript?
- Do you have any other financial competing interests?
- Do you have any non-financial competing interests in relation to this paper?

If you can answer no to all of the above, write 'I declare that I have no competing interests' below. If your reply is yes to any, please give details below.

I declare that I have no competing interests.

I agree to the open peer review policy of the journal. I understand that my name will be included on my report to the authors and, if the manuscript is accepted for publication, my named report including any attachments I upload will be posted on the website along with the authors' responses. I agree for my report to be made available under an Open Access Creative Commons CC-BY license (http://creativecommons.org/licenses/by/4.0/). I understand that any comments which I do not wish to be included in my named report can be included as confidential comments to the editors, which will not be published.

Choose an item.

To further support our reviewers, we have joined with Publons, where you can gain additional credit to further highlight your hard work (see: https://publons.com/journal/530/gigascience). On publication of this paper, your review will be automatically added to Publons, you can then choose whether or not to claim your Publons credit. I understand this statement.

Yes Choose an item.
