# Supplementary material for: Loop detection using Hi-C data with HiCExplorer
Source: Gigascience. 2022 Jul 9;11:giac061. doi: 10.1093/gigascience/giac061 (PMC9270730; doi:10.1093/gigascience/giac061)
Supplement: giac061_Reviewer_2_Report_Revision_1 — Feng Yue -- 7/19/2021 Reviewed [file giac061_reviewer_2_report_revision_1.pdf]

Reviewer Report

Title: Loop detection using Hi-C data with HiCExplorer

Version: Revision 1 **Date:** 7/19/2021

Reviewer name: Feng Yue

Reviewer Comments to Author:

My main concern for the revised manuscript is the additional benchmarking the authors performed with Fit-Hi-C and Peackachu. Since Fit-Hi-C is one of the first algorithms for Hi-C loop prediction (published in 2014) and Peakachu is the only method that uses the supervised machine learning approach for such purpose, I suggested that these two software should be recognized. If the authors can perform a fair benchmarking and find out where the differences come from, the results would be really interesting. The authors decided to test the aforementioned methods during the revision. Unfortunately, I believe there were some errors during the testing.

For Peakachu:

1. Most importantly, the authors used the wrong form of normalized Hi-C files for Peakachu. Peakachu model was trained and should be used with ICE-normalized Hi-C matrix. However, based on page 8 in the supplementary file, the input file is gm12878_KR.cool. The data range for ICE and KR normalization is very different, and therefore, the model trained in ICE file will not work with KR format and the prediction will wrong. Therefore, all the following evaluations and descriptions for the Peakachu prediction are not accurate and needs to be revised (such as Fig. 4, Table S1 ...).
2. In the response letter, there is another misunderstanding about merging. Because Fit-Hi-C predicted too many contacts, the authors of Peakachu merged "the top 140,000 interactions into 14,876 loops (Fig. 3a, b), with the same pooling algorithm used by Peakachu." The reason is that if multiple continuous bins on a Hi-C map are all predicted as loops, the merging/filtering step will use the bin with the most significant P-value as the chromatin loops (local minimal). As the authors noted, Fit-Hi-C by default will generate "significant contacts in the 100,000-ends." Therefore, this merging/filtering step is necessary if we want to compare the loops predicted by each method. This is also what the author did in this manuscript as well - I am quoting their own writing here, "This filtering step is necessary to address the candidate peak value as a singular outlier within the neighborhood." Therefore, I do not understand the authors are "irritated" by such approach.
3. The authors of Peakach have released their prediction in 56 Hi-C datasets on their 3D Genome Browser website (http://3dgenome.fsm.northwestern.edu/publications.html), including the ones used in this manuscript. The authors used models trained at different sequencing depths for different datasets. Therefore, I would suggest the authors use this dataset for a fair evaluation.

Regarding Fit-Hi-C, what are the number of peaks the before and after filtering? The author also needs to provide the loop locations so that reviewers can evaluate their claim independently. This information is critical. This manuscript might be helpful for the authors to evaluate Fit-Hi-C (Arya Kaul et al. Nature Protocol 2020).

Finally, the authors need to provide all the predicted chromatin loops in the cell lines as well as loops

predicted by other software used in this manuscript as supplementary materials (loops in Supplementary Table 1).

Level of Interest

Please indicate how interesting you found the manuscript: Choose an item.

Quality of Written English

Please indicate the quality of language in the manuscript: Choose an item.

Declaration of Competing Interests

Please complete a declaration of competing interests, considering the following questions:

- Have you in the past five years received reimbursements, fees, funding, or salary from an organisation that may in any way gain or lose financially from the publication of this manuscript, either now or in the future?
- Do you hold any stocks or shares in an organisation that may in any way gain or lose financially from the publication of this manuscript, either now or in the future?
- Do you hold or are you currently applying for any patents relating to the content of the manuscript?
- Have you received reimbursements, fees, funding, or salary from an organization that holds or has applied for patents relating to the content of the manuscript?
- Do you have any other financial competing interests?
- Do you have any non-financial competing interests in relation to this paper?

If you can answer no to all of the above, write 'I declare that I have no competing interests' below. If your reply is yes to any, please give details below.

I declare that I have no competing interests

I agree to the open peer review policy of the journal. I understand that my name will be included on my report to the authors and, if the manuscript is accepted for publication, my named report including any attachments I upload will be posted on the website along with the authors' responses. I agree for my report to be made available under an Open Access Creative Commons CC-BY license (http://creativecommons.org/licenses/by/4.0/). I understand that any comments which I do not wish to be included in my named report can be included as confidential comments to the editors, which will not be published.

Choose an item.

To further support our reviewers, we have joined with Publons, where you can gain additional credit to further highlight your hard work (see: https://publons.com/journal/530/gigascience). On publication of this paper, your review will be automatically added to Publons, you can then choose whether or not to claim your Publons credit. I understand this statement.

Yes Choose an item.
